# Base-resolution methylation patterns accurately predict transcription factor bindings *in vivo*

**DOI:** 10.1093/nar/gkv151

**Published:** 2015-02-26

**Authors:** Tianlei Xu, Ben Li, Meng Zhao, Keith E. Szulwach, R. Craig Street, Li Lin, Bing Yao, Feiran Zhang, Peng Jin, Hao Wu, Zhaohui S. Qin

**Affiliations:** 1 Department of Mathematics and Computer Science, Emory University, 400 Dowman Drive, Atlanta, GA 30322, USA; 2 Department of Biostatistics and Bioinformatics, Rollins School of Public Health, Emory University, 1518 Clifton Road, Atlanta, GA 30322, USA; 3 Department of Human Genetics, Emory University, School of Medicine, 615 Michael Street, Atlanta, GA 30322, USA; 4 Department of Biomedical Informatics, Emory University, 36 Eagle Row, Atlanta, GA 30322, USA

## Abstract

Detecting *in vivo* transcription factor (TF) binding is important for understanding gene regulatory circuitries. ChIP-seq is a powerful technique to empirically define TF binding *in vivo*. However, the multitude of distinct TFs makes genome-wide profiling for them all labor-intensive and costly. Algorithms for *in silico* prediction of TF binding have been developed, based mostly on histone modification or DNase I hypersensitivity data in conjunction with DNA motif and other genomic features. However, technical limitations of these methods prevent them from being applied broadly, especially in clinical settings. We conducted a comprehensive survey involving multiple cell lines, TFs, and methylation types and found that there are intimate relationships between TF binding and methylation level changes around the binding sites. Exploiting the connection between DNA methylation and TF binding, we proposed a novel supervised learning approach to predict TF–DNA interaction using data from base-resolution whole-genome methylation sequencing experiments. We devised beta-binomial models to characterize methylation data around TF binding sites and the background. Along with other static genomic features, we adopted a random forest framework to predict TF–DNA interaction. After conducting comprehensive tests, we saw that the proposed method accurately predicts TF binding and performs favorably versus competing methods.

## INTRODUCTION

A fundamental goal of functional genomic research is to understand gene regulation. Gene expression can be controlled by epigenetic mechanisms via the coordinated binding of transcription factors (TFs), histone modifications, and DNA methylation (1). An important first step toward deciphering the complexities of gene regulatory networks is detecting the activities of functional elements, such as TF binding sites in the genome.

Advances in high-throughput sequencing technologies such as ChIP-seq (2–4) and ChIP-exo (5) allow the comprehensive genome-wide profiling of protein–DNA binding sites. In recent years, enormous efforts have been made to map TF binding sites under different biological contexts; for example, by consortiums like ENCODE (6) and modENCODE (7). In spite of the successes, the application of ChIP-seq is still limited by the availability of high-quality antibodies and a requirement for fresh cells/tissues. The multitude of distinct proteins makes genome-wide profiling for all of them labor-intensive and costly. Furthermore, individual profiling of TF binding is a challenge in clinical settings because the amount of biological materials available is often limited. For these reasons, developing *in silico* approaches to predict *in vivo* TF binding sites that do not rely on ChIP-seq is desirable.

Traditionally, DNA sequence motifs have been used to predict TF binding (8,9). However, such an approach only works well for proteins with binding motifs that are highly specific. For proteins with weak binding motif patterns, the predictions suffer low specificity. In addition, the DNA motif is insufficient to determine whether a TF will bind to DNA *in vivo*, which means cell type-specific binding cannot be determined; additional information is needed to make that prediction. Recent studies revealed that TF binding is associated with nucleosome positions (10), histone marks (4,11), and hypersensitivity to cleavage by DNase I (12,13). Based on these findings, a number of statistical methods and software tools have been developed to integrate motif information with other data types and genome annotations to achieve better prediction results (10,14–20). All these methods use histone or DNase I data, as well as the genome annotations and DNA motifs for prediction. One of the common limitations is that the histone modification or DNase I hypersensitivity studies require large amounts of fresh starting material (at least from 10^6^ cells). This makes the existing prediction methods (Supplemental Materials Table S1) practically inapplicable to clinical samples.

DNA methylation is an important epigenetic modification with essential roles in many biological processes (21,22). Methylation of cytosine at carbon five (5-methylcytosine, or 5mC) regulates gene expression, determines cell development, and affects numerous disease pathogeneses (22,23). Exploiting next-generation sequencing technologies, a powerful experimental assay called bisulfite sequencing (BS-seq) was developed that measures DNA methylation at base resolution genome-wide (24–26). The experiment starts by treating DNA molecules with sodium, which induces deamination and conversion of unmethylated cytosine to uracil, while methylated cytosine is protected by the methyl group and remains unchanged. The uracil will be amplified as thymine during amplification. The bisulfite-treated and PCR-amplified DNA segments then go through high-throughput sequencing. After alignment and preprocessing, BS-seq data can be analyzed by counting the number of sequencing reads for each CpG site where either a thymine or a cytosine is observed. The count of thymine represents the number of sequenced DNA strands that are unmethylated, and the count of cytosine represents the number of DNA strands that are methylated at this CpG site.

5mC is known to interfere with DNA–protein interactions, thereby directing transcriptional states (27). For example, a recent publication reported that 5mC is strongly correlated with TF binding, where the binding sites are usually hypomethylated (28). Regulation of DNA–protein interactions can occur either through affinity of methyl-CpG-binding proteins for 5mC, or through the refractory effects of 5mC on some DNA–protein interactions. The latter is known to directly influence binding of a number of TFs, such as CTCF (29). Furthermore, more recent observations have implicated the iterative oxidation of 5mC to 5-hydroxymethylcytosine (5hmC), 5-formylcytosine (5fC) and 5-carboxylcystosine (5caC) in pathways that serve to offset 5mC levels and facilitate TF binding (30). All these findings indicate that DNA methylation levels offer clues as to whether TF binding occurred at a particular locus, which may be exploited as an alternative to the DNase I or histone data for the purpose of predicting TF binding *in vivo*. This is important because DNA methylation profiles are more stable and much easier to obtain than DNase I profiles in a clinical setting.

**Figure 1.**
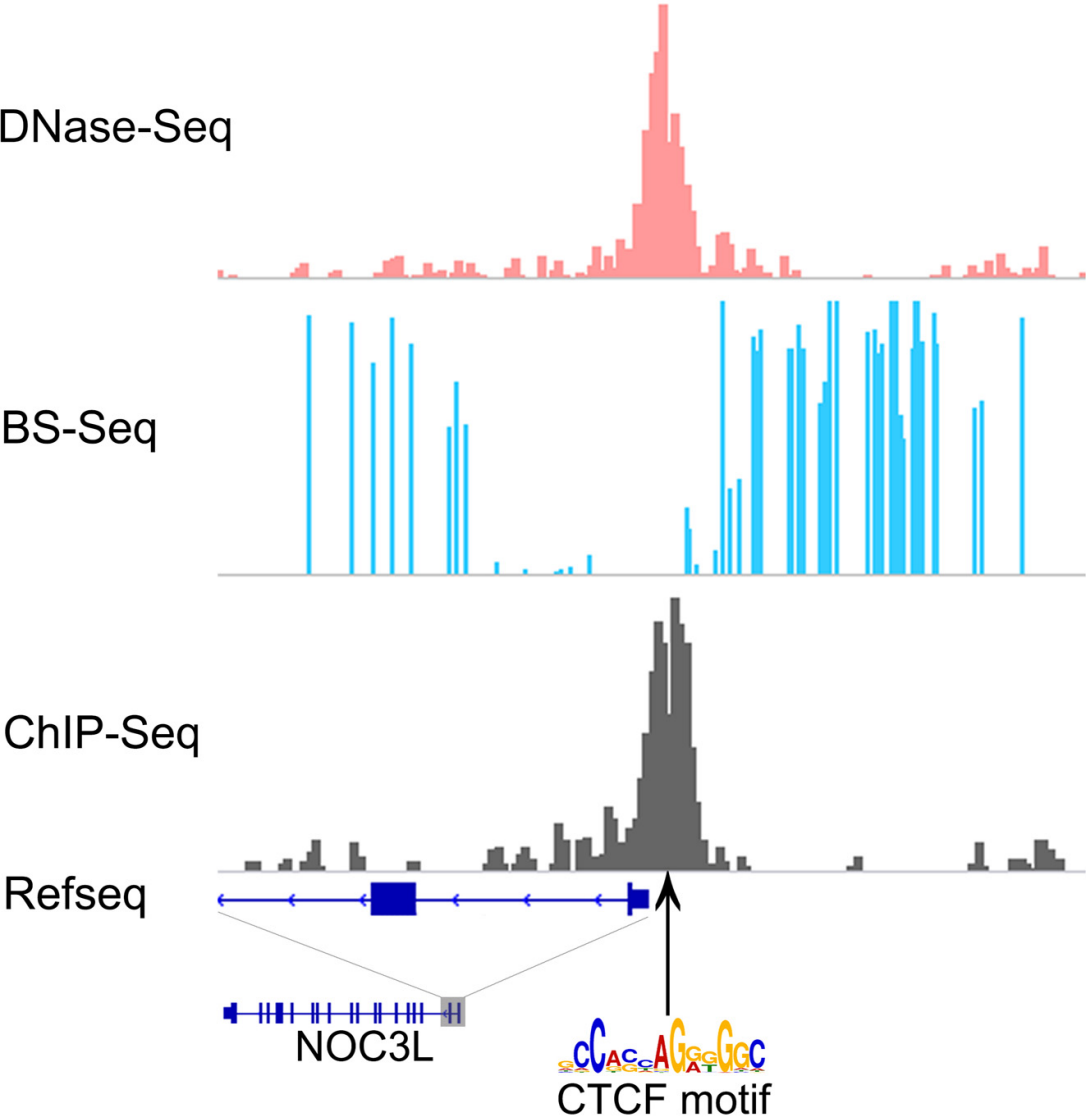
Concordances between ChIP-seq, DNase I and methylation on a genomic locus. A comparative view of DNase I-hypersensitive, methylation (5mC) and ChIP-seq profiles on a genomic locus on chromosome 10. Good concordances are shown between ChIP-seq peaks (used as the gold standard for TF binding), DNase I peaks and methylation ‘dips’. All data shown are from the H1-hESC cell line.

To investigate the viability of this hypothesis, we aligned profiles of DNase I, methylation and TF binding obtained by DNase-seq, BS-seq and ChIP-seq, respectively, in which the ChIP-seq data are used as the gold standard for TF binding. Visual inspection showed there are good concordances between ChIP-seq peaks, DNase I peaks, and methylation ‘dips’. As an example, Figure 1 shows one CTCF binding sites in H1-hESC, which is located at the transcription start site (TSS) of a protein-coding gene. One can clearly see that at the TF binding sites (indicated by ChIP-seq peaks), the DNase-seq data indicates enrichment of DNase I hypersensitivity sites. At the same locations, the DNA methylation levels are altered and show strong hypomethylation.

**Figure 2.**
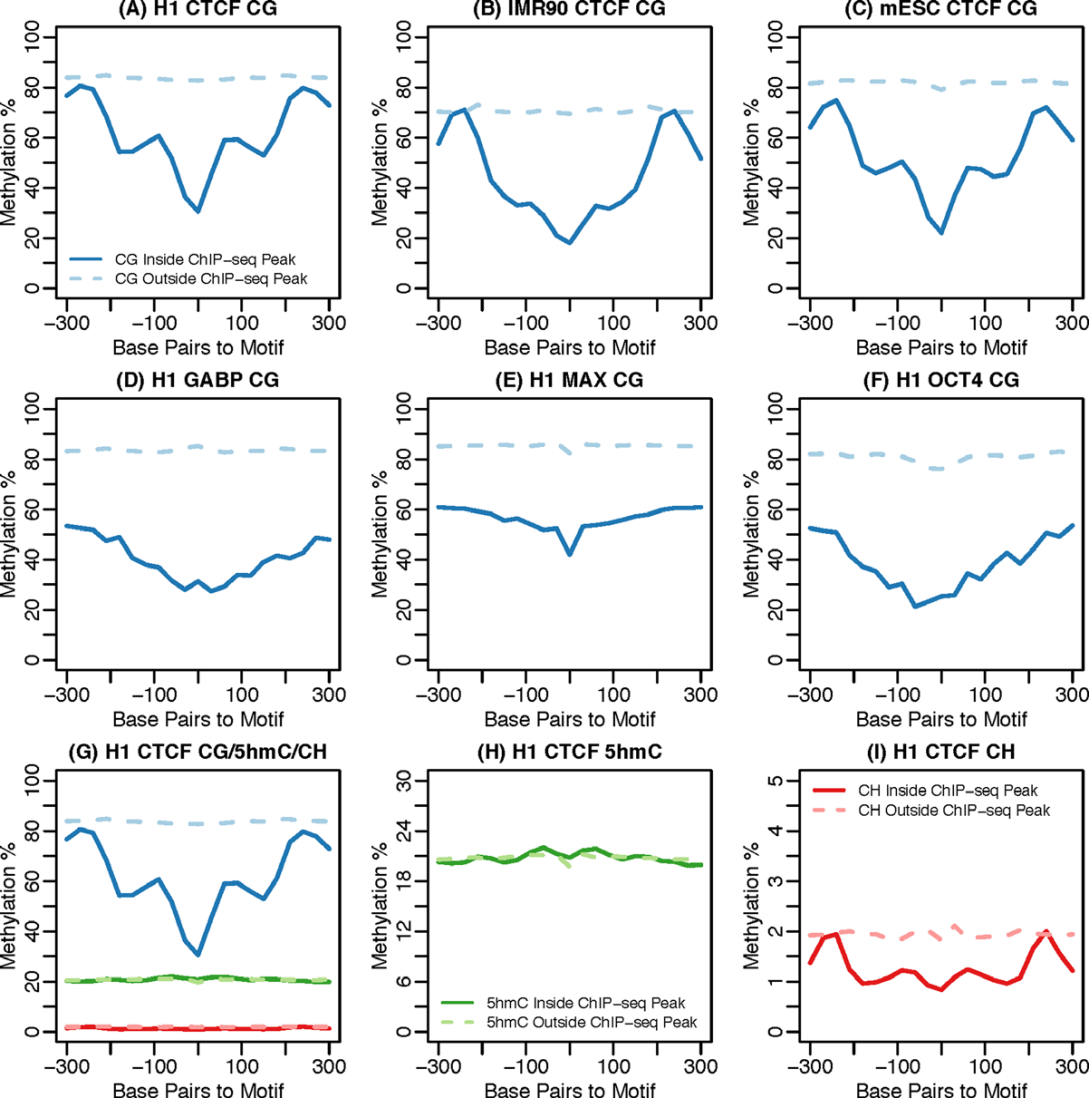
Methylation profiles from different cell lines/TFs/methylation type. Methylation patterns around binding sites of several TFs from different cell lines. Curves represent average methylation (CG/5hmC/CH) levels around ChIP-seq peaks (solid lines) and motif sites without ChIP-seq peaks (dashed lines). (**A**–**C**) CG methylation profiles for CTCF from different cell lines (H1-hESC, IMR90 and mESC). (**D**–**F**) CG methylation profiles for several TFs (GABP/MAX/OCT4) from H1-hESC. (**G-I**) Different types of methylation profiles (CG/5hmC/CH) for CTCF from H1-hESC.

For a more comprehensive view of the methylation profiles around TFBSs, we explored whole-genome BS-seq data from two human cell lines (embryonic stem cell H1-hESC and fibroblast IMR90) and one mouse cell line (embryonic stem cell mESC). We calculated the average methylation levels of three types of methylation: CG methylation (5mC), CG hydroxymethylation (5hmC), and non-CG (CH) methylation around putative TF binding sites (motif sites covered by a ChIP-seq peak) and compared these levels to those from non-TF binding sites (motif site not covered by any ChIP-seq peak). A ‘meta-gene’ style plot is shown in Figure 2, with more such plots shown in Supplemental Materials Figure S1) From these plots, we make three important observations. First, there are differences in the methylation levels between actual TF binding sites and random regions, with 5mC patterns showing the most pronounced difference. Second, the methylation profiles are distinct for different TFs. Third, the methylation patterns for the same TF are similar across cell types. Taken together, these findings indicate that methylation profiles, similar to the DNase-seq data, can be used to distinguish TF binding sites from the genomic background. Despite the empirical evidence connecting methylation level variation and TF binding, how to develop a rigorous statistical approach to quantify the methylation profiles around TF binding sites is non-trivial. Another key question is how to integrate methylation information along with DNA sequence motif and other genomic features in a coherent framework to predict TF binding *in vivo*.

Motivated by these findings, we developed a novel computational approach to predict TF binding. Our method, named Methylphet, is a supervised learning strategy that is able to combine methylation profiles and multiple genomic features to make TF binding predictions. Using ChIP-seq data as surrogates for putative TF binding, we show that Methylphet achieves higher accuracy than prediction method using motif score alone or DNase I profiles. Compared with histone ChIP-seq or DNase-seq, BS-seq can be accomplished using very little material (nanograms of genomic DNA) with highly sensitive bisulfite conversion-based methods, making a prediction method based on BS-seq data a good alternative means for inferring gene regulatory mechanisms from samples in which ChIP-seq and DNase I hypersensitivity studies are not feasible.

## MATERIALS AND METHODS

### Description of the Methylphet method

**Figure 3.**
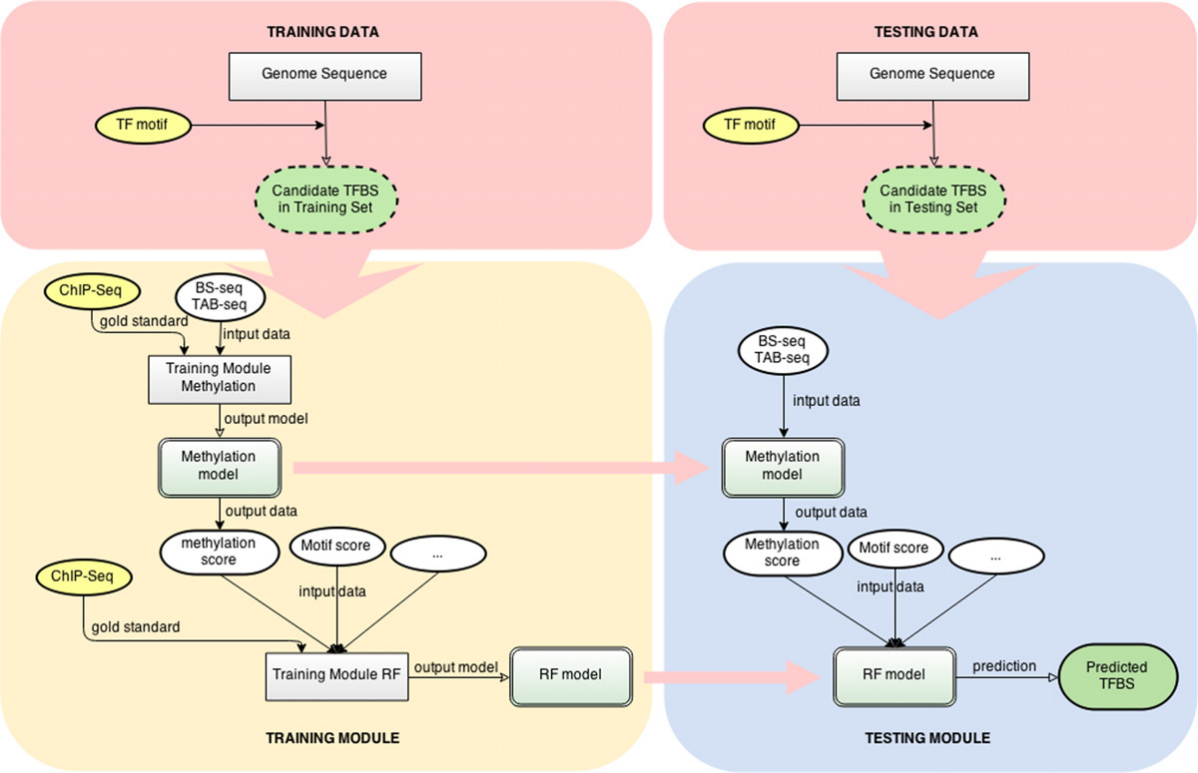
A flow chart for Methylphet method.

The workflow of Methylphet is illustrated in Figure 3. The method consists of candidate site selections, a training module and a testing module. The first step is to identify candidate binding sites by genome-wide motif scan using motif PWMs in both training and testing data. The detailed procedure of selecting candidate sites is provided in the ‘Data and Processing’ section, and summary of candidate sites for the 19 TFs used in this study can be found in Supplementary Table S2-1. Then a predictive model is constructed in the training module, and then the model is put to work for TF binding prediction in the testing module. We use Random Forest (RF) (31) to build the predictive model. RF is an ensemble learning method for classification that recently became popular in genomics because of its flexibility, efficiency and ability to avoid over-fitting. Moreover, RF provides importance measurements for all predictors, which are key to deciding whether to remove an unrelated predictor or add a new promising one.

The required inputs for the training module include the ChIP-seq peak locations (as the gold standard), a set of whole-genome BS-seq data, and other static genomic features, such as DNA motif and evolutionary conservation scores. Optionally, 5-hydroxymethylcytosine (5hmC) data from Tet-assisted BS-seq (TAB-seq) (29) can also be included. The training module contains two steps: the construction of a methylation model and a RF model respectively. Motif information is not used in the methylation model training step, but used in constructing the RF model. With the candidates available, we first identify the putative binding sites (those inside a ChIP-seq peak) from all candidate regions using the gold standard ChIP-seq data. Next in the estimation of methylation models, we characterize the methylation count data in a genomic window around the true TF binding sites as a series of beta-binomial distributions (details are provided in the next section). Then the same procedure is applied to candidate regions without TF binding. At the end of this step, we obtain two sets of beta-binomial distributions for the methylation profiles from TF binding and background regions. For example, means of the beta-binomial distributions represent the shapes of the methylation levels around TF binding or background regions (as shown in Figure 2). Based on the estimated distributions, for each candidate region we compute multiple ‘methylation scores’, which are defined as the likelihood ratios of the site being a true binding site versus being the background. The methylation scores include 5mC scores, CH methylation scores and 5hmC scores if TAB-seq data are available. Next for the training of the RF model, in addition to methylation scores, we also include genomic features, such as motif scores, conservation scores and distance to TSS. A complete list of genomic features used is presented in Supplementary Table S3. Subsequently, the methylation and RF models produced from the training module are employed for prediction. It is important to note that a different predictive model is constructed for each TF due to the TF specificity of the methylation profiles.

### Methylation models

We used the following model to characterize methylation (including 5mC, 5hmC and CH methylation) patterns at a genomic region. Given a candidate site, we treat the motif site as a window, and then add ten 30 bp window to each side. The methylation profiles in these 21 windows are used to capture methylation patterns for TF binding sites and backgrounds. We choose 30 bp as the window size for the sake of balancing the needs of model parameter estimation accuracy and spatial resolution of the methylation profile. To gauge the impact of the window size selection, we repeat the whole analysis procedure using window size of 20 bp and compare the two sets of results. We found that the two sets of results are very similar and the 30-bp results are slightly better overall (Supplemental Materials Figure S11). In the software implementation of Methylphet, we provide option for user to specify the window size.

Inside each window, if there was at least one CG dinucleotide covered by at least one read (either methylated or not), we recorded the total number of methylated and unmethylated reads. Assume there are *n* candidate sites. In the *j*th window ($j = 1,2, \ldots ,21$) of the *i*th candidate site ($i = 1,2, \ldots n)$, we used $x_{ij}$ and $y_{ij}$ to denote the number of methylated and unmethylated reads and let $n_{ij} = x_{ij} + y_{ij}$.

Similar to (32), we used a beta-binomial compound distribution to model the count data from BS-seq. The counts, given underlying ‘true’ methylation levels, are assumed to follow a binomial distribution:

\begin{equation*} x_{ij} |n_{ij} ,p_j \sim {\rm Binom}\left( {n_{ij} ,p_j } \right),i = 1,2, \ldots n;j = 1,2, \ldots 21.\end{equation*}

The methylation levels $p_j$'s are assumed to follow a beta distribution, but with different parameters at TF binding sites and background. When there is no TF binding at a candidate site, the methylation levels from all 21 windows are assumed to be identical and similar to those from the genomic background (close to fully-methylated). Thus, we assume $p_j$'s follow the same beta distribution. For candidate sites that are bound by TFs, we found (Figure 2) that the methylation levels are different at different windows, e.g. methylation levels dip toward the motif site from both directions. Therefore, we assume that each $p_j$ follows a different beta distribution. Defining indicator $z_i$ to denote binding ($z_i = 1$) or not ($z_i = 0$) for candidate site *i*, we have

\begin{equation*} p_j |z_i = 1\sim {\rm Beta}\left( {\alpha _j ,\beta _j } \right),\;p_j |z_i = 0\sim {\rm Beta}\left( {\alpha ',\beta '} \right).\end{equation*}

For quality control purposes, we removed all windows with less than five total reads and candidates within CpG islands from the training set and used the method of moment (MOM) to estimate parameters $\alpha _j ,\beta _j$ and $\alpha \prime ,\beta \prime$. More details about this can be found in the Supplemental Materials. With parameters estimated at each motif site, we calculated the likelihood ratio comparing the two methylation patterns (TF binding or no binding) as methylation score $\lambda _i$ for the ith candidate sites in test data:

\begin{equation*} \lambda _i = \mathop \sum \limits_{j:\# {\rm of\,CG} >0}^m \log \left( {\frac{{p\left( {x_{ij} {\rm |}n_{ij} ,z_i = 1} \right)}}{{p\left( {x_{ij} {\rm |}n_{ij} ,z_i = 0} \right)}}} \right)\end{equation*}

Higher methylation scores indicated stronger evidence for a candidate site to have TF binding. The same procedure was applied to obtain CH methylation scores, as well as 5hmC scores if whole-genome TAB-seq data were available.

### Other genomic features

Other genomic information used in the predicting model included: sequence conservation, distance to TSS, overlap with repetitive region, and other genomic features. Conservation scores were downloaded from the UCSC genome browser, hg18 phastCons44way table. Repeat masker, which marks the repetitive regions, was downloaded from the UCSC genome browser. We also calculated the nearest distance between candidate binding sites. For other genomic features, we used a binary indicator (0 or 1) to show if the motif overlapped with: TSS, TES, exons, introns, or CpG islands, and the distance to TSS. All the genomic Feature annotations were calculated using R and Bioconductor.

### Prediction

Several supervised learning approaches were investigated, and RF performed the most accurately and robustly among all the approaches (Supplementary Figure S8). Hence, results were demonstrated using RF, which was achieved with R package randomForest (33).

In the RF, a binary classification model was trained using methylation score together with genomic features. In each trained model, the importance of input features was assessed using Gini gain importance. The number of trees used in the model was determined by the stability of out-of-bag error (see Supplemental Materials 6.3). The predicting result is represented by the probability of getting a vote from the randomly generated classification tree for each class. The predicting performance was evaluated using the ChIP-seq peaks as the gold standard. ROC based on the class probability and the gold standard was computed to show the overall predicting performance of our method.

### Data and processing

#### 5mC data from bisulfite sequencing (BS-seq) studies

The BS-seq data from human embryonic stem-cell (hESC) lines H1-hESC and IMR90 were downloaded from Gene Expression Omnibus (GEO) with ID GSE16256 (24). The 5mC BS-seq data from the mouse embryonic stem-cell (mESC) line was downloaded from GEO with ID GSE30202 (28). The 5mC BS-seq data from the mouse dentate gyrus (DG) cells was downloaded from GEO with ID GSM1263221 (34)

#### Bisulfite-seq paired-end read processing and methylation calling

Paired-end reads were first pre-processed to remove adapter sequences, as well as low-quality sequence on both the 3′ and 5′ ends using Trimmomatic 0.20 (35), with the following parameters: LEADING:3 TRAILING:3 SLIDINGWINDOW:4:15 MINLEN:36. This was followed by *in silico* conversion of each C to T (Read 1) and each G to A (Read 2). Preprocessed reads were then aligned to both C-to-T and G-to-A converted chromosomes that were computationally derived from NCBI mm9 genomic sequence using Bowtie 0.12.9 (36) (-m 1 -l 30 -n 0 -e 90 -X 550). Reads mapping to both genomes were discarded and non-aligned pairs were reprocessed as single-end data using the same alignment parameters. For both paired-end and single-end alignments, only uniquely mapping reads were retained, and PCR duplicates were removed using MarkDuplicates (Picard Tools 1.82). To avoid counting reference positions covered by overlapping paired-end reads, overlapping regions were clipped, keeping the region of the overlap with higher quality. The original computationally converted C's and G's were reverted, and for each reference cytosine position the number of C reads and T reads were counted using SAMTools mpileup. We kept the number of 5mC reads as well as total read coverage at each CG dinucleotide where 5mC is present for the processed data.

#### 5hmC data

The base-resolution maps of 5hmC in human and mouse ES cells were generated previously (29). We used the same procedure described above to process 5hmC data and call methylation.

#### DNase data

The DNase I cutting sites were derived from the ENCODE dataset. We downloaded the Human H1-hESC DNase sequencing alignment files from ENCODE Crawford–Duke chromatin Map dataset via ENCODE Data Coordination Center (DCC); we downloaded the mouse mESC DNase sequencing alignment files from ENCODE Uw Dnase dataset from ENCODE DCC; DNase-seq alignment files for the IMR90 cell line were first downloaded from ENCODE Duke OpenChromDnase dataset on ENCODE DCC, and then converted to the hg18 coordinate system using liftover (37).

#### ChIP-seq data

The ChIP-seq mapping results for all the TFs in H1-hESC cells, IMR90 cells, and CTCF in mouse mESC Cells were downloaded from UCSC ENCODE collection (38). We performed the ChIP-seq experiment on mouse mESC for OCT4.

#### ChIP-seq experiment

ChIP-seq experiments were performed following the protocol from the laboratory of Richard M. Myers (http://myers.hudsonalpha.org/documents/Myers%20Lab%20ChIP-seq%20Protocol%20v041610.pdf). Briefly, 2 × 10^7^ mouse ES cells were cross-linked with 1% formaldehyde at 25°C for 10 min and sonicated to generate chromatin fragments of 100–500 bp. Chromatin fragments from 2 × 10^7^ cells were immunoprecipitated using OCT4 antibody (Abcam ab8895). ChIP-seq library construction and Illumina sequencing were performed following the manufacturer's instructions.

#### ChIP-seq data processing

For bam files that listed genomic coordinates in hg19, we first converted them to genomic coordinates in hg18 using liftover (37). We next used HPeak (39) for peaking calling. Peak intersections of biological replicates are retained and employed for model training to maintain enhanced ChIP signal strength.

#### Selection of candidate regions

The candidate TF binding regions are selected based on sequence motif scores. The position-specific weight matrices (PWM) for CTCF, MAX, SIX5, USF1, BCL11A, EGR1, NANOG, RAD21, RFX5, SRF, USF2, GABP, NRSF, YY1, CJUN, JUND, OCT4 and TCF12 were downloaded from JASPAR (40) and factorbook (38). We used PWM matching functions from Bioconductor package ‘Biostrings’ to scan the entire genome to identify candidate sites for TFBS. The cutoff for candidate sites leaves between 200 000 and 600 000 candidate sites for most TFs (more details can be found in Supplemental Materials).

### Data access

ChIP-Seq data in mESC have been submitted to GEO (GEO accession number GSE65093).

## RESULTS

### TF binding prediction results

**Figure 4.**
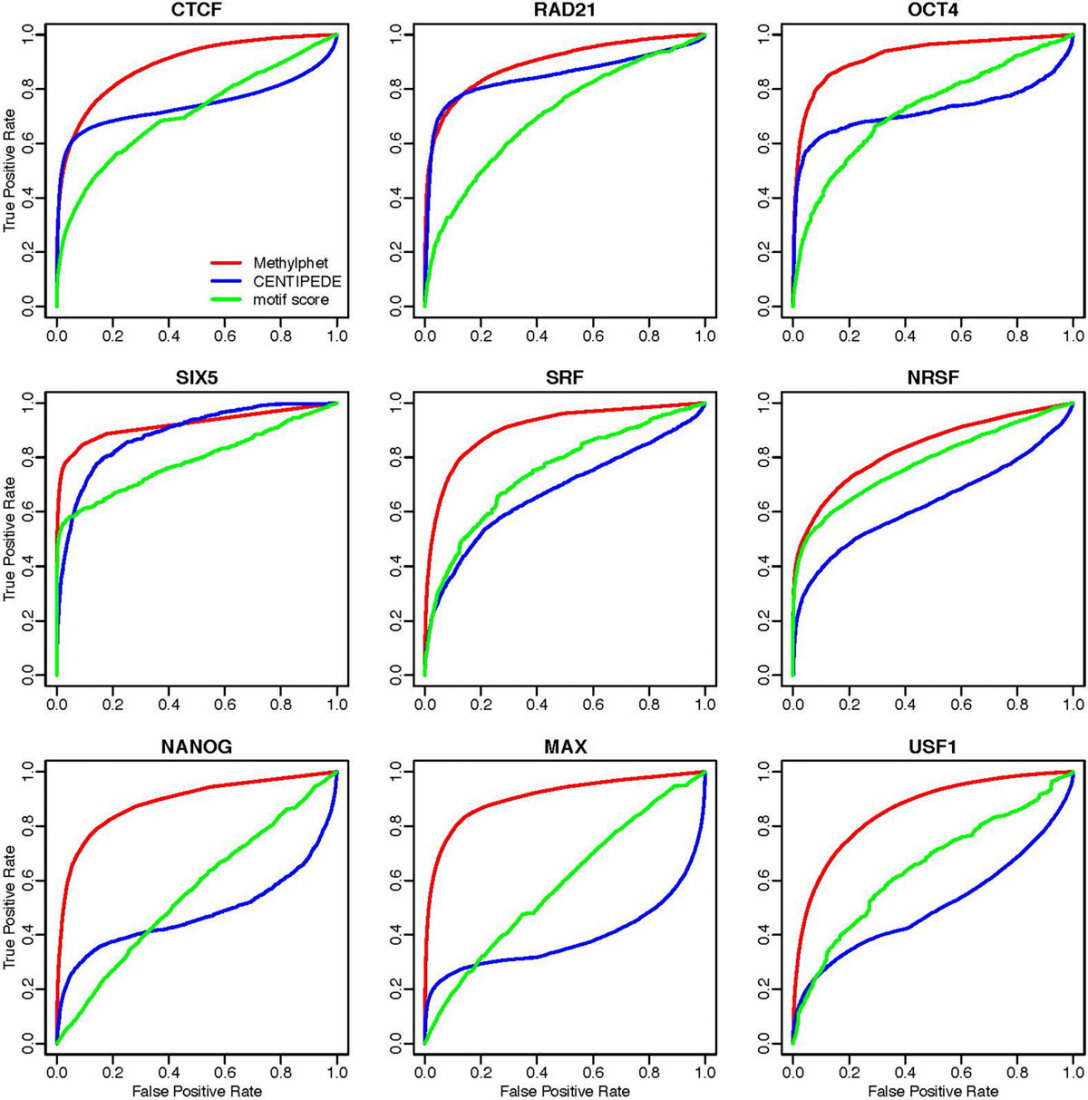
TF binding prediction results for H1-hESC cell line. ROC curves for Methylphet, CENTIPEDE, and motif score are shown by red, blue, green lines, respectively. Prediction results were generated by randomly splitting the dataset into training set and testing set of equal size for the H1-hESC cell line. Methylphet robustly provides better predicting performance for different transcription factors. ROC curves for other factors in H1-hESC, IMR90, and mESC are shown in Supplementary Figures S1–S3.

**Figure 5.**
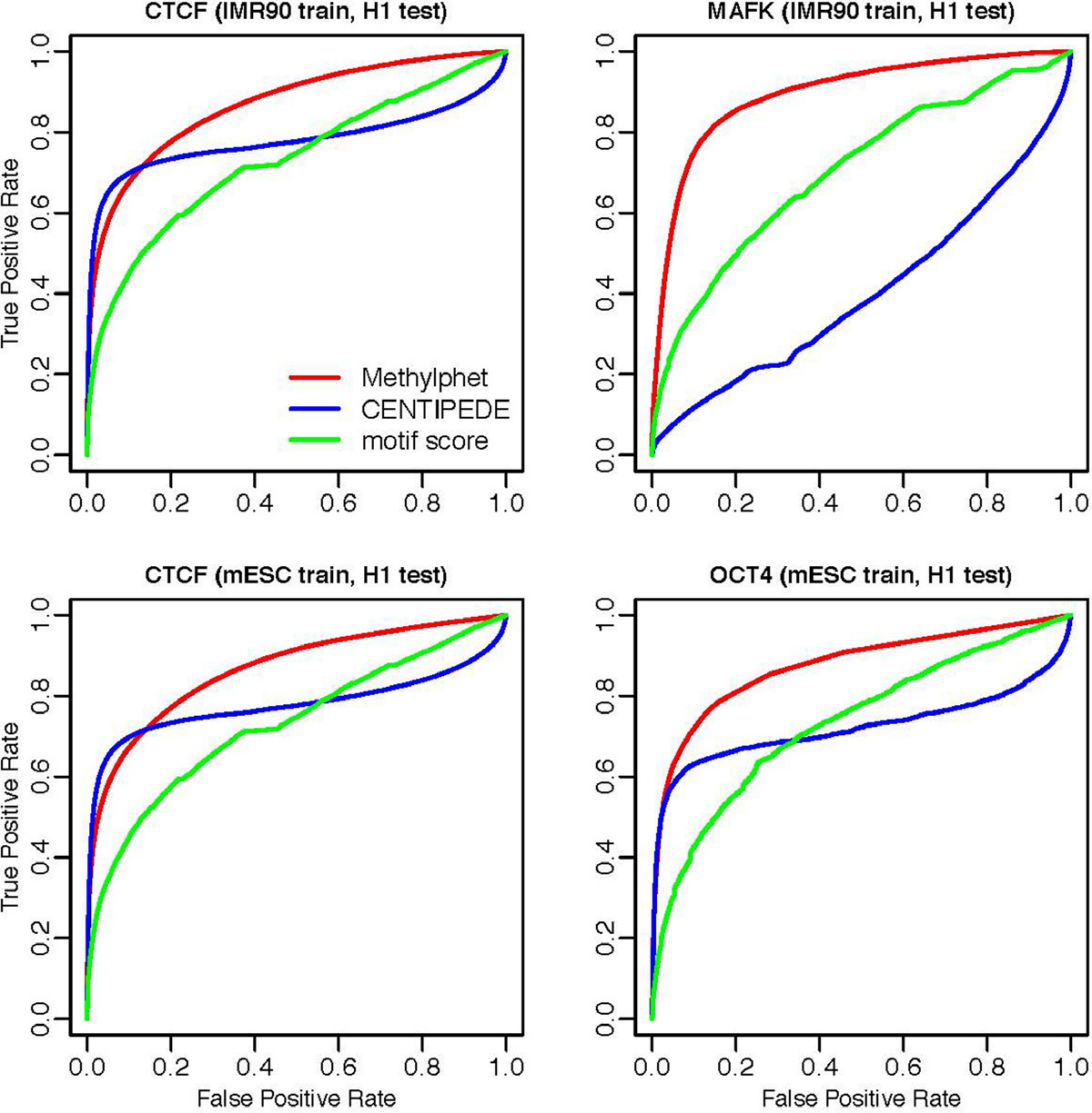
Cross-sample TF binding prediction results. Upper two figures are cross-cell line predictions between IMR90 and H1-hESC; lower two figures are cross-cell line predictions between a mESC cell line and H1-hESC. The results demonstrate that Methylphet generally achieves more robust and precise prediction.

We conducted extensive real data analyses to evaluate the performance of Methylphet. In total, we performed TF binding prediction of 19 TFs for human embryonic stem cell line H1-hESC and five TFs for human fibroblast cell line IMR90. We randomly split candidate sites into equal-sized training set and testing set. Prediction performance is evaluated on the testing set only. We compared the prediction performance of Methylphet with CENTIPEDE (16), which is a widely used unsupervised method using DNase-seq data to predict TFBS. CENTIPEDE takes other genomic features to construct the prior for TF binding prediction. For a fair comparison, we fed the same set of non-cell-type-specific, static genomic features, such as conservation score, motif score, etc. to both CENTIPEDE and Methylphet. Besides those, DNase data were used in CENTIPEDE, and methylation data were used in Methylphet. We did not include methylation data for CENTIPEDE, nor did we include DNase data for Methylphet. We also compared with predictions using sequence motif only (candidate regions are ranked by their motif scores). Receiver Operation Characteristic (ROC) curves are used to represent the overall predicting performance of each method (Figures 4 and 5). Considering that a majority the candidate sites are negative for some TFs (Supplementary Table S2-1), we also generate precision-recall curves for performance evaluation (Supplementary Figures S9 and S10).

Figure 4 shows the ROC curves for nine different TFs in H1-hESC. These extensive real data analyses show that Methylphet robustly outperforms CENTIPEDE and the motif-score-only method. To be more specific, Methylphet outperforms motif-score-only for all TFs in all cell lines. This is expected since Methylphet effectively combines motif score and information from methylation profiles and other genomic features. Compared with CENTIPEDE, which also considers motif score and other genomic features, Methylphet also outperforms significantly in all TFs. Although CENTIPEDE and Methylphet perform similarly for CTCF and RAD21 when the false-positive rate (FPR) is small (<0.1), Methylphet outperforms CENTIPEDE after the FPR is >0.1. Similar results for other TFs in H1-hESC, more results in mESC and IMR90 cell lines are shown in Supplementary Figures S4–S6. In order to demonstrate the robustness of the performance, we repeat the testing for 10 times. The boxplots of the 10 area under the ROC curves values (Supplemental Materials, Figure S12) show that Methylphet robustly outperforms motif score and CENTIPEDE.

In addition to the difference in information sources, the underlying strategy of Methylphet, which is an ensemble learning approach, is also different from that of CENTIPEDE, which is a mixture model type of approach. It is of interest to find out whether the source of data, or the underlying method is the major contributor of the performance improvement of Methylphet. In order to answer the above question, we replaced the methylation scores with the DNase scores obtained from CENTIPEDE in the RF of Methylphet and compare that to Methylphet as well as CENTIPEDE. Results are summarized in Supplementary Figure S13, which show that in most cases, RF with methylation performs the best, followed by RF with DNase data and then CENTIPEDE. Our comparison results between RF with methylation data versus RF with DNase data seem to suggest that both data source (methylation data versus DNase data) and method used (RF versus mixture model) contribute to the performance improvement of Methylphet over CENTIPEDE. However, it is also possible that the statistical model, not the data source used, made the difference. Therefore, an alternative model for DNase with RF could change, and potentially improve the predicting ability of DNase data.

The advantages of RF, an ensemble learning approach, over the mixture model type of approach adopted by CENTIPEDE can be attributed to two factors. First, due to the high variability among TFs, a supervised learning approach like RF is more robust. On the other hand, an unsupervised mixture model approach may fail in adverse situations. As an example, the EM algorithm (41) fails to converge when the proportion of true positives in the candidate sites is low, which often occurs for TFs with shorter motifs and fewer putative binding sites. Second, RF does not assume independence among the predictors, as does CENTIPEDE. Our experience with CENTIPEDE is that the final results are often dominated by the DNase-seq data. Since the genomic features are diverse and many of them are highly correlated, an RF model can better use the integrated information from predictors.

### Cross-sample TF binding prediction results

We further tested the predictive accuracy when training and testing data were from different samples. Our approach will be most attractive if the model trained in one cell type can produce robust prediction in a different cell type or sample; from Figure 2, this seems plausible since we saw that the methylation pattern is consistent across cell lines for the same TF.

To verify this, we conducted tests in which we trained the Methylphet model using data from IMR90 and mESC cell lines, and then applied the model in a different cell line, H1-hESC, for prediction. Figure 5 shows the ROC curve for predicting CTCF, OCT4 and MAFK binding sites with the cross-cell-line-trained model. For MAFK (model trained on IMR90) and OCT4 (model trained on mESC), Methylphet outperforms CENTIPEDE significantly. For CTCF on H1-hESC (model trained on IMR90 and mESC, respectively), Methylphet outperforms CENTIPEDE after the FPR is >0.15, although CENTIPEDE performs slightly better before that. In terms of overall area under the curve, Methylphet is superior in all TFs. These results demonstrate that Methylphet achieves robust and precise prediction when the model trained in a different cell line, showcasing the broad utility of our method.

### Cross-TF prediction results

We further investigated the TF-specificity of Methylphet model by cross-TF training and predicting (more details in Supplemental Materials, Section 8.6). This result (Supplementary Figure S15) shows that even though cross-TF prediction is possible, TF-specific Methylphet model provides the best results. The methylation profile and other genomic characteristics of TF binding are important in the Methylphet model and better to be modeled in a TF-specific manner.

### Experimental validation in mouse dentate gyrus cells

We performed NRSF binding site prediction in mouse dentate gyrus (DG) cells using Methylphet model trained from mES data. Because NRSF ChIP-seq data in mouse DG cells are not available, we performed qPCR in randomly selected sites as validation. Ten positive and ten negative sites are randomly selected from top 1000/bottom 1000 Methylphet-predicted binding sites respectively. Then five positive and five negative sites have suitable qPCR primers were tested (details in Supplemental Materials, section 8.5). Fold enrichment is calculated on both positive sites and negative sites in order to compare the prediction performance. Among the selected sites, we can see clear enrichment inside positive predicted sites compared to negative predicted sites (Supplementary Figure S14). Detailed information about the selected sites are listed in Supplementary Table S3 in Supplemental Materials.

### Contribution of different features in Methylphet

**Figure 6.**
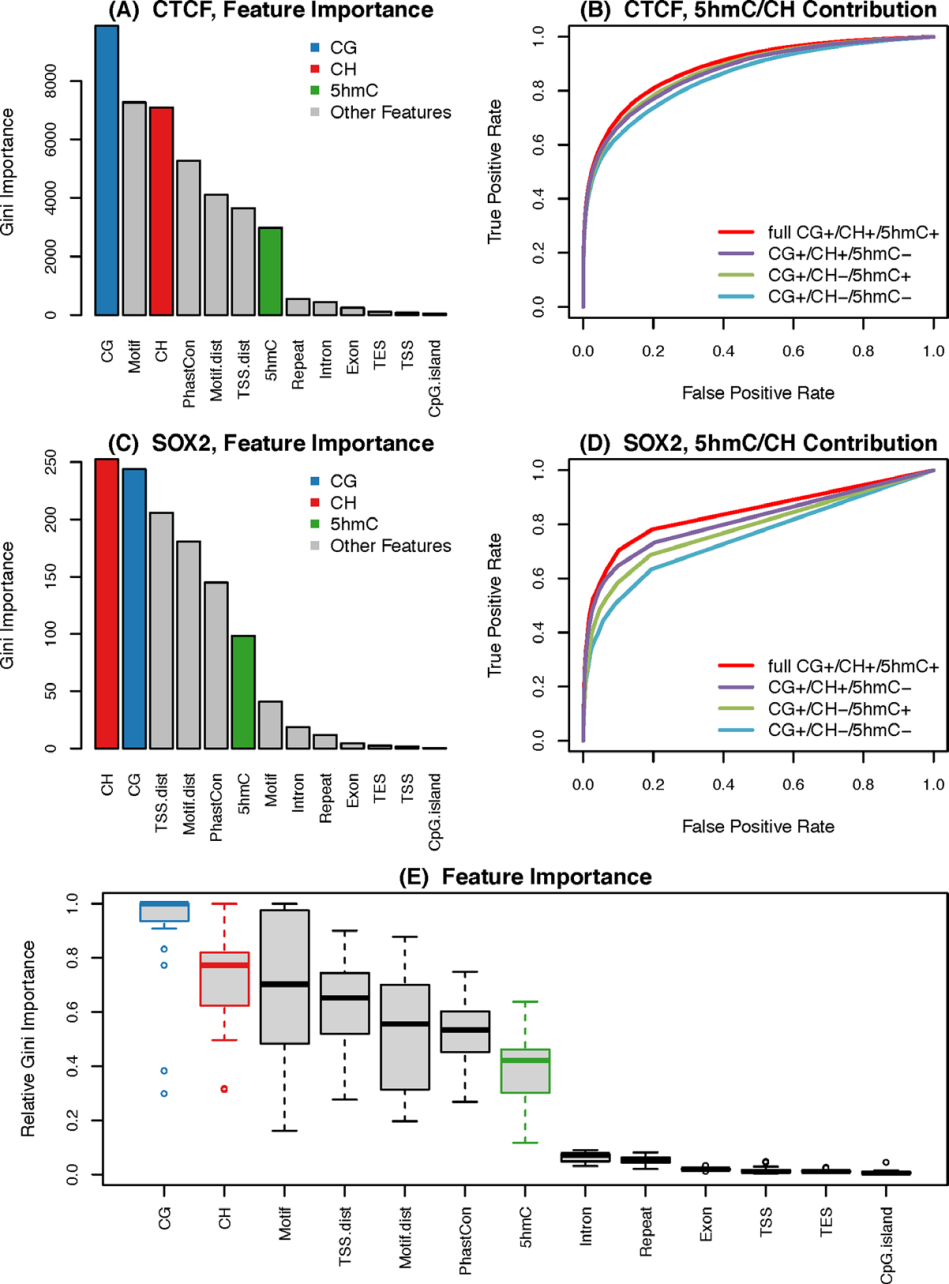
Relative predictive power of genomic features used in Methylphet. (**A**) and (**C**) show the contribution of each feature in RF. (**B**) and (**D**) show the ROC curves with or without adding CG methylation and CH methylation information. Among them, (A) and (B) were generated using CTCF TFBS prediction results in H1-hESC; (C) and (D) were generated using SOX2 TFBS prediction results in H1-hESC. (**E**) Boxplot of Gini importance for each feature used in RF.

It is important to understand the relative predictive power of methylation levels and other genomic features used in Methylphet. We present the Gini importance (Supplemental Materials 6.2) of predictors in the RF model for CTCF and SOX2 using bar plots in Figure 6A and C. Gini importance is the measurement of classification efficiency for each feature, which is defined as the level of decrease in the class impurity. Please see the Supplemental Materials 6.2 about the details of feature importance evaluation. Similar figures for other TFs are provided in Supplemental Figure S7. Based on these analyses, we found that 5mC scores play the most important role in predicting CTCF binding, whereas the CH score is the most important predictor for SOX2. Motif is the second most important for CTCF, but its importance is very low for SOX2. This is because the SOX2 motif has much lower specificity compared to the CTCF motif. For both TFs, CH and 5hmC methylations play rather important roles. We compared the predictive performances with or without 5hmC and CH methylations. Figure 6B and D shows the ROC curves from such comparisons. These results demonstrate that including both 5hmC and CH methylation scores in the model improves the prediction power. Figure 6E shows the distributions of Gini importance of each predictor across all TFs we tested. We can see clearly that the 5mC score contributes the most on average among all features, next being the CH and motif scores, followed by sequence conservation and distance to the closest TSS.

### Comparison with other predicting tools and other machine learning methods

We chose Random Forest as the ensemble algorithm to integrate all the features. During the RF model construction, one single variable was used at a time, and by integrating this information after sampling, it can give an automatic measure of feature importance. This is important since our work requires integrating different types of information and evaluating feature importance. We also compared with other popular supervised machine learning tools, such as Neural Network (42), SVM (43) and adaBoost (44). Supplemental Figure S8 shows the ROCs of the methods compared. In general, we can obtain reasonably good results with all these choices because of the rich information in the methylation scores and other genomic features. Among all the methods we saw robust performance from RF across all the TFs. Even though the predicting result is not sensitive to model selection, we prefer RF for its additional advantages, such as its efficiency on large datasets, ability to avoid over-fitting, and its inherently non-parametric structure. In addition, it can provide more details in the importance of features without extra cost. The evaluation of Gini importance is done as the learning goes, which lead to one of the major discoveries in our study that 5mc and 5hmc profile can contribute as the top predictor in Methylphet.

### Description of the software

R package Methylphet is freely available from https://github.com/benliemory/Methylphet and will be submitted to Bioconductor (45) soon. Methylphet accepts 5mC, 5hmc and CH methylation profiles individually or in combination. As the example in the package shows, training about 7000 candidate sites and predicting on about 10 000 candidate sites with both CG and CH information takes less than one minute on a MacBook Pro laptop computer with 2.7 GHz i7 CPU and 16G RAM. Training time varies depending on number of candidate sites. For most of the cases in this study, training time is <30 min.

## DISCUSSION

In this work, we developed Methylphet, a novel computational method and software package to predict TF binding using a combination of methylation profiles and genomic features. The idea is based on the observation that *in vivo* TF binding events often co-occur with altered methylation levels. Methods for *in silico* prediction of TF binding using epigenetics data have been proposed before, mostly based on histone ChIP-seq or DNase-seq data. Our method exploits methylation data instead, which is much easier to collect experimentally. In this respect, our method provides a more practical means of *in silico* TF binding prediction and will be more useful in the clinical setting.

We show that Methylphet performs very well in the cross-sample and even cross-species predictions. These results imply that a predictive model trained under a certain biological context can be applied for prediction in different samples, which is important because it indicates that the model building procedure (which is the most time consuming) only needs to be performed once, and then the model can be applied elsewhere for the same TF. It is important to note that the predictive models are TF-specific, i.e. each TF will have its own model. This is because around the binding sites of different TFs, both the methylation patterns and the genomic features are different (Figure 2).

Disruption of epigenetic processes is known to contribute to the pathogenesis of multiple human diseases. For example, aberrant epigenetic modifications occurring at the earliest stages of neoplastic transformation are believed to be an essential player in cancer initiation and progression (46,47). Using our method, a change of epigenetic status, particularly DNA methylation status, at a given locus could imply dynamics of *in vivo* TF–DNA interactions. Advances in epigenetics have not only offered a deeper understanding of the mechanisms underlying disease pathogenesis, but have also allowed the identification of putative epigenetic biomarkers for early detection and diagnosis. Nevertheless, it would be very challenging to collect patient tissues/cells that are fresh enough to perform chromatin immunoprecipitation or DNase I-hypersensitive assays. However, DNA methylation analyses could be performed routinely with clinical samples. So, the development of a DNA methylation-based *in vivo* TF–DNA interaction predicting algorithm is critical for uncovering effective biomarkers for human diseases (48).

One constraint of the application of the method is that whole-genome BS-seq experiment is very expensive. However, with the predictive model pre-built from public data, it is possible to use BS-seq data from selected regions (such as reduced representation of BS-seq or RRBS (49)) for binding prediction. Such an approach, although the prediction will not be genome-wide, still provides valuable information at important regions. Potentially, with small modifications of the methylation model, data from methylation microarrays can be used for binding prediction. This will be our research plan in the near future.

Unlike plant genomes, where enzymes for generating and erasing CH methylation have been well characterized (50), CH methylation in mammalian genomes has not been studied extensively until recently. Recent whole-genome bisulfite sequencing revealed that CH methylation is abundant in hESCs and hiPSCs, as well as brain (25). In brain, CH methylation accumulates during neuronal maturation, suggesting a potential role for CH methylation in normal brain function (34). The role of CH methylation in gene regulation remains elusive. Our analyses presented here suggest that CH methylation is among the best predictors of *in vivo* TF–DNA interactions along with 5mC, pointing to an active role for CH methylation in gene regulation. It is possible that the coordination between CpG and CH methylations regulate the dynamics of TF–DNA interaction *in vivo*.

5hmC shares similar characteristics with 5mC data. Since our model is very robust to capture the methylation pattern between TF binding sites and non-binding sites, we extended our model to summarize 5hmC data to calculate 5hmC score. Although 5hmC data are far more sparse than 5mC data, they could provide additional information to predict TF binding sites. Figure 6 shows the ROC curve with and without the 5hmC data. (More results on other TFs can be found in Supplemental Materials Figure S7.)

Beyond TF binding, there are other genomic features that are also related to methylation, such as active/poised transcription, active/poised enhancers, RNA splicing and chromosomal translocation. In the future, we could potentially adapt our machine learning algorithm to detect other genomic elements of interest that play a regulatory role. It would be highly interesting to see whether there are any methylation signatures associated with these events.

## SUPPLEMENTARY DATA

Supplementary Data are available at NAR Online.

SUPPLEMENTARY DATA
