# Supplementary material for: Base-resolution methylation patterns accurately predict transcription factor bindings in vivo
Source: Nucleic Acids Res. 2015 Feb 26;43(5):2757–66. doi: 10.1093/nar/gkv151 (PMC4357735; doi:10.1093/nar/gkv151)
Supplement: SUPPLEMENTARY DATA [file supp_43_5_2757__index.html]

Base-resolution methylation patterns accurately predict transcription factor bindings in vivo — SUPPLEMENTARY DATA 

# Base-resolution methylation patterns accurately predict transcription factor bindings *in vivo*

## SUPPLEMENTARY DATA

**Files in this Data Supplement:**

- Supplementary Material
