# Supplementary material for: Base-resolution methylation patterns accurately predict transcription factor bindings in vivo
Source: Nucleic Acids Res. 2015 Feb 26;43(5):2757–66. doi: 10.1093/nar/gkv151 (PMC4357735; doi:10.1093/nar/gkv151)
Supplement: SUPPLEMENTARY DATA [file supp_gkv151_nar-02908-n-2014-File008.pdf]

Supplementary Material for:

Base-resolution methylation patterns accurately predict transcription factor bindings in vivo

Tianlei Xu[1,3] , Ben Li[1], Meng Zhao[1], Keith E. Szulwach[2], R. Craig Street[2], Li Lin[2], Bing Yao[2], Feiran Zhang[2], Peng Jin[2,]*, Hao Wu[1,]* , and Zhaohui Qin[1,]*

[1] Department of Biostatistics and Bioinformatics, Rollins School of Public Health, Emory University. 1518 Clifton Road. Atlanta, Georgia 30322. USA

[2] Department of Human Genetics, Emory University, School of Medicine, 615 Michael Street. Atlanta, Georgia 30322. USA

[3] Department of Mathematics and Computer Science, Emory University, 400 Dowman Drive. Atlanta, Georgia 30322. USA

* To whom correspondence should be addressed. Tel: +1(404) 712-9576; Fax: (404)727-1370; Email: zhaohui.qin@emory.edu
* Correspondence may also be addressed to Hao Wu. Tel: +1(404) 727-8633; Fax: (404)727-1370; Email: hao.wu@emory.edu
* Correspondence may also be addressed to Peng Jin. Tel: +1 (404) 727-3729; Fax: (404)727-5408; Email: peng.jin@emory.edu

1. A brief summary of existing TF binding prediction methods 2
2. Candidate TFBS selection 3
3. Methylation profile 8
3.1 Methylation profile (CG, 5hmC, CH) in H1-hESC. 8
3.2 Methylation profile (CG, CH) in IMR90. 13
3.3 Methylation profile (CG, 5hmC and CH) in mESC 15
4. Methylphet Model 16
5. Methylphet Performance 18
6. Features in Methylphet 21
6.1 Features Description 21
6.2 Evaluation of Feature Importance 21
6.3 Model convergence and tree number 22
7. Comparison with other machine learning algorithms 42
8. Model evaluation 43
8.1 Precision-recall curves v.s. ROC curves 43
8.2 Choice of bin sizes in constructing methylation model 45
8.3 AUC result for cross-validation 48
8.4 Comparison of different cell-type-specific features 49
8.5 qPCR of selected Methylphet-predicted sites 50
8.6 Cross-TF prediction result. 52

1. A brief summary of existing TF binding prediction methods

Publication	Data used	Method	Software
He et al. (2010)	Nucleosome-resolution histone ChIP-Seq	Dynamics of nucleosome occupancy and motif	N/A
Won et al. (2010)	Histone ChIP-seq	HMM with supervised learning	Chromia
Ramsey et al. (2010)	Histone acetylation ChIP-seq, nucleosome occupancy, genome	Weighted sum of scores. Model training by supervised learning	RamseyHAc2010
Pique-Regi et al. (2011)	DNase I + genome	Two-component mixture model, EM.	CENTIPEDE
Cuellar-Partida et al. (2012)	DNase I, histone ChIP-seq	Epigenetic data as prior, use motif to predict.	FIMO, part of MEME
Arvey et al. (2012)	Histone ChIP-seq + DNase I	SVM	N/A
Ji et al. (2013)	Nine histone ChIP-seq	PCA-type unsupervised learning.	dPCA
Rajagopal et al. (2013)	Histone ChIP-seq	Random Forest, supervised learning	RFECS

Table S1. A brief summary of existing *in silico* TF binding prediction methods. These studies showed that the TF binding is associated with nucleosome positions (He et al. 2010), histone marks (Heintzman et al. 2007; Robertson et al. 2007), and hypersensitivity to cleavage by DNase I (Bernat et al. 2006; Hesselberth et al. 2009). Based on these findings, a number of statistical methods and software tools have been developed to integrate motif information with other data types and genome annotations, and achieve better prediction results (He et al. 2010; Ramsey et al. 2010; Won et al. 2010; Pique-Regi et al. 2011; Arvey et al. 2012; Cuellar-Partida et al. 2012; Ji et al. 2013; Rajagopal et al. 2013).

2. Candidate TFBS selection

Candidate transcription factor binding sites were determined by sequence motif scores represented by the position-specific weight matrices (PWM). The PWM for CTCF, MAX, SIX5, USF1, BCL11A, EGR1, NANOG, RAD21, RFX5, SRF, USF2, GABP, NRSF, YY1, CJUN, JUND , OCT4, TCF12 were downloaded from JASPAR (Mathelier et al. 2014) and factorbook (Wang et al. 2012). The PWM matching function from Bioconductor package "Biostrings" (Pages et al.) is used to scan the entire genome to identify candidate sites for TFBS. After the selection, most TFs have 200k to 600k candidate sites with a median of 335,600 candidate sites for H1-hESC cell line. Detailed candidate sites information is shown in the Table S2-1 and S2-2 below. Among all the candidate sites, those located inside a ChIP-seq peak are considered as positives. The rest are considered as negatives.

Candidate TF sites located within CpG islands are not considered by default. This is due to the wide-spread hypomethylation known to occur at the CpG islands, which could potentially biase the methylation model estimation. Since CpG islands only account for less than 1% of the genome, such exclusion shouldn’t affect the result much. Methylphet software provides an option for users to choose to include candidate sites within CpG islands.

Table S2-1. Number of candidate TFBS in H1-hESC

TF	# of Candidate sites	min.score[*1]	# of Positive[*2]	# of Negative[*2]	# of total peaks[*2]	# of peaks without motif[*2]	Motif ID[*3]
BCL11A	725,974	95%	1,615	724,359	2,664	1,049	
CEBPB	297,822	97.5%	5,340	292,482	44,858	39,518	MA0466.1
CJUN	1,160,903	90%	2,445	1,158,458	7,383	4,938	
CTCF	389,712	85%	35,000	354,712	58,399	23,399	MA0139.1
GABP	505,709	90%	1,331	504,378	17,228	15,897	
MAFK	280,465	95%	4,591	275,874	27,552	22,961	MA0496.1
MAX	685,346	90%	12,874	672,472	33,747	20,873	MA0058.2
NANOG	363,495	96%	1,295	362,200	15,176	13,881	
NRSF	219,928	80%	6,014	213,914	16,818	10,804	
OCT4	165,299	90%	854	164,445	3,662	2,808	
RAD21	389,712	85%	51,211	338,501	85,393	34,182	
RFX5	28,573	93%	489	28,084	5,653	5,164	MA0510.1
SIX5	564,533	80%	1,961	562,572	3,106	1,145	
SOX2	288,139	99.5%	1,385	286,754	5,684	4,299	MA0143.3
SRF	331,388	90%	2,327	329,061	4,684	2,357	MA0083.2
TCF12	2,235,295	93%	16,785	2,218,510	26,715	9,930	MA0521.1
USF1	532,348	90%	23,607	508,741	30,097	6,490	MA0093.2
USF2	532,348	90%	5,664	526,684	12,675	7,011	MA0526.1
YY1	99,052	90%	3,150	95,902	35,587	32,437	MA0095.2

*1 min.score is the parameter used in R package Biostring for motif scan.
*2 # of positive is the number of candidate sites overlapping with peak, which is equivalent to the number of peaks containing motif; # of Negative is the number of candidate sites without overlapping with peak; # of peaks are from ChIP-seq.
*3 Motif ID are JASPAR IDs. Some TFs don't have JASPAR ID; we find the PWM matrices of these TFs in Factorbook, Supplemental Table S2: http://genome.cshlp.org/content/suppl/2012/08/22/22.9.1798.DC1/TableS2.pdf

Table S2-2. Motif logos based on PWMs for all the TFs are produced[*]

**BCL11A**<br>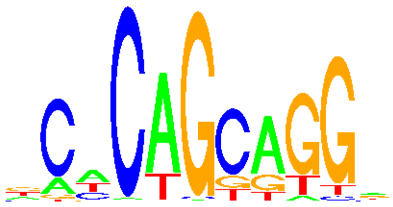	**CEBPB**<br>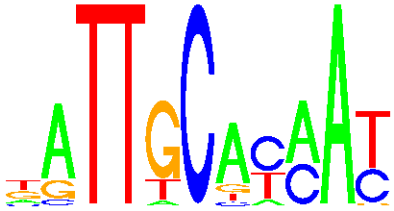
**CJUN**<br>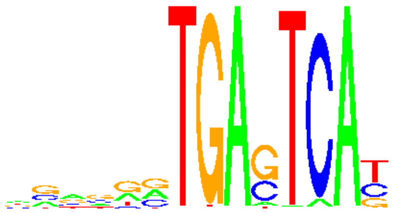	**CTCF**<br>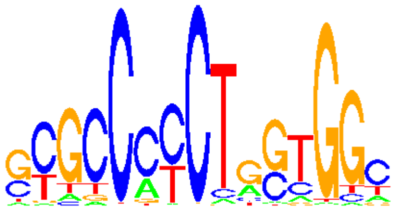
**GABP**<br>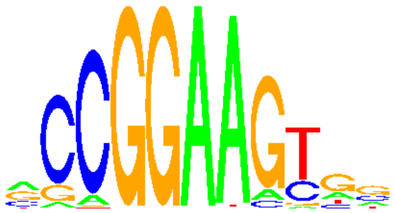	**MAFK**<br>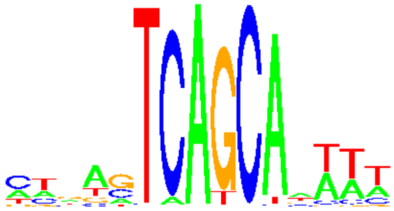

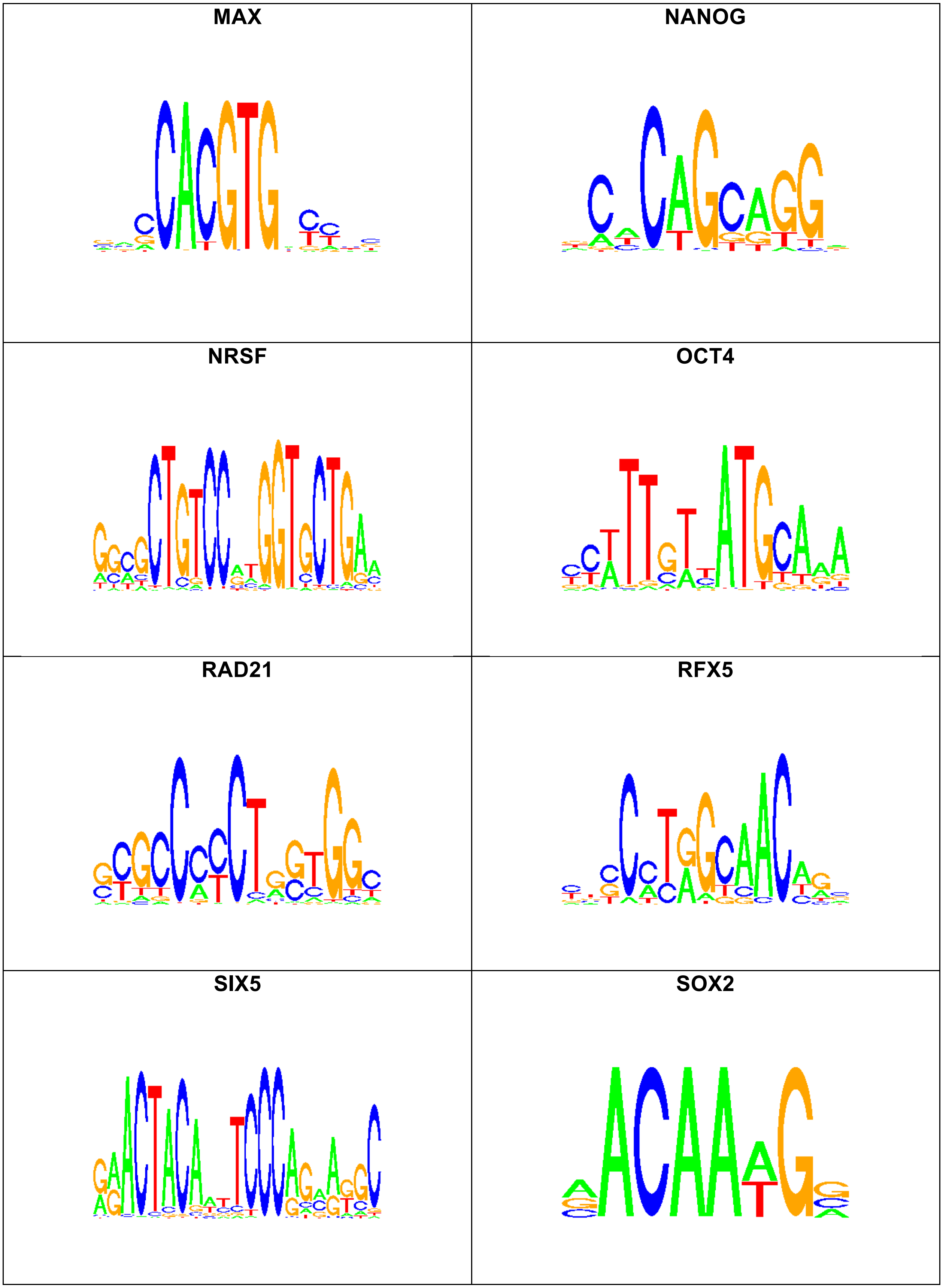
MAX
NANOG
NRSF
OCT4
RAD21
RFX5
SIX5
SOX2

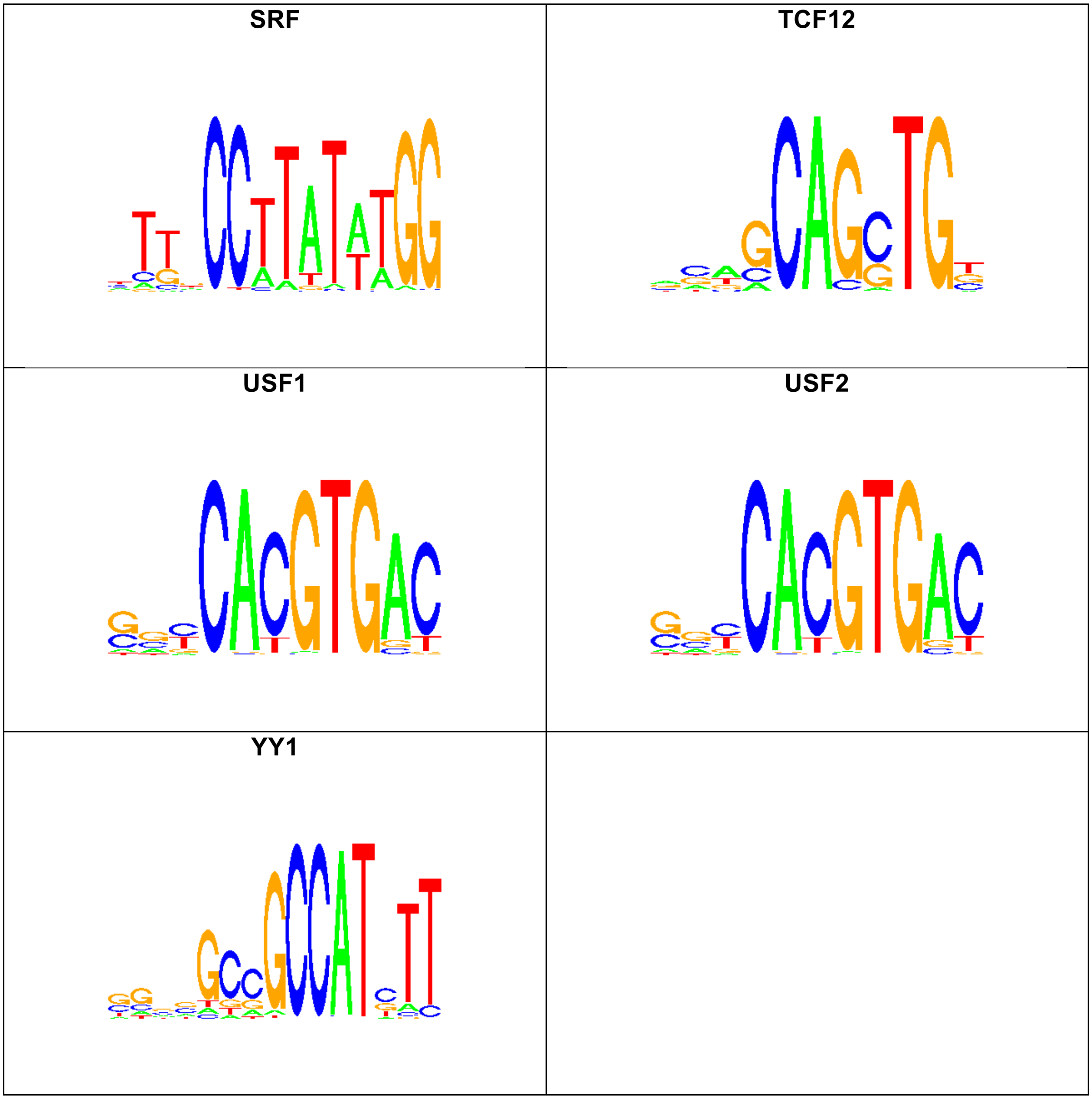

*   The height of motif logo is scaled to its information content.

3. Methylation profile

3.1 Methylation profile (CG, 5hmC, CH) in H1-hESC.

Figure S1 shows the profiles of CpG methylation (CG), CpG hydroxyl-methylation (5hmC) and CpH methylation (CH) from H1-hESC for a number of TFs.

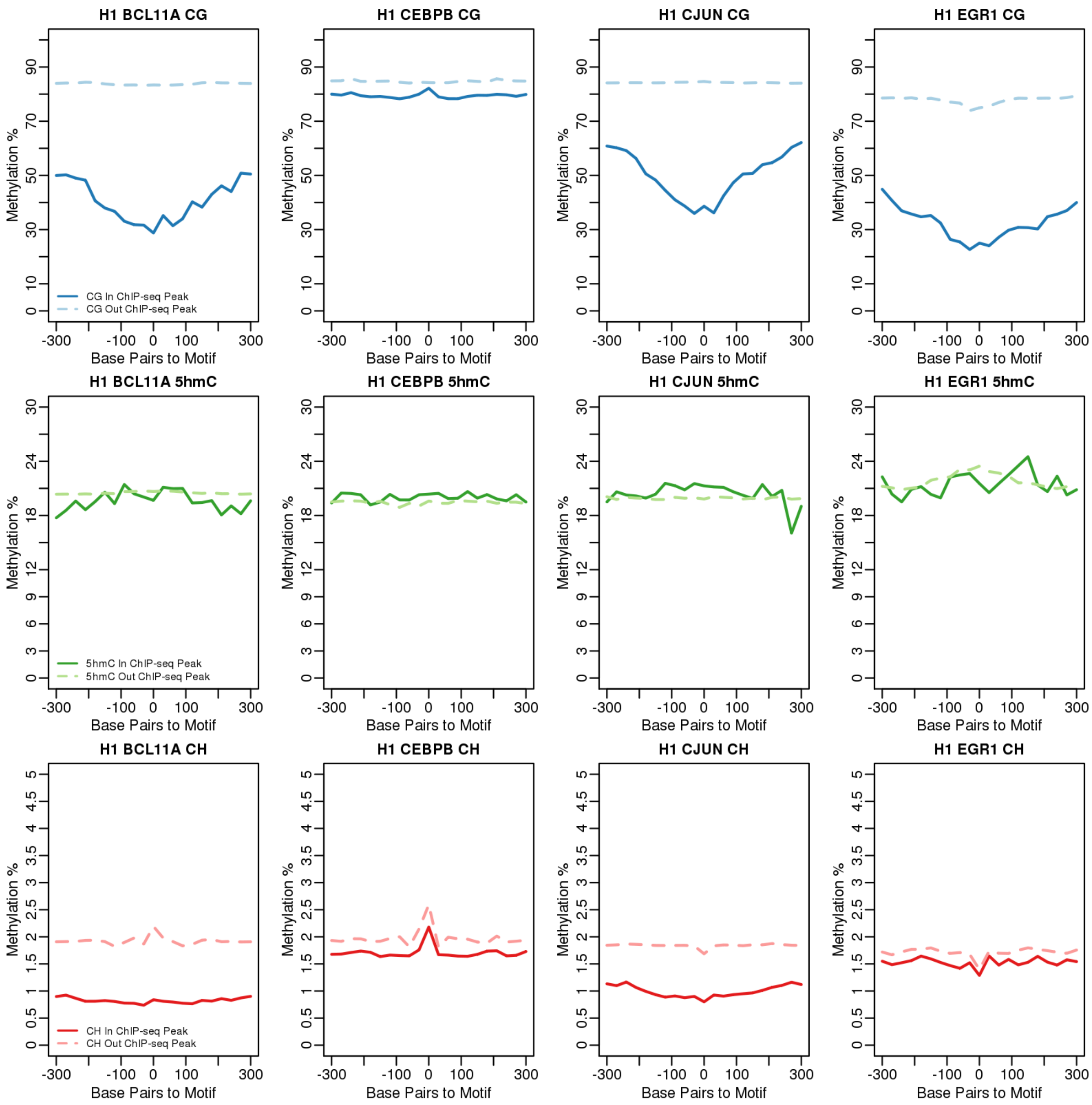

Figure S1-1. Methylation profile (CG, 5hmC, CH) of BCL11A, CEBPB, CJUN and EGR1 in H1-hESC.

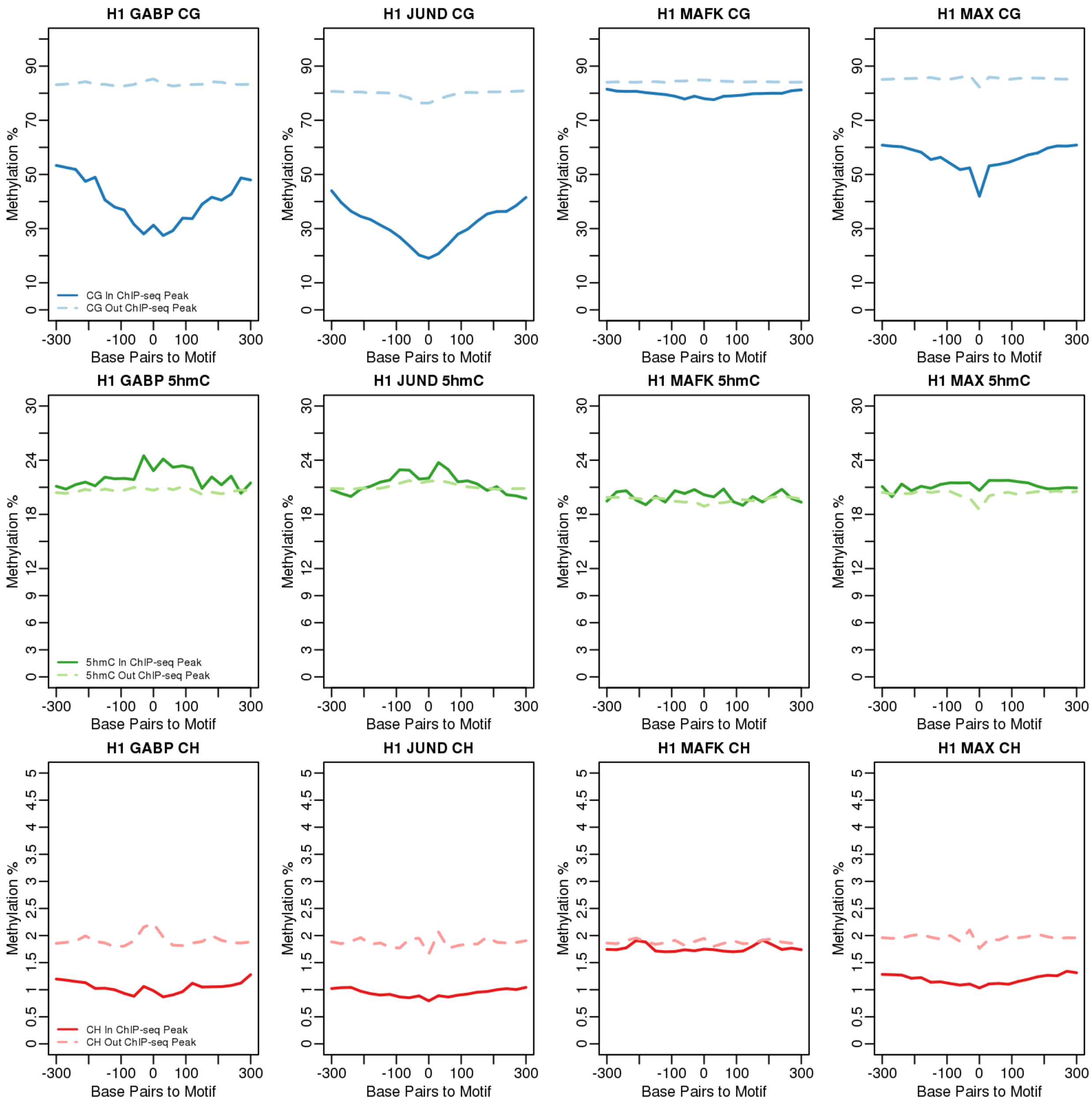

Figure S1-2. Methylation profile (CG, 5hmC, CH) of GABP, JUND, MAFK and MAX in H1-hESC.

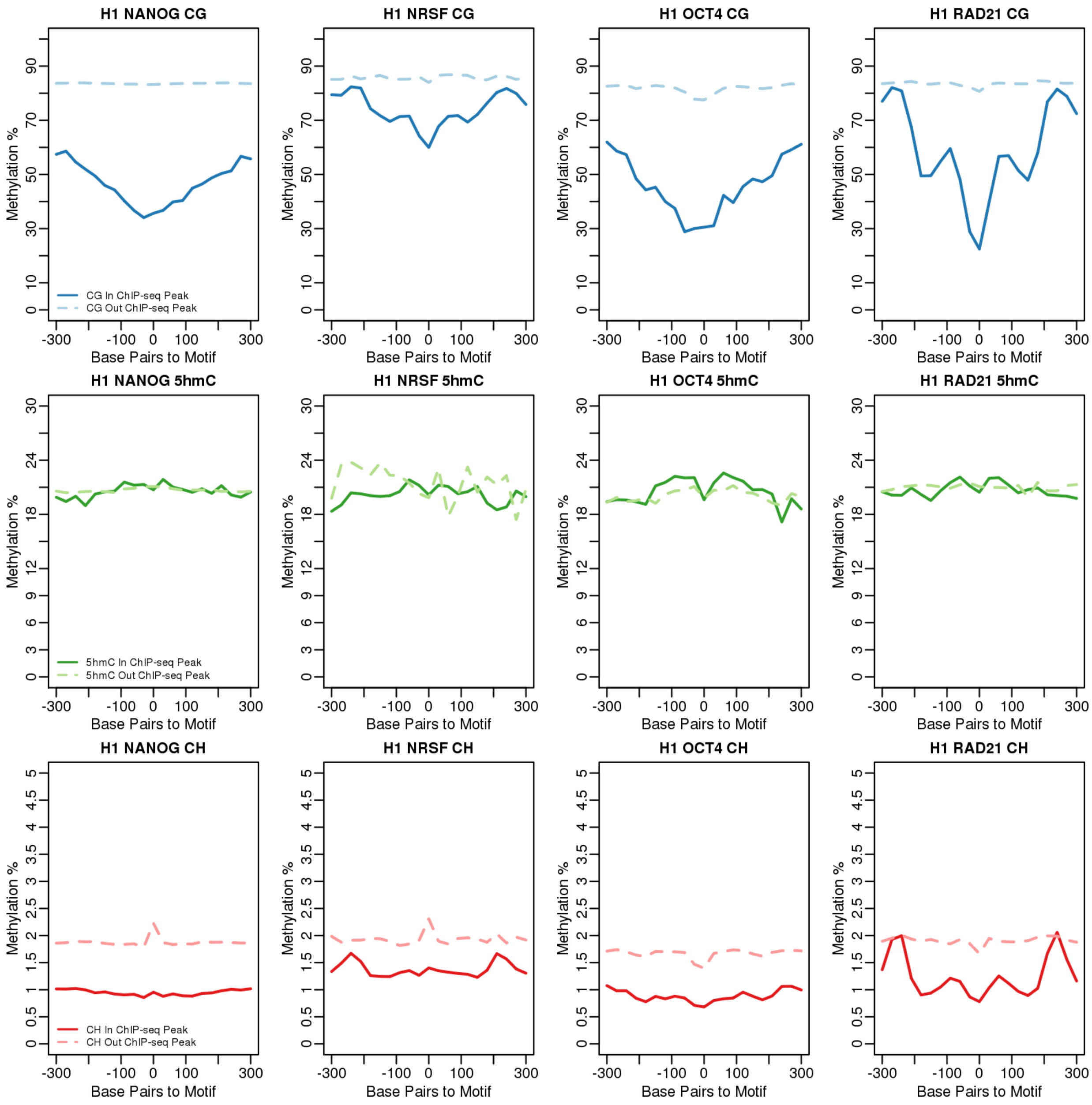

Figure S1-3. Methylation profile (CG, 5hmC, CH) of NANOG, NRSF, OCT4 and RAD21 in H1-hESC.

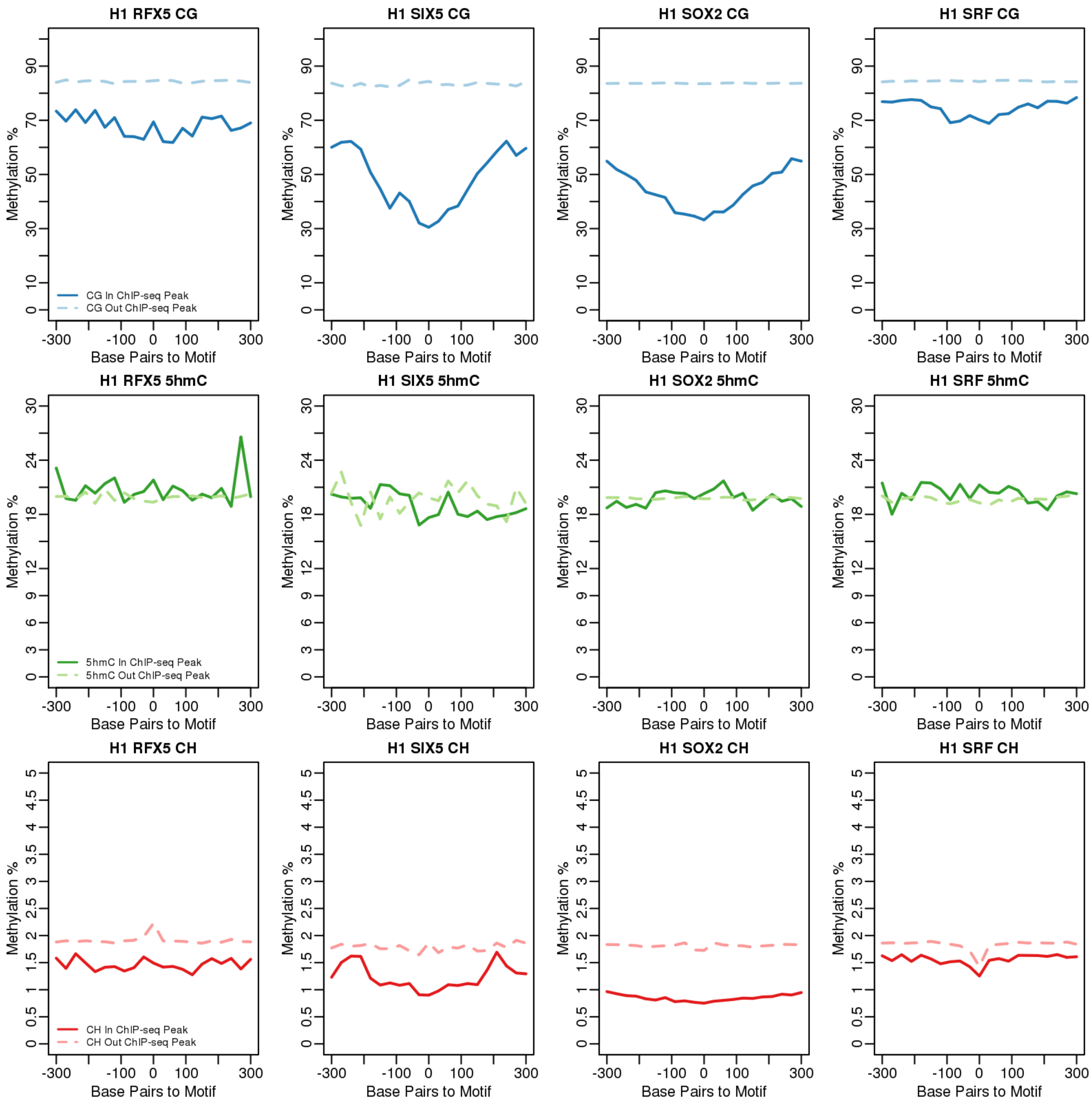

Figure S1-4. Methylation profile (CG, 5hmC, CH) of RFX5, SIX5, SOX2 and SRF in H1-hESC.

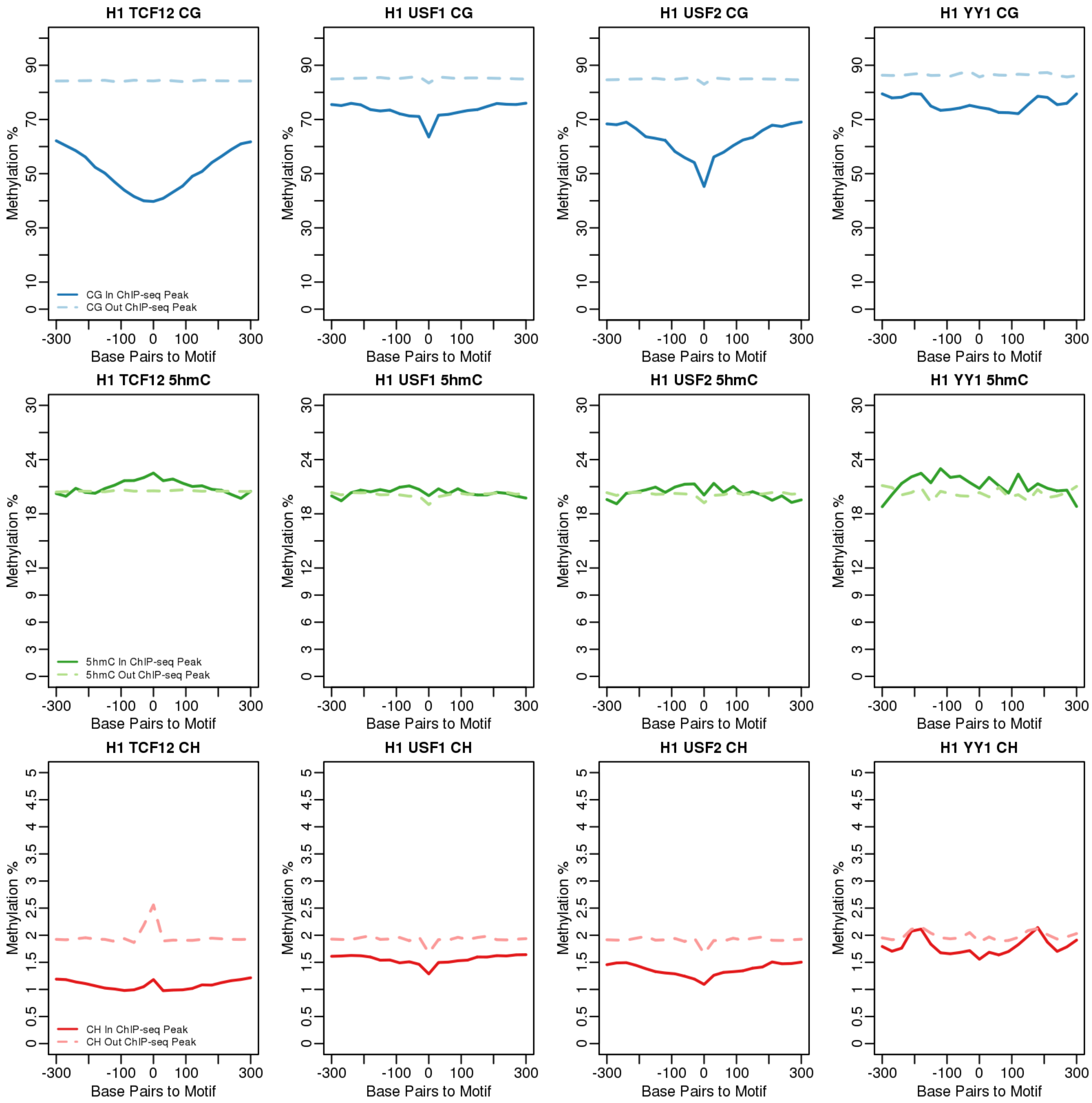

Figure S1-5. Methylation profile (CG, 5hmC, CH) of TCF12, USF1, USF2 and YY1 in H1-hESC.

3.2 Methylation profile (CG, CH) in IMR90.

Figure S2 shows the profiles of CG and CH from IMR90 for a number of TFs. Because the TAB-seq data are not available, the 5hmC profiles are not shown.

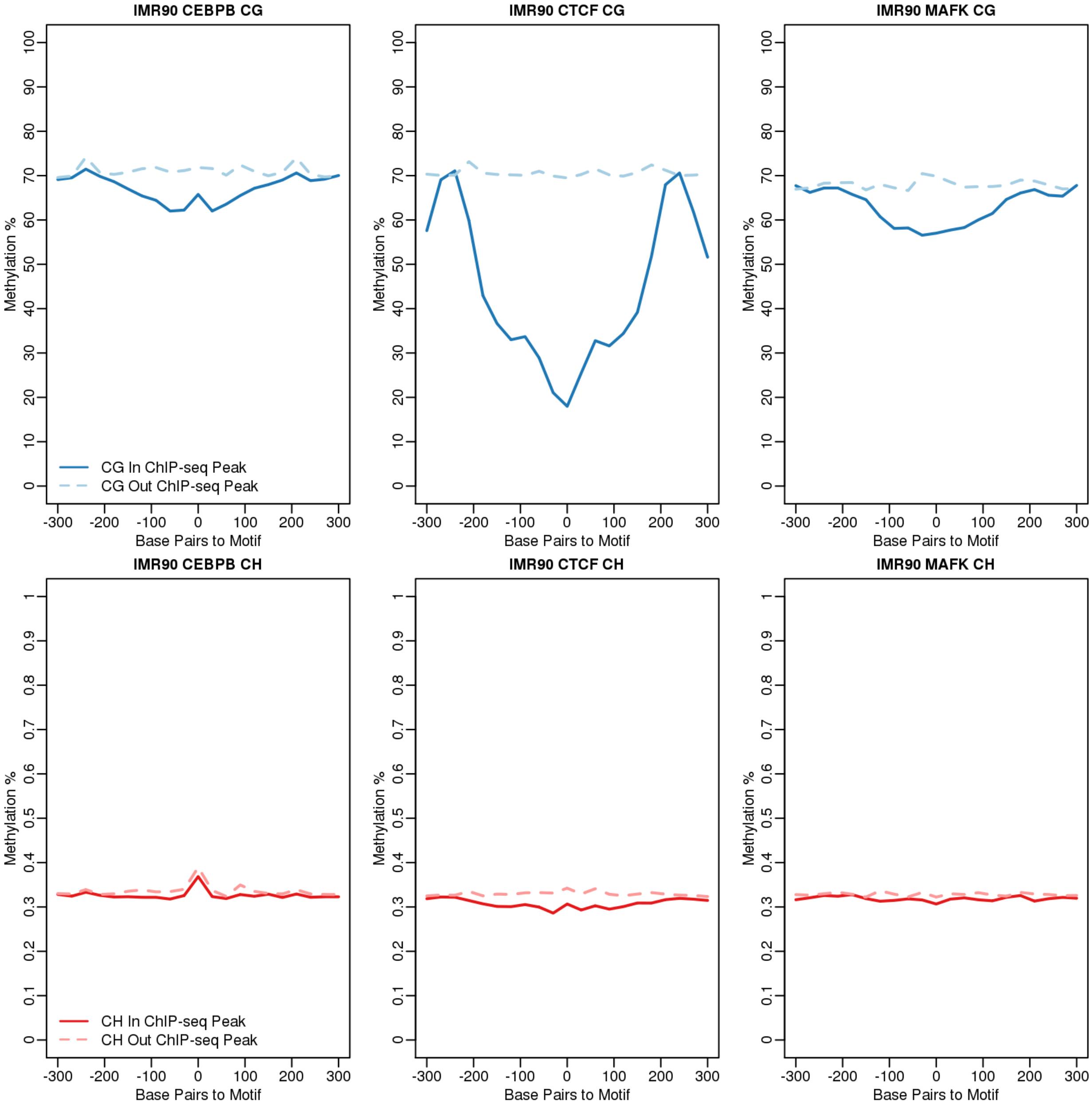

Figure S2-1. Methylation profile (CG, CH) of CEBPB, CTCF and MAFK in IMR90.

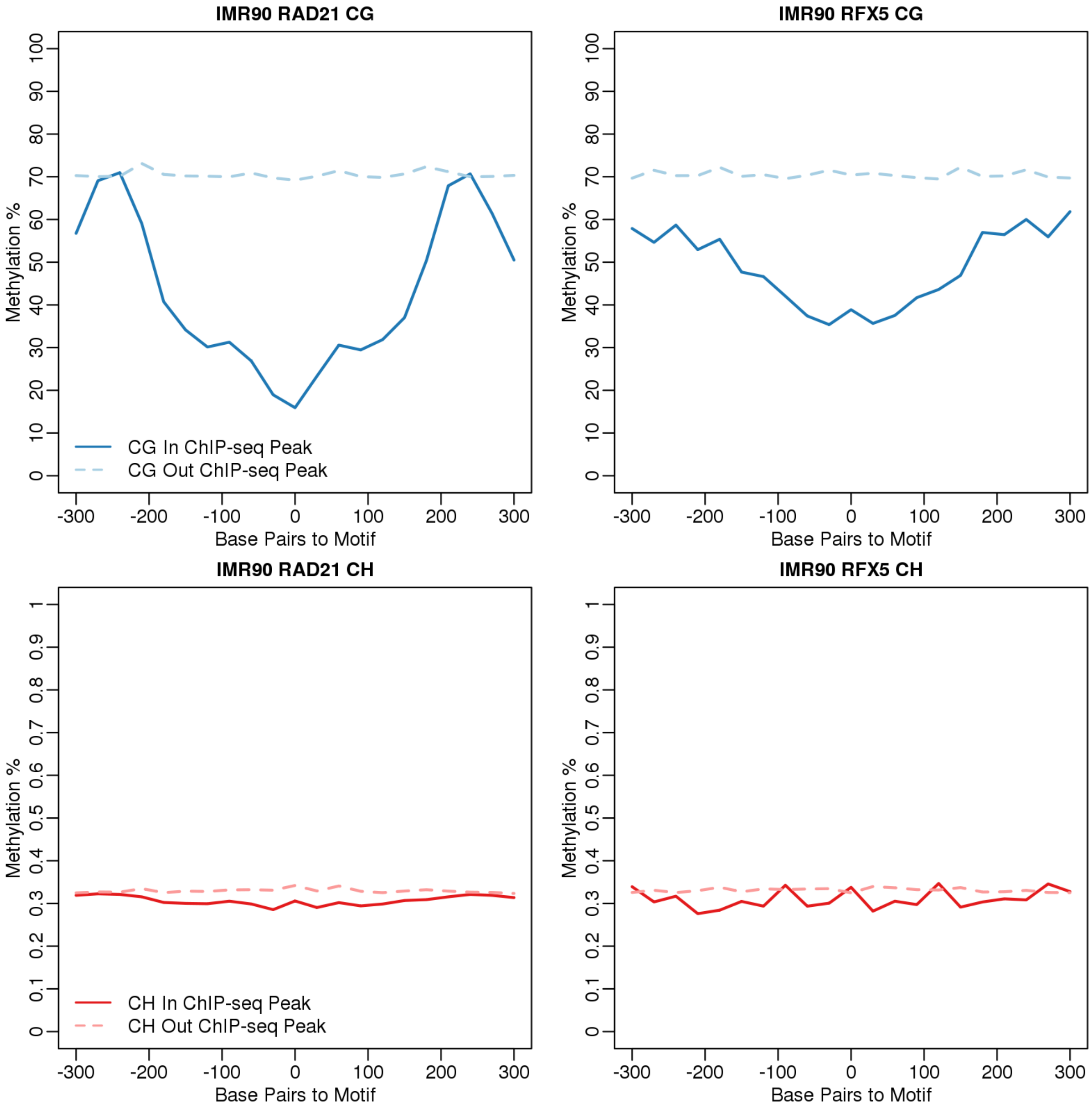

Figure S2-2. Methylation profile (CG, CH) of RAD21 and RFX5 in IMR90.

3.3 Methylation profile (CG, 5hmC and CH) in mESC.

Figure S3 shows the profiles of CG, 5hmC and CH methylation from mESC for a number of TFs.

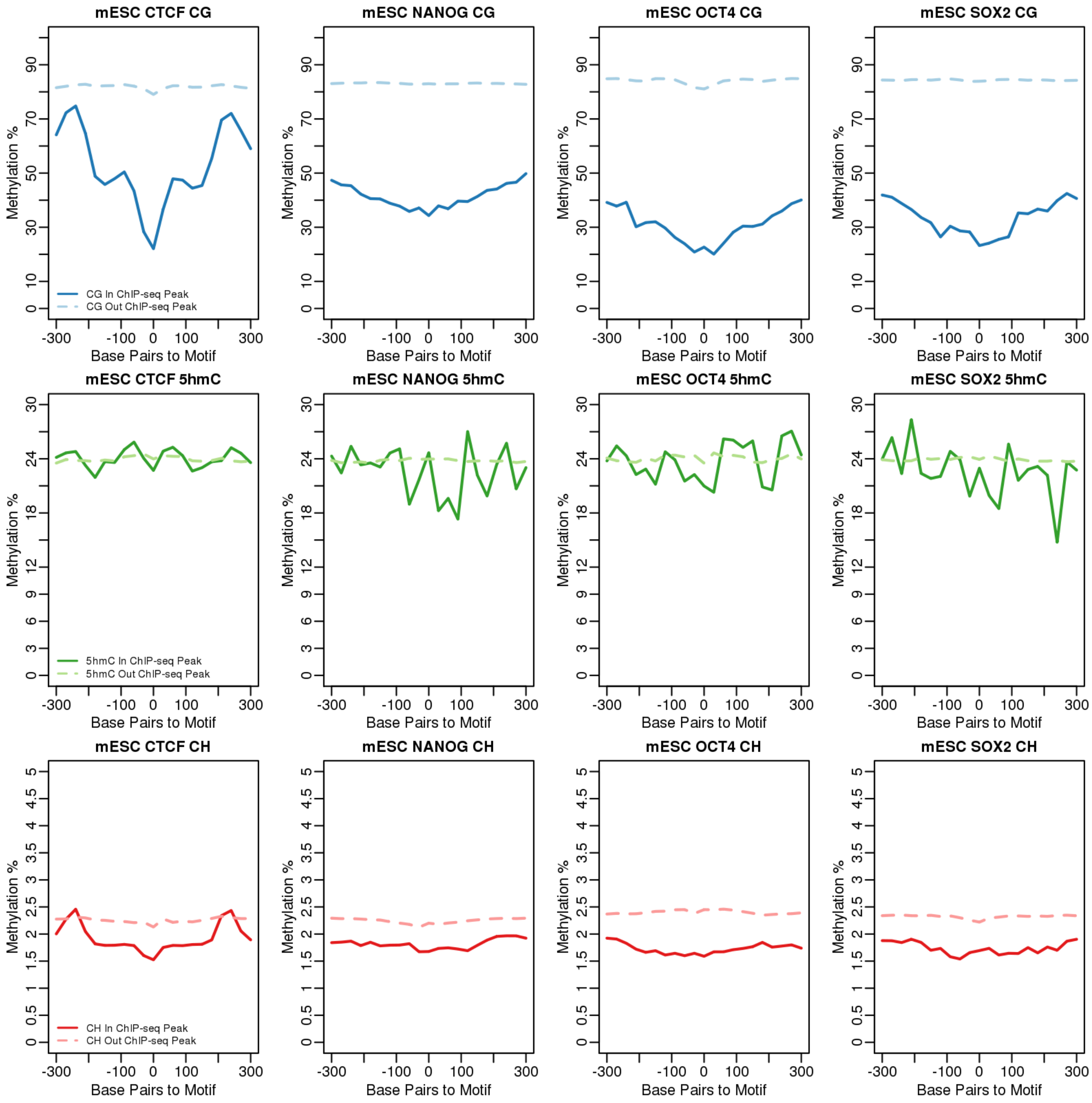

Figure S3. Methylation profile (CG, 5hmC and CH) of CTCF, NANOG, OCT4 and SOX2 in mESC.

4. Methylphet Model

Beta-binomial model is used to characterize methylation (including 5mC, 5hmC and CH methylation) patterns at a genomic region. For a certain candidate site, 21 window with 30bps each centered at the center of the motif. The methylation reads and total reads in these 21 windows are used to capture methylation patterns for TF binding sites as well as background methylation level. Inside each window, if there is at least one CG dinucleotide that is covered by at least one read (either methylated or not), we recorded the total number of methylated and un-methylated reads. Assume there are n candidate sites. In the jth window ($j = 1,2,\ldots,21$) of the ith candidate site ($i = 1,2,\ldots n$), we use x_{ij} and y_{ij} to denote the number of methylated and unmethylated reads and let $n_{ij} = x_{ij} + y_{ij}$. The counts, given underlying "true" methylation levels are assumed to follow binomial distribution

$$x_{ij} | n_{ij}, p_j \sim Binom(n_{ij}, p_j), i = 1,2,\ldots n; j = 1,2,\ldots 21.$$

The methylation levels p_j's are assumed to follow a beta distributions, but with different parameters at TF binding sites and background. For candidate sites that are bound by TFs, it is shown (Figure 2) that methylation levels dip toward the motif site from both directions. For candidate sites that are not bound by TFs, the methylation levels from all 21 windows are assumed to be identical and similar to those from the genomic background (close to fully-methylated). Therefore we assume that each p_j follows a different Beta distribution. Define indicator z_i to denote binding ($z_i = 1$) or not ($z_i = 0$) for candidate site i, we have:

$$\begin{cases} p_j | z_i = 1 \sim Beta(\alpha_j, \beta_j) \\ p_j | z_i = 0 \sim Beta(\alpha', \beta') \end{cases}$$

To build the predictive model, parameters of $\alpha_j, \beta_j, \alpha', \beta'$ will be estimated using the training data. Here, we used method of moments to obtain the estimate for these parameters. It is convenient to consider an alternative representation of beta binomial compound model. We define:

$$\begin{cases} \mu_j = \dfrac{\alpha_j}{\alpha_j + \beta_j} \\ M_j = \alpha_j + \beta_j \end{cases}$$

Then the beta binomial model can be reparameterized as:

$$\begin{cases} x_{ij} | n_{ij}, p_j \sim Binom(n_{ij}, p_j), i = 1,2,\ldots n; j = 1,2,\ldots 21 \\ p_j | z_i = 1 \sim Beta(\mu_j, M_j) \\ p_j | z_i = 0 \sim Beta(\mu', M') \end{cases}$$

The expectation and variance for $\frac{x_{ij}}{n_{ij}}$ are:

$$E\left(\frac{x_{ij}}{n_{ij}}\right) = E\left(E\left(\frac{x_{ij}}{n_{ij}} \mid p_j\right)\right) = \mu_j$$

$$var\left(\frac{x_{ij}}{n_{ij}}\right) = E\left(var\left(\frac{x_{ij}}{n_{ij}} \mid p_j\right)\right) + var\left(E\left(\frac{x_{ij}}{n_{ij}} \mid p_j\right)\right)$$

$$= E\left(\frac{p_j(1-p_j)}{n_{ij}} \mid \mu_j, M_j\right) + var(p_j \mid \mu_j, M_j)$$

$$= \frac{\mu_j - \left(\mu_j^2 + \frac{\mu_j(1-\mu_j)}{M_j+1}\right)}{n_{ij}} + \frac{\mu_j(1-\mu_j)}{M_j+1}$$

$$= \frac{\mu_j(1-\mu_j)}{n_{ij}} + \frac{n_{ij}-1}{n_{ij}}\frac{\mu_j(1-\mu_j)}{M_j+1}$$

$$= \frac{\mu_j(1-\mu_j)}{n_{ij}}\left(1 + \frac{n_{ij}-1}{M_j+1}\right)$$

The sample mean and sample variance for $\frac{x_{ij}}{n_{ij}}$ are:

$$\overline{\frac{x_{ij}}{n_{ij}}} = \frac{\sum_{i=1}^{n}\frac{x_{ij}}{n_{ij}}}{n}$$

$$S^2 = \frac{\sum_{i=1}^{n}\left(\frac{x_{ij}}{n_{ij}} - \widehat{\mu_j}\right)^2}{n-1}$$

By method of moments, we have:

$$\begin{cases} \widehat{\mu_j} = \frac{\sum_{i=1}^{n}\frac{x_{ij}}{n_{ij}}}{n} \\ \frac{\widehat{\mu_j}(1-\widehat{\mu_j})}{n_{ij}}\left(1 + \frac{n_{ij}-1}{\widehat{M_j}+1}\right) = \frac{\sum_{i=1}^{n}\left(\frac{x_{ij}}{n_{ij}} - \widehat{\mu_j}\right)^2}{n-1} \end{cases}$$

Hence, we can obtain the point estimate for μ_j, M_j and further to obtain the point estimate for $\alpha_j, \beta_j$by

$$\begin{cases} \widehat{\alpha_j} = \widehat{\mu_j} * \widehat{M_j} \\ \widehat{\beta_j} = \left(1 - \widehat{\mu_j}\right) * \widehat{M_j} \end{cases}$$

5. Methylphet Performance

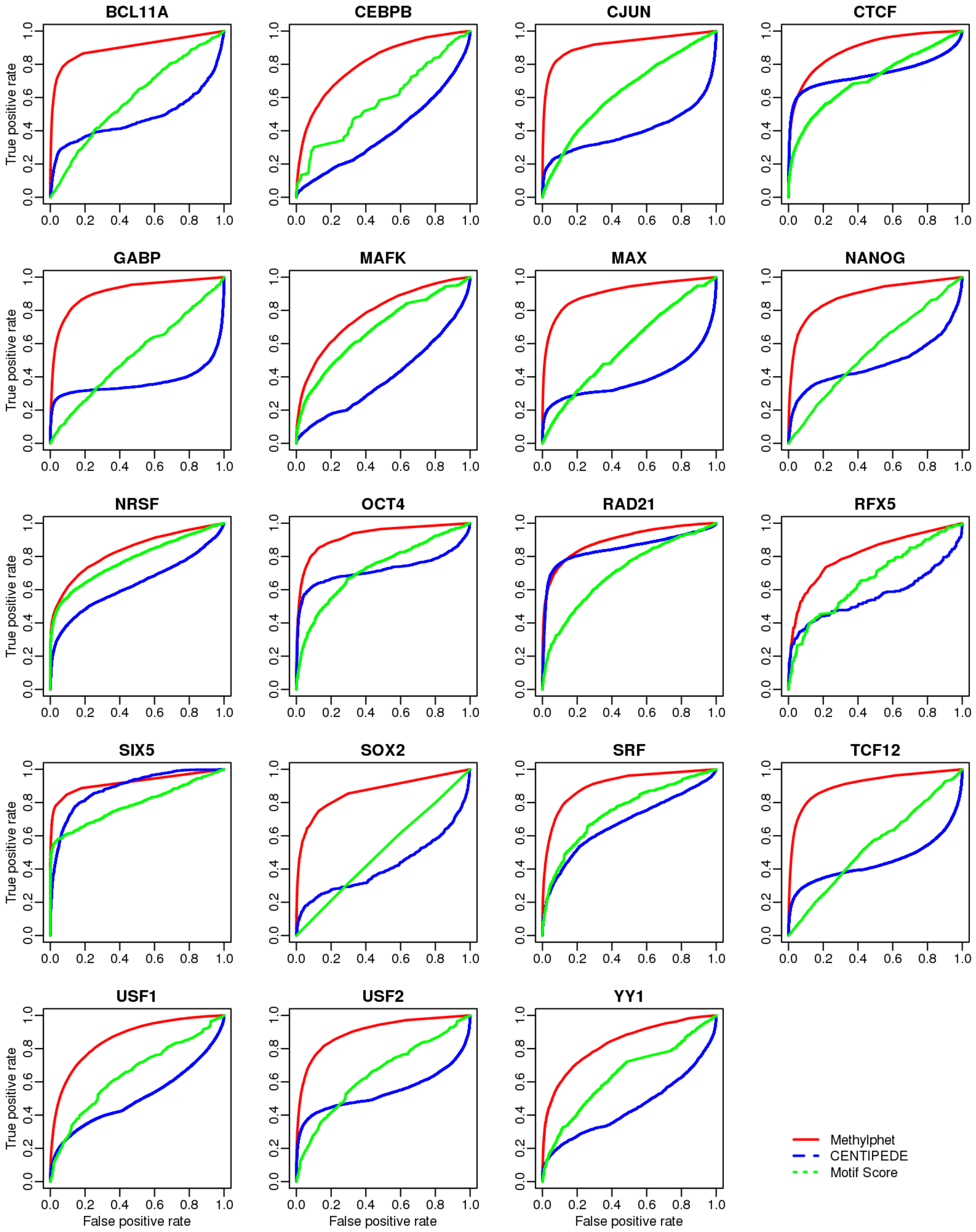

Figure S4. ROC curves of 19 TFs in H1-hESC; Cross-validation within cell line.

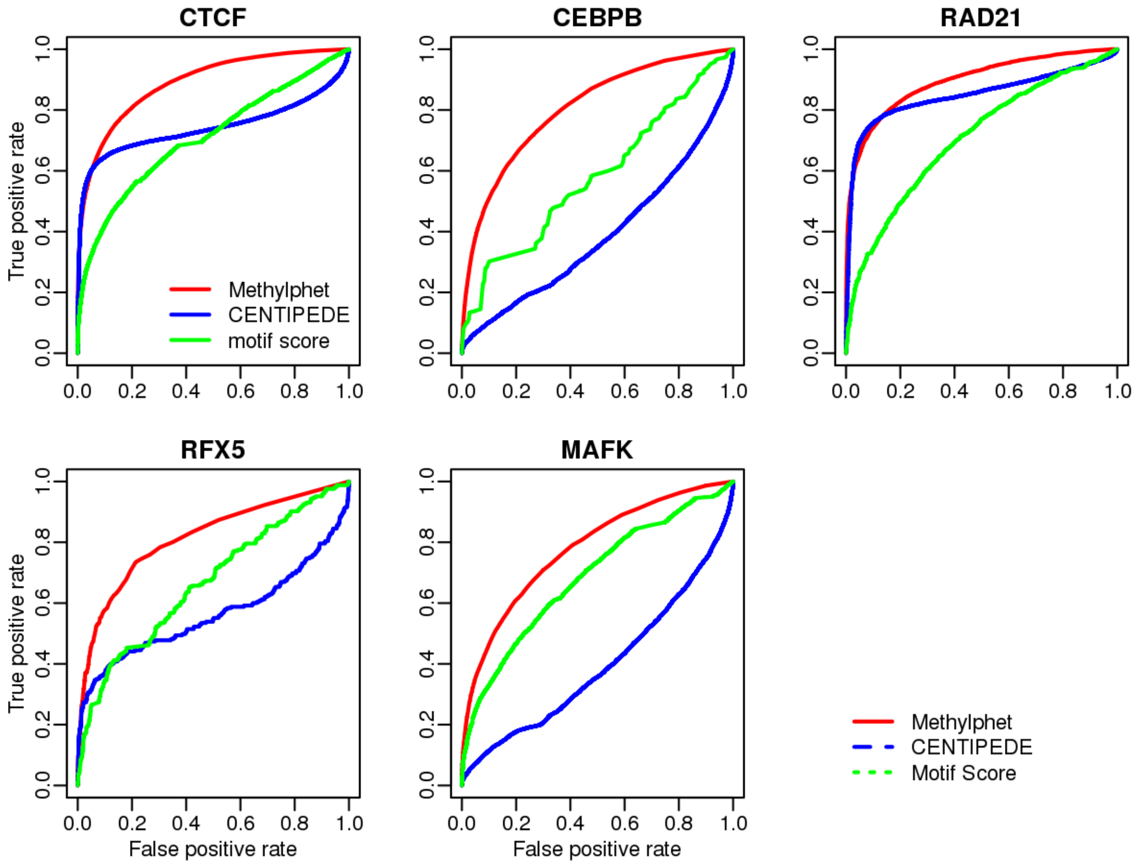

Figure S5. ROC curves of 5 TFs in IMR90; Cross-validation within cell line.

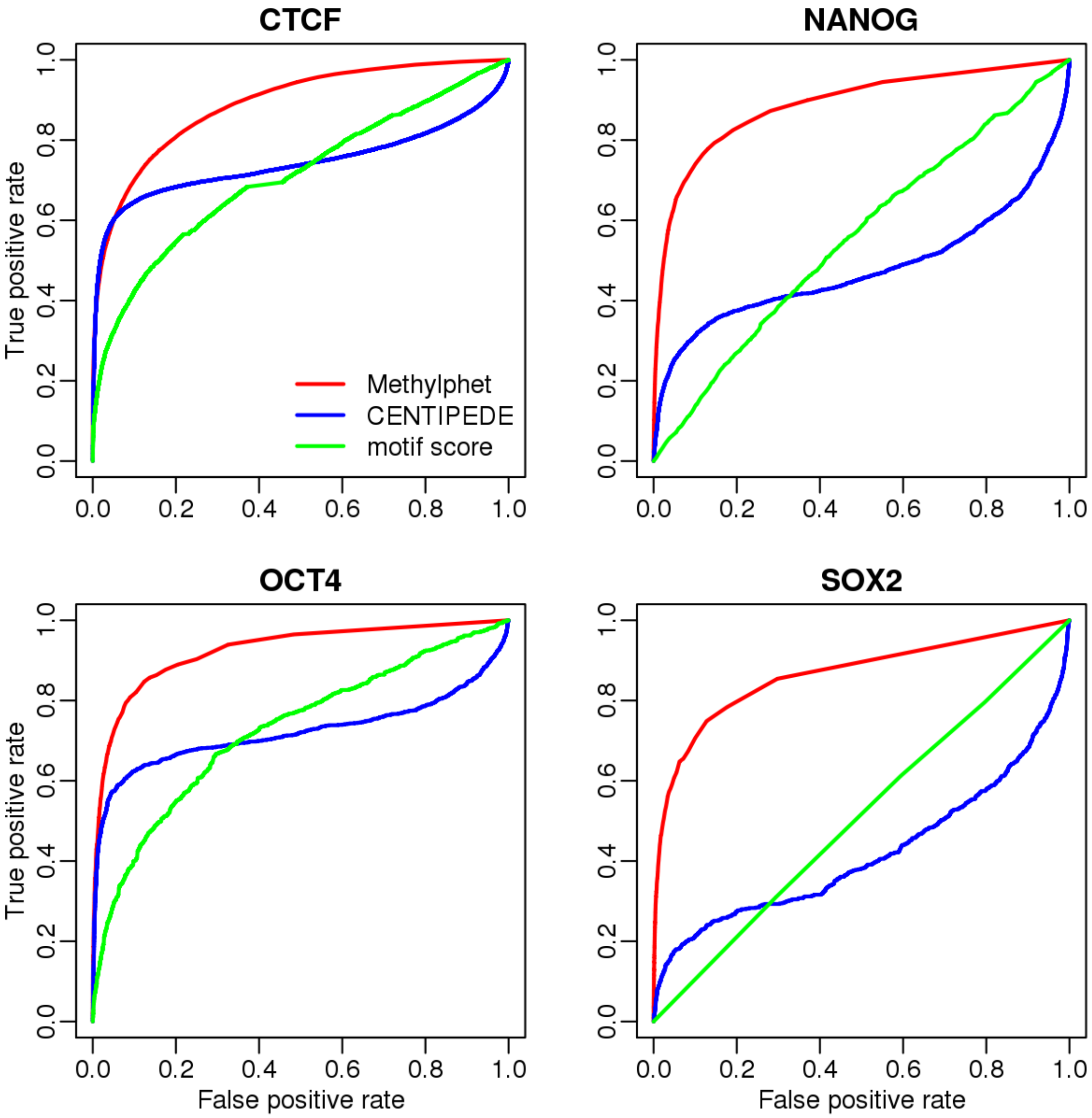

Figure S6. ROC curves of 4 TFs in mESC; Cross-validation within cell line.

6. Features in Methylphet

6.1 Features Description

The features used in Methylphet's second step are listed in Table S3.

Table S3. Features used in Methylphet

Feature name	Description
CG	Methylation score on CpG sites
CH	Methylation score on CH sites (not CpG)
5hmC	Hydroxymethylation score
Motif	Motif scan score (see section "Candidate TFBS selection")
TSS.dist	Distance between the center of candidate TFBS and the nearest TSS
Motif.dist	Distance between the center of candidate TFBS and the nearest center of other candidate TFBS
PhastCon	Average conservation score of all bases on candidate TFBS. PhastCon score was obtained from UCSC phastcon44ways for hg18, and phastCons30ways for mm9.
Repeat	Boolean, if the candidate TFBS is overlapping with a repetitive region. Repeat regions were as defined by UCSC repeat Masker
Intron	Boolean, if the candidate TFBS is overlapping with an Intron
Exon	Boolean, if the candidate TFBS is overlapping with an Exon
TSS	Boolean, if the candidate TFBS is overlapping with an TSS
TES	Boolean, if the candidate TFBS is overlapping with an TES
CpG	Boolean, if the candidate TFBS is overlapping with an CpG Island

6.2 Evaluation of Feature Importance

The importance of each feature is measured by the Gini Importance. This measure is the total decrease in node impurities from splitting on the variable, averaged over all trees. Therefore, the more decrease in node impurity the feature causes, the more important this feature is. In this binary classification case, the node impurity is measured by the Gini index (or Gini coefficient, Gini ratio). It is calculated as:

$$I_G(p) = 2p_0p_1$$

Where p_0 and $p_1$are the fraction of items labeled as 0 and 1 respectively.

In Figure S7-1 ~S7-19, we examined the features used in Methylphet. Within each figure, panel (A) shows the pair-wise correlation between features. In general there're no strong correlation observed

among features. (B) Gini importance of different features, sorted in decreasing order. (C) The ROC curve of Methylphet with/without 5hmc and CH. The predicting performance will be generally improved by including 5hmc and CH score. (D) Model convergence check (See below).

6.3 Model convergence and tree number

In the random forest model, Out-of-bag error (OOB error, details in the manual of R package '*randomForest*') was used to evaluate model convergence as the number of trees grows. In our model, we examine the model convergence as the number of trees used in training grows from 0 to 200. In general, the model becomes stable when number of trees reaches <100. (Figure S7-1 ~ S7-19, panel D)

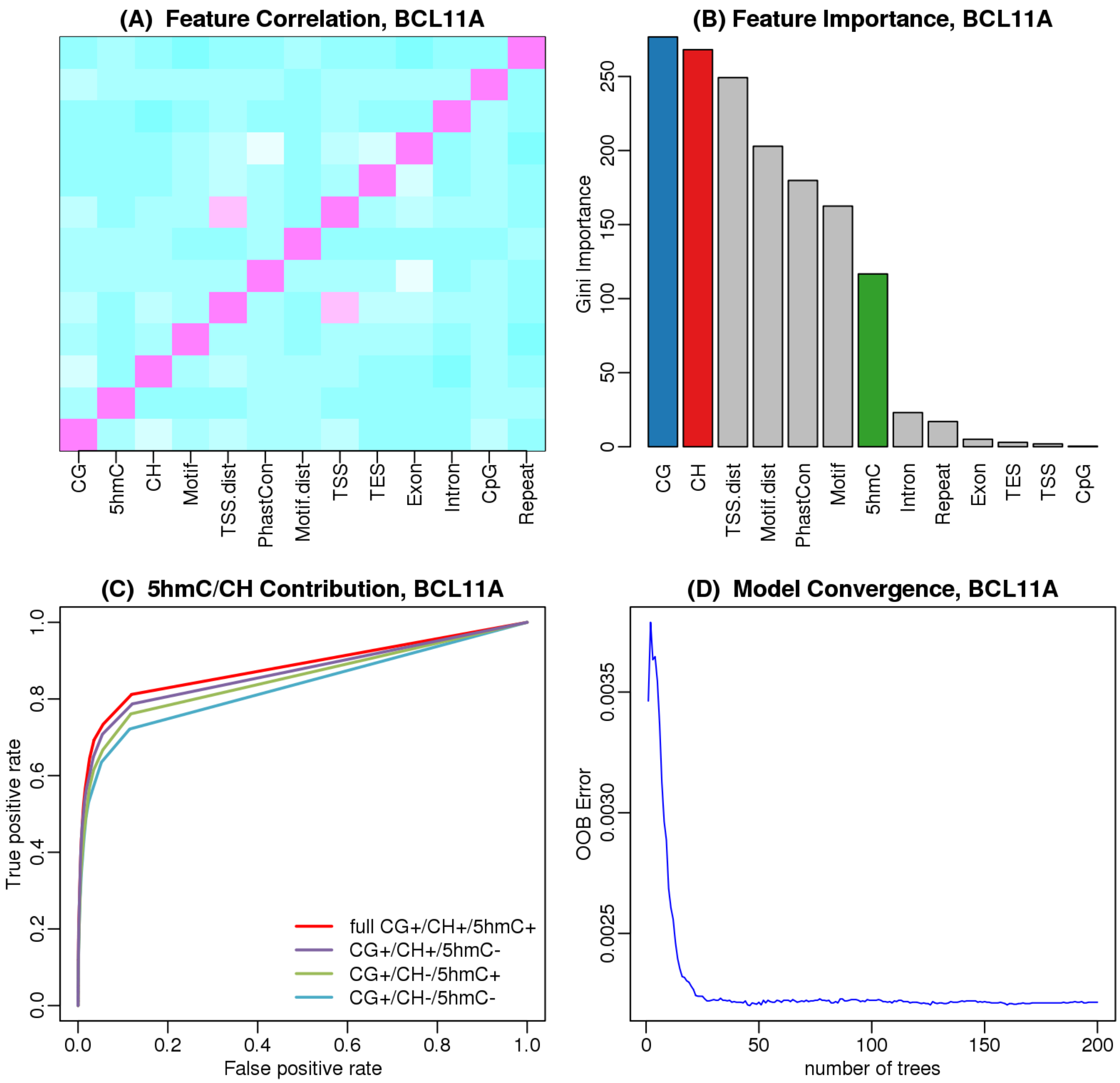

Figure S7-1. Characteristics of features; BCL11A in H1-hESC.

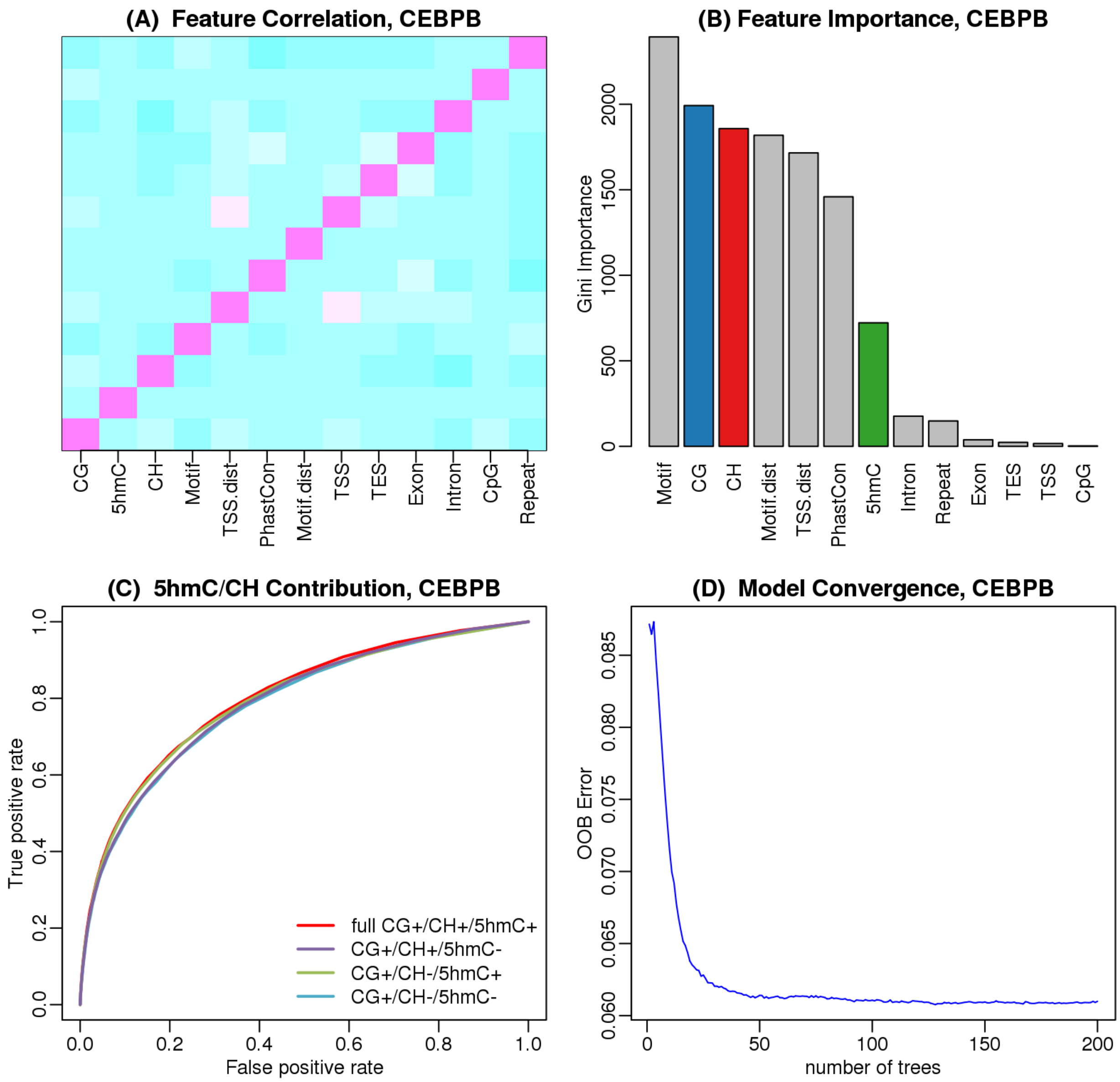

Figure S7-2. Characteristics of features; CEBPB in H1-hESC.

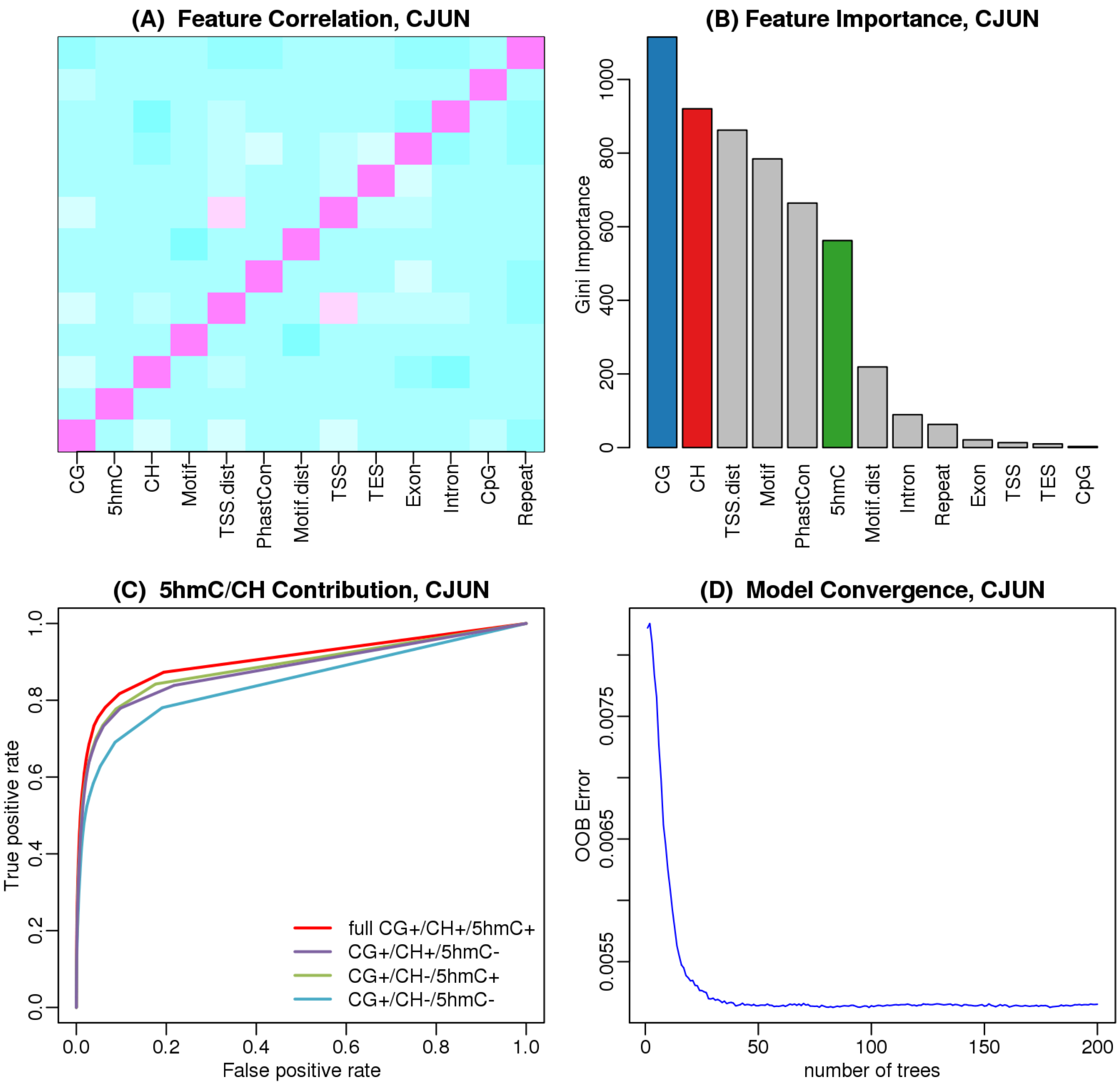

Figure S7-3. Characteristics of features; CJUN in H1-hESC.

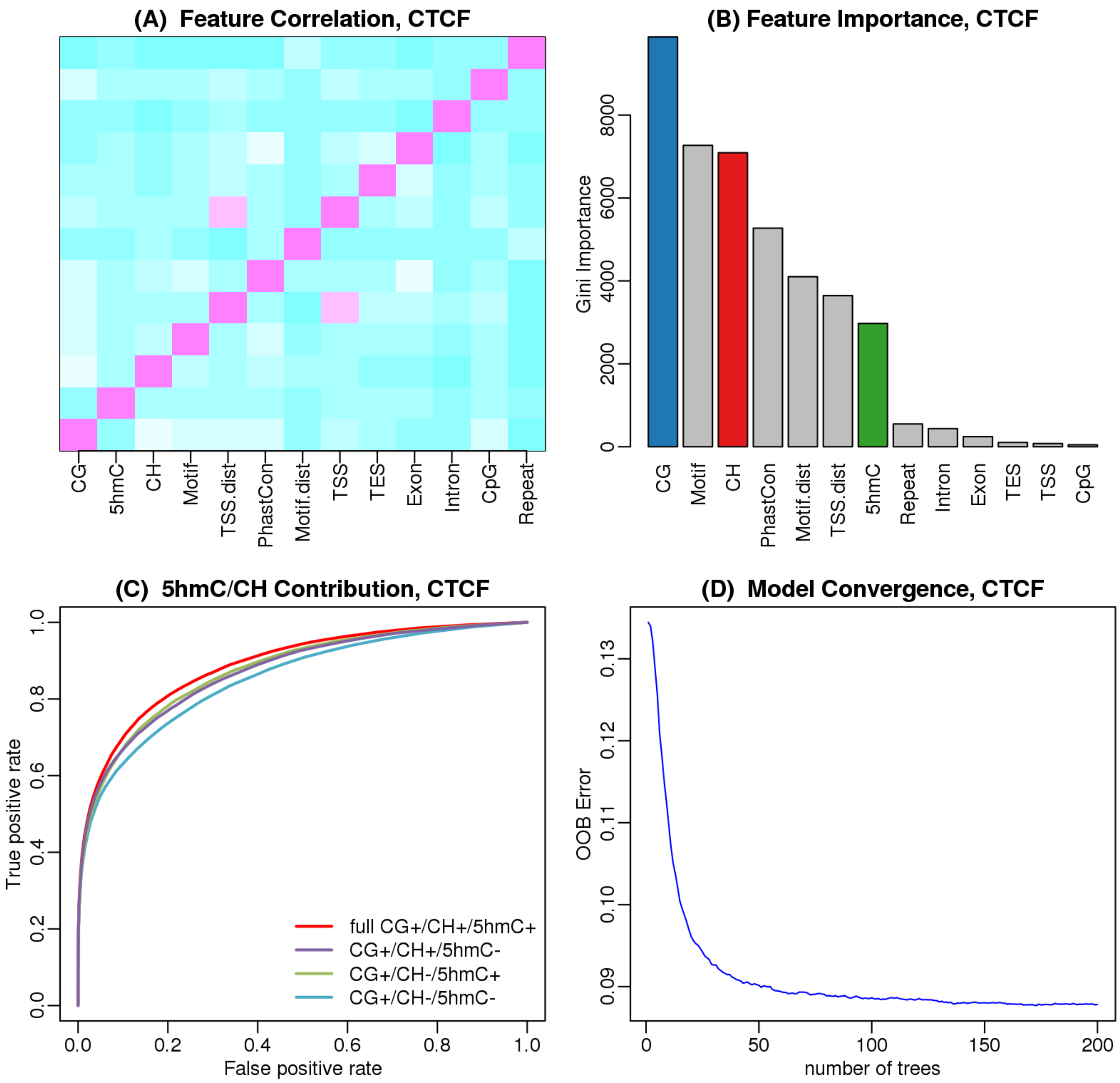

Figure S7-4. Characteristics of features; CTCF in H1-hESC.

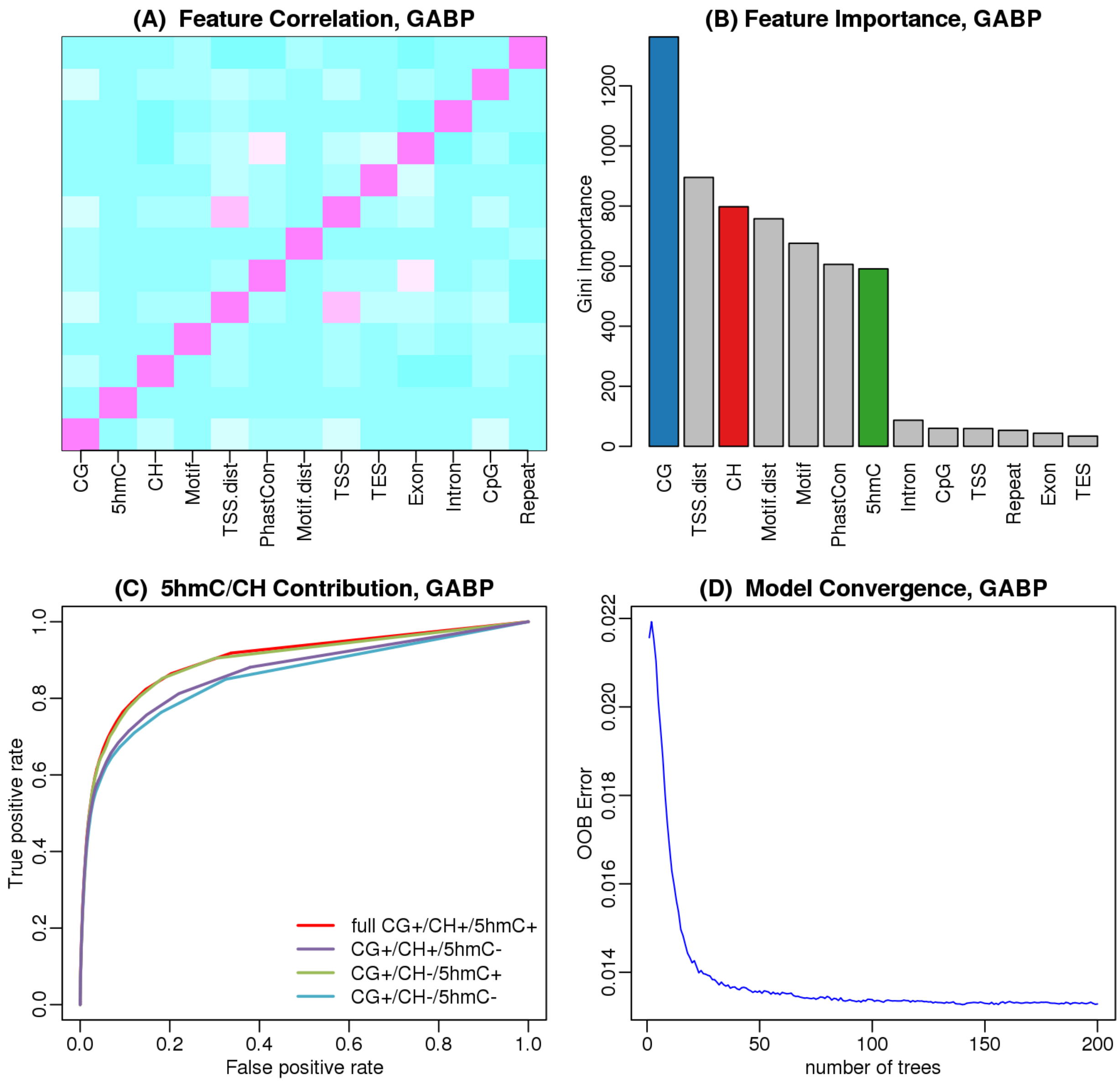

Figure S7-5. Characteristics of features; GABP in H1-hESC.

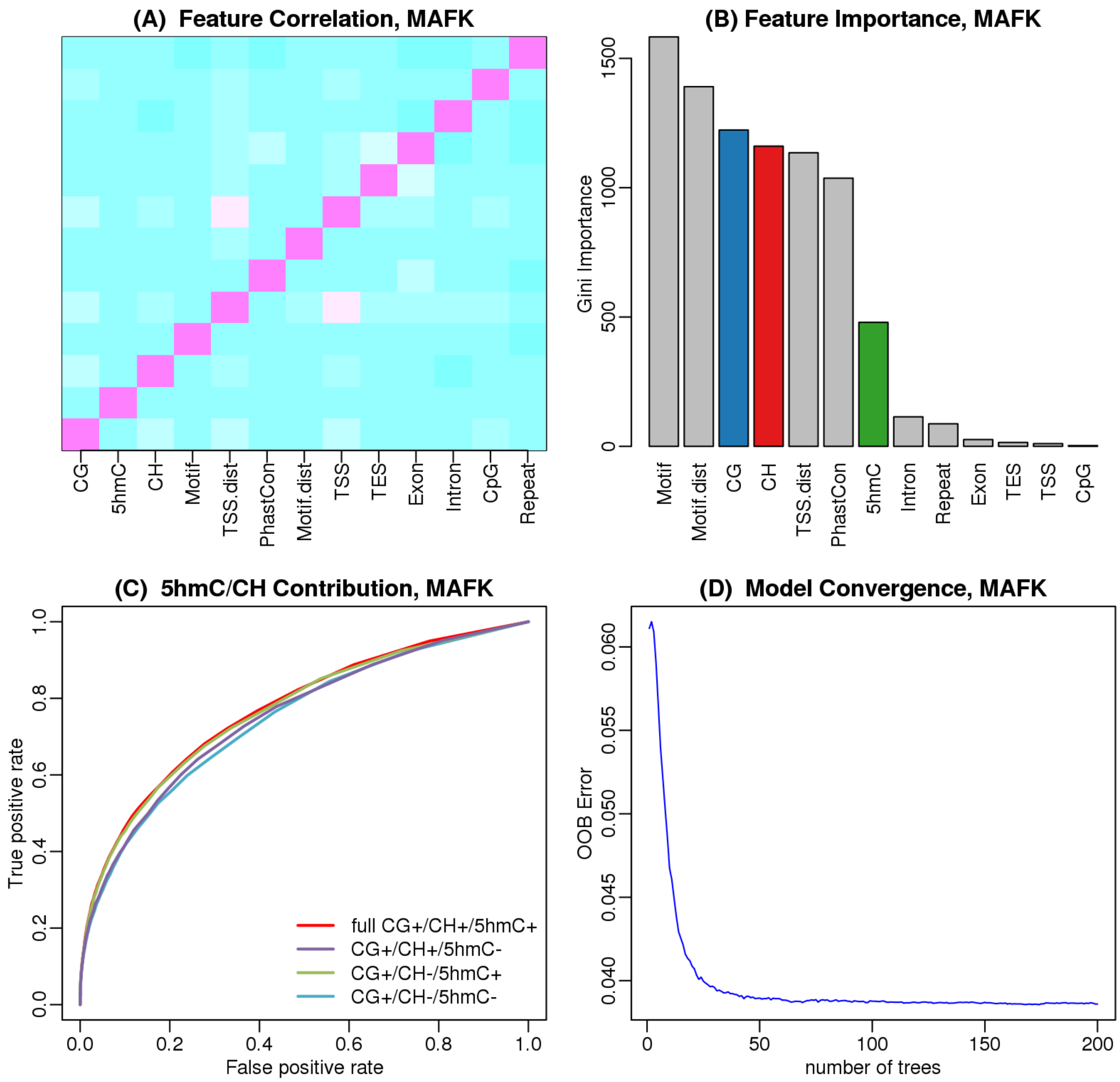

Figure S7-6. Characteristics of features; MAFK in H1-hESC.

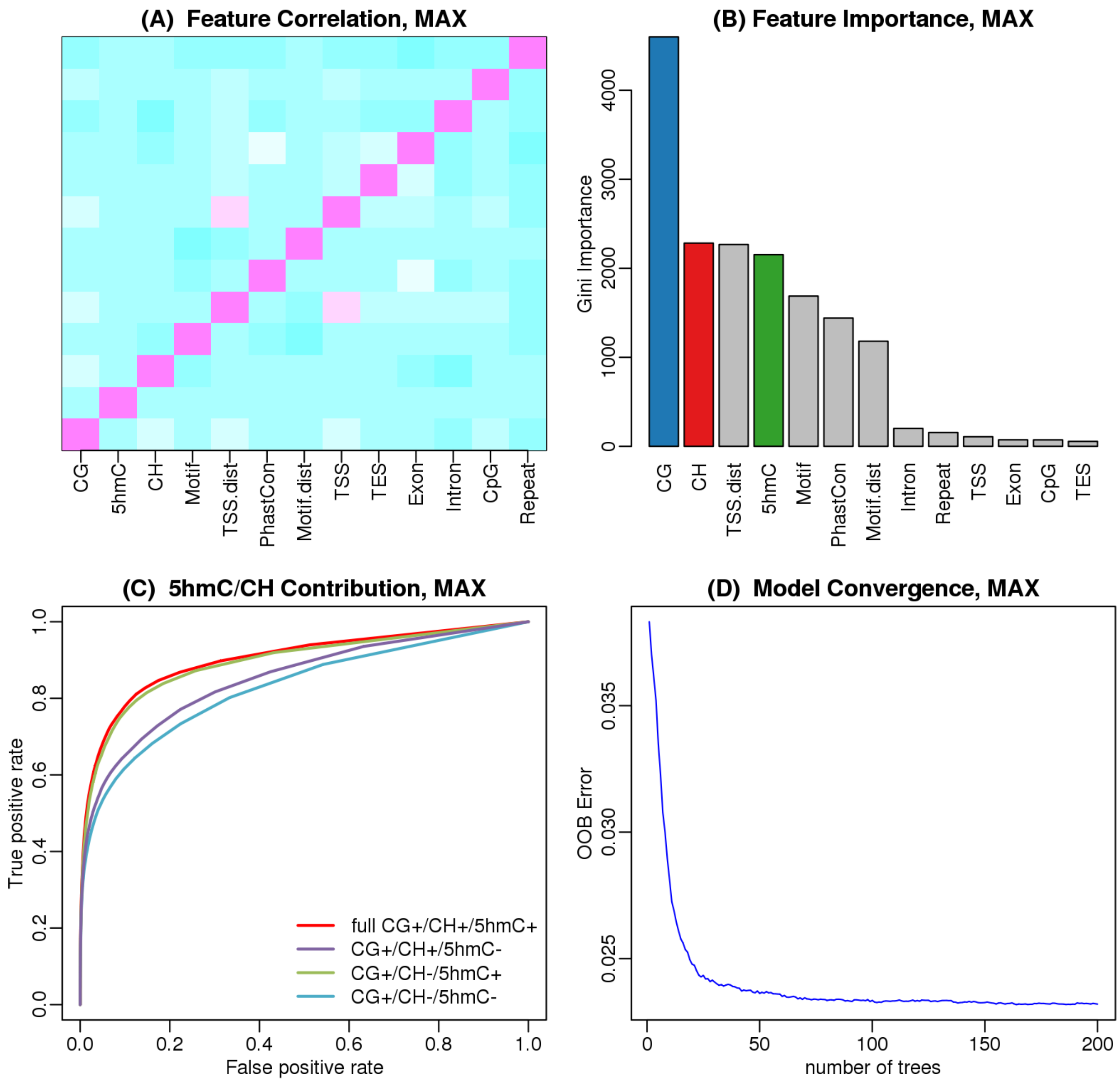

Figure S7-7. Characteristics of features; MAX in H1-hESC.

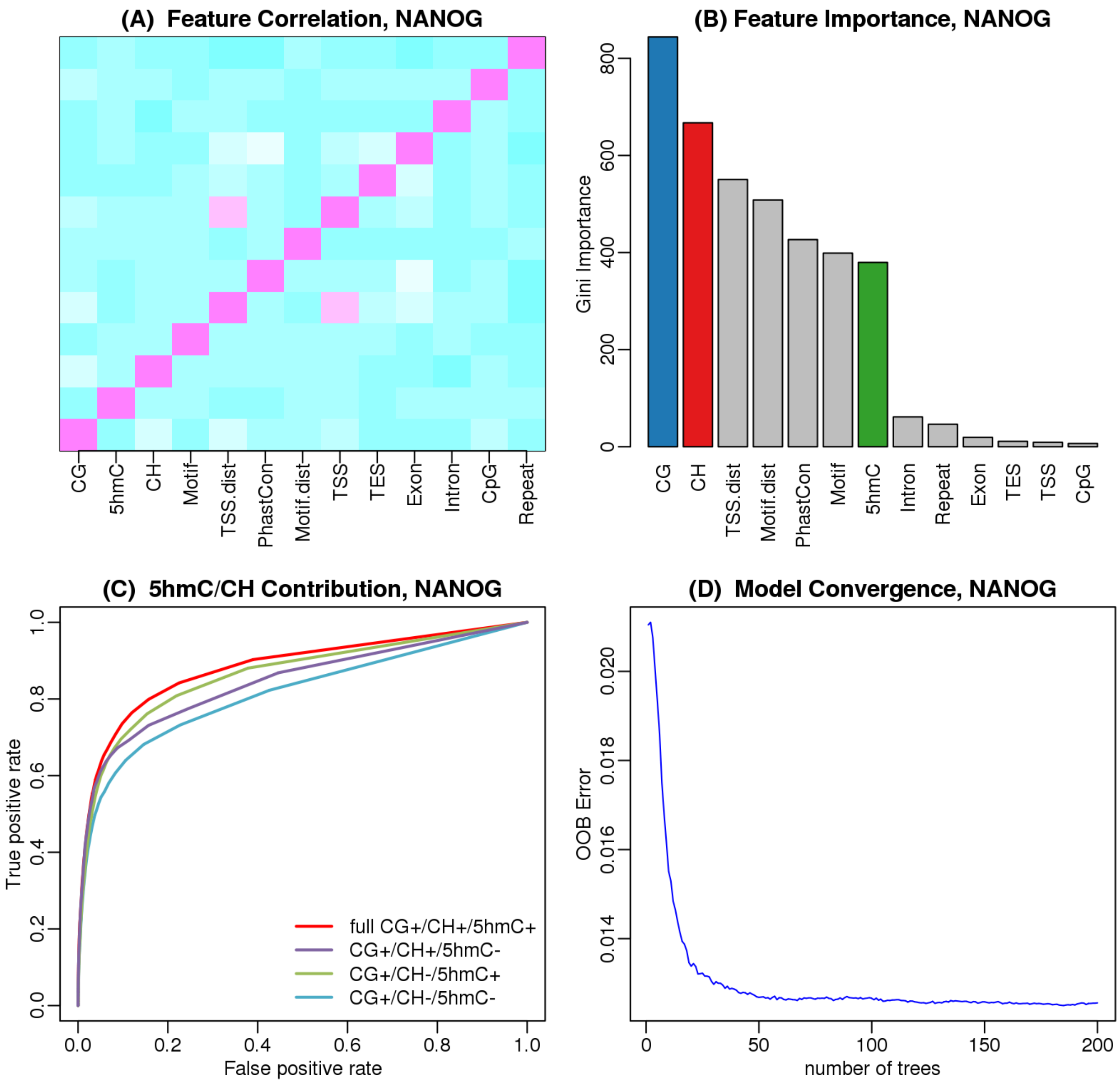

Figure S7-8. Characteristics of features; NANOG in H1-hESC.

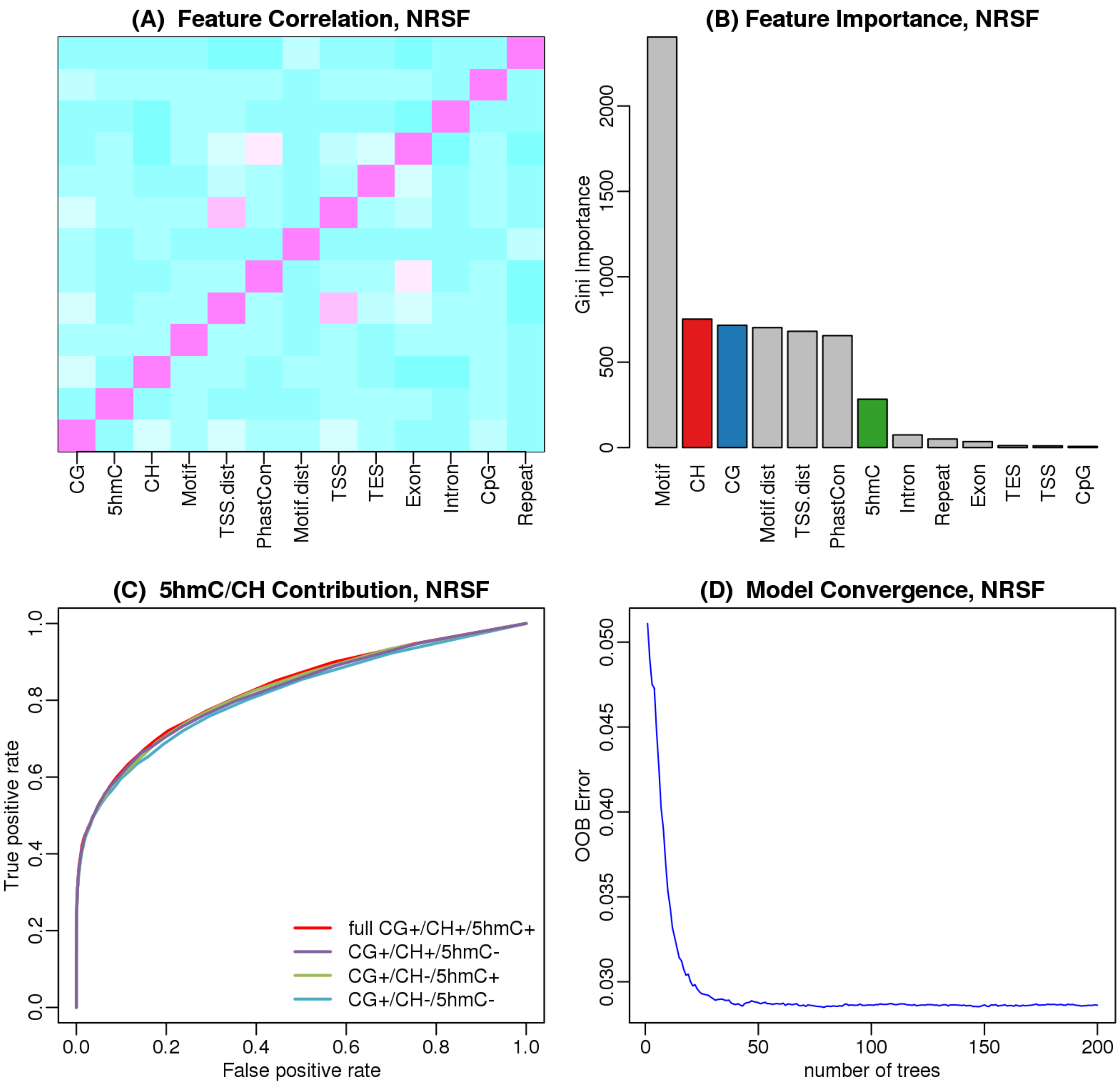

Figure S7-9. Characteristics of features; NRSF in H1-hESC.

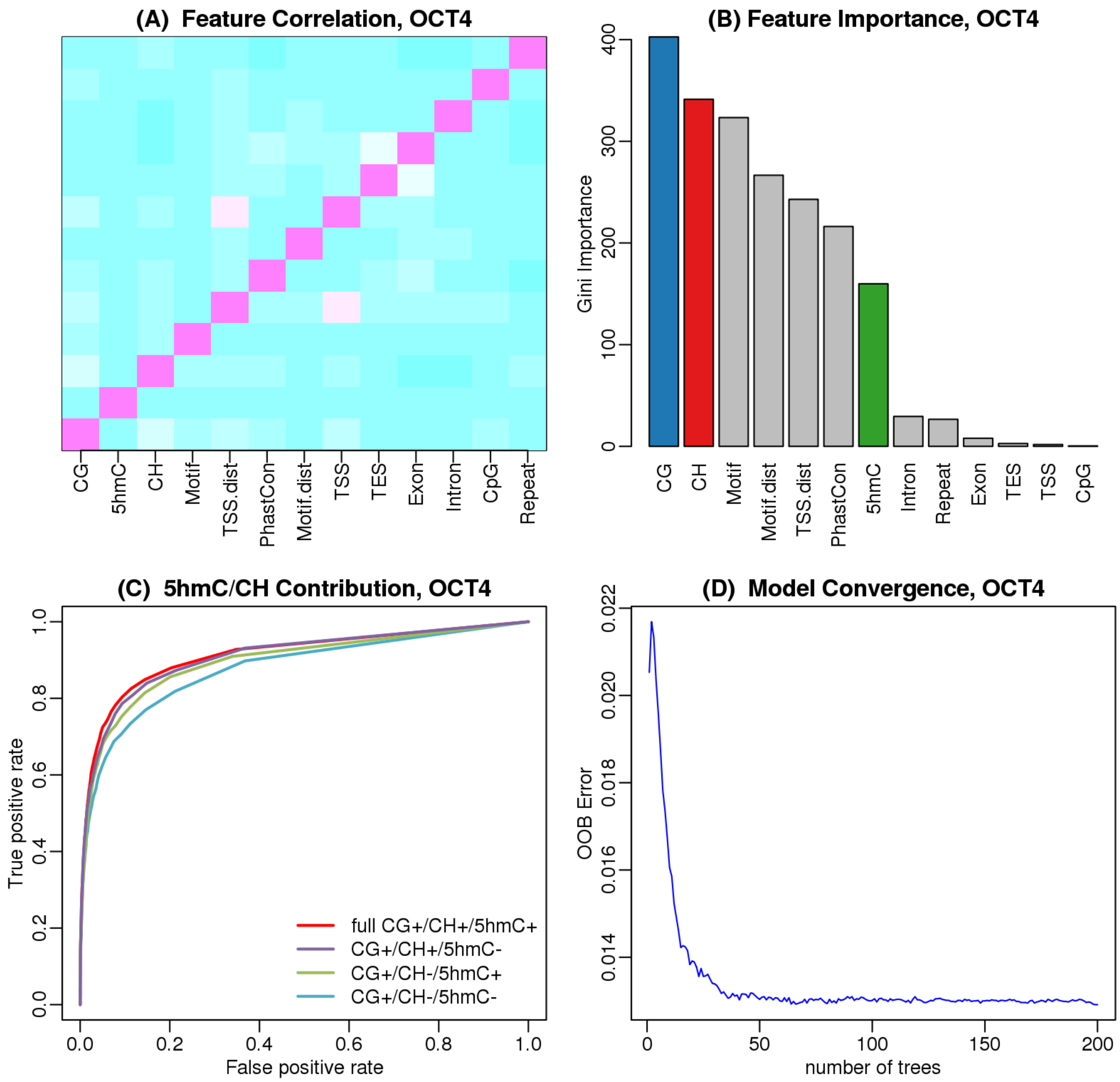

Figure S7-10. Characteristics of features; OCT4 in H1-hESC.

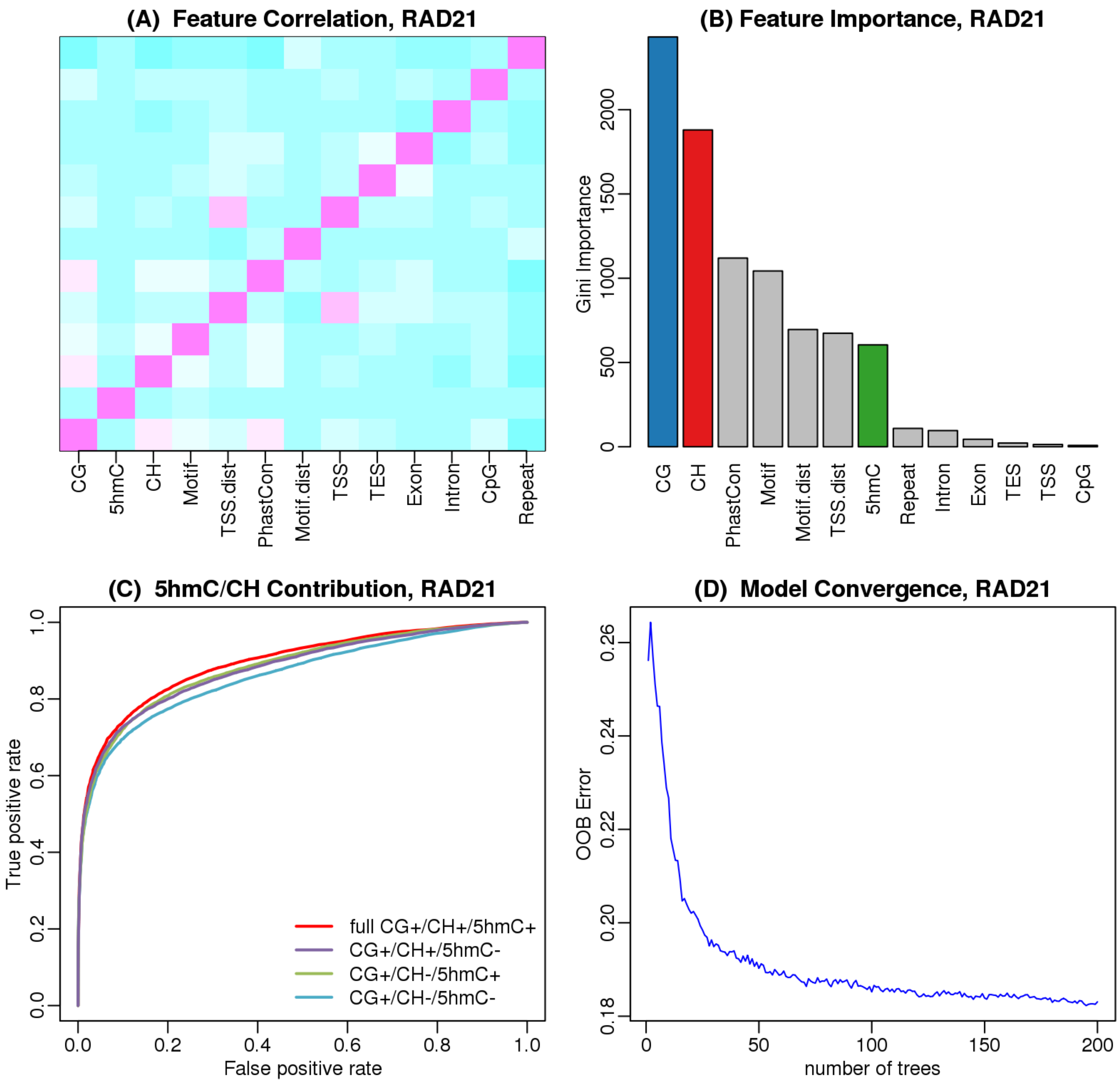

Figure S7-11. Characteristics of features; RAD21 in H1-hESC.

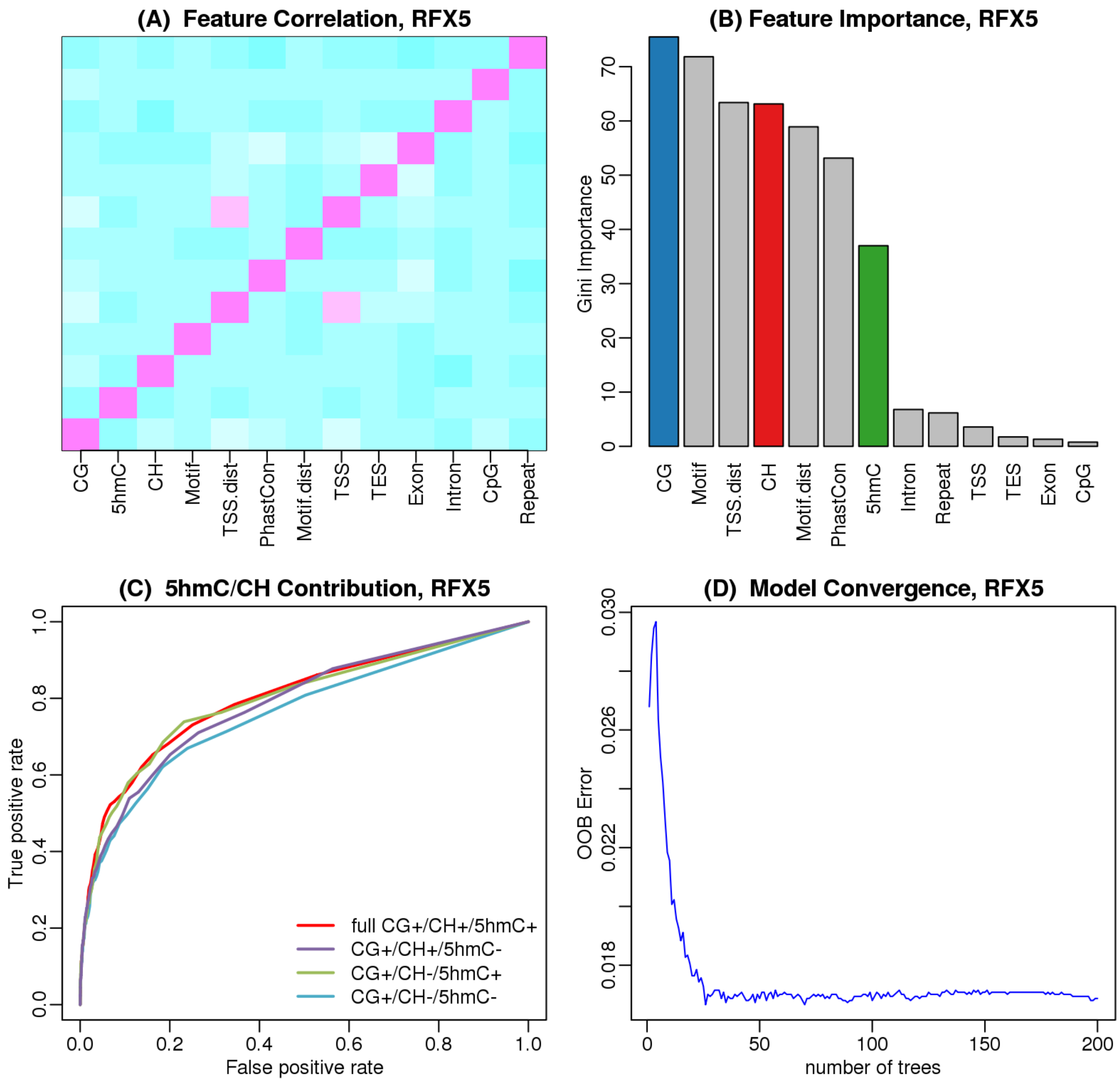

Figure S7-12. Characteristics of features; RFX5 in H1-hESC.

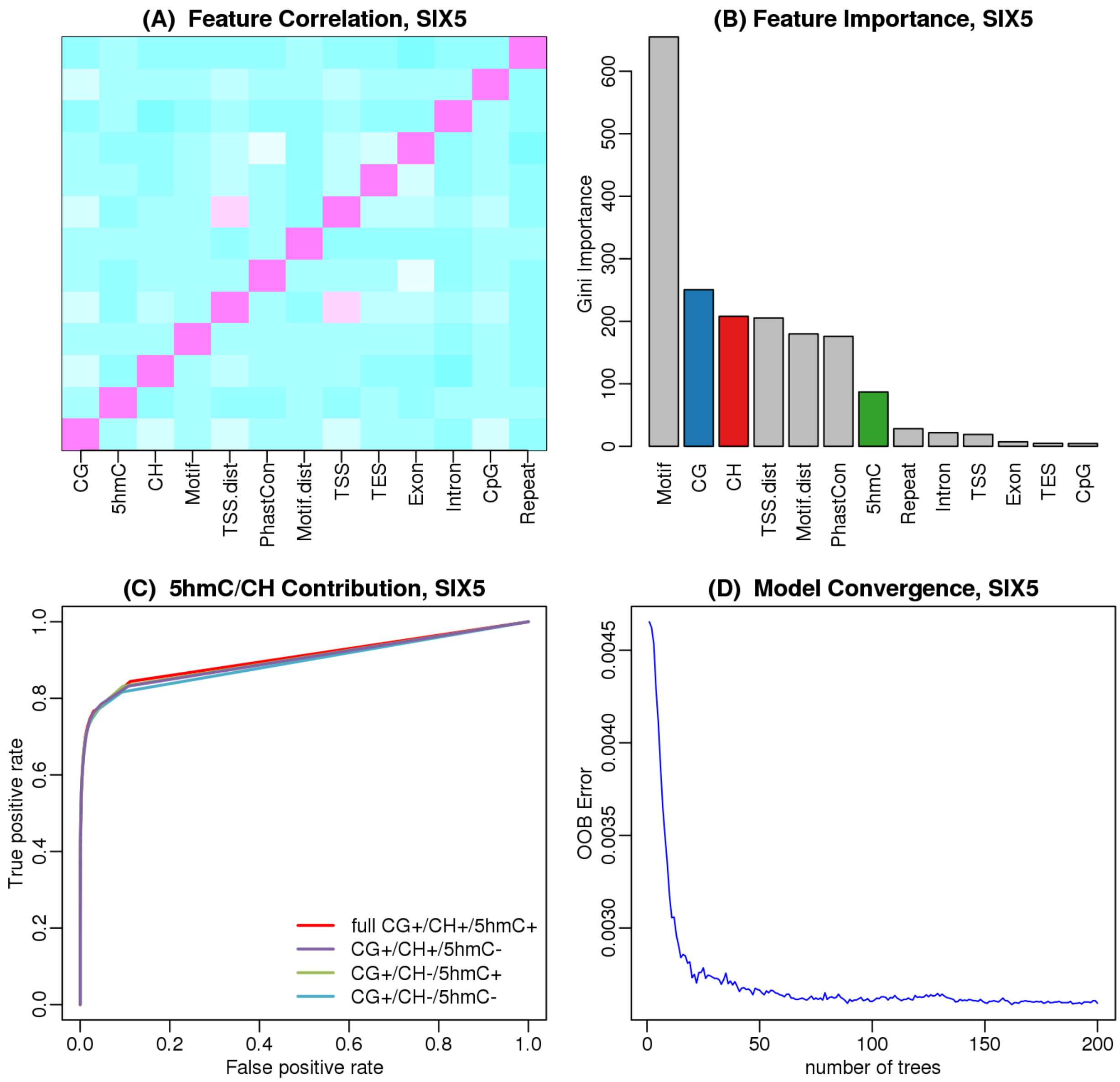

Figure S7-13. Characteristics of features; SIX5 in H1-hESC.

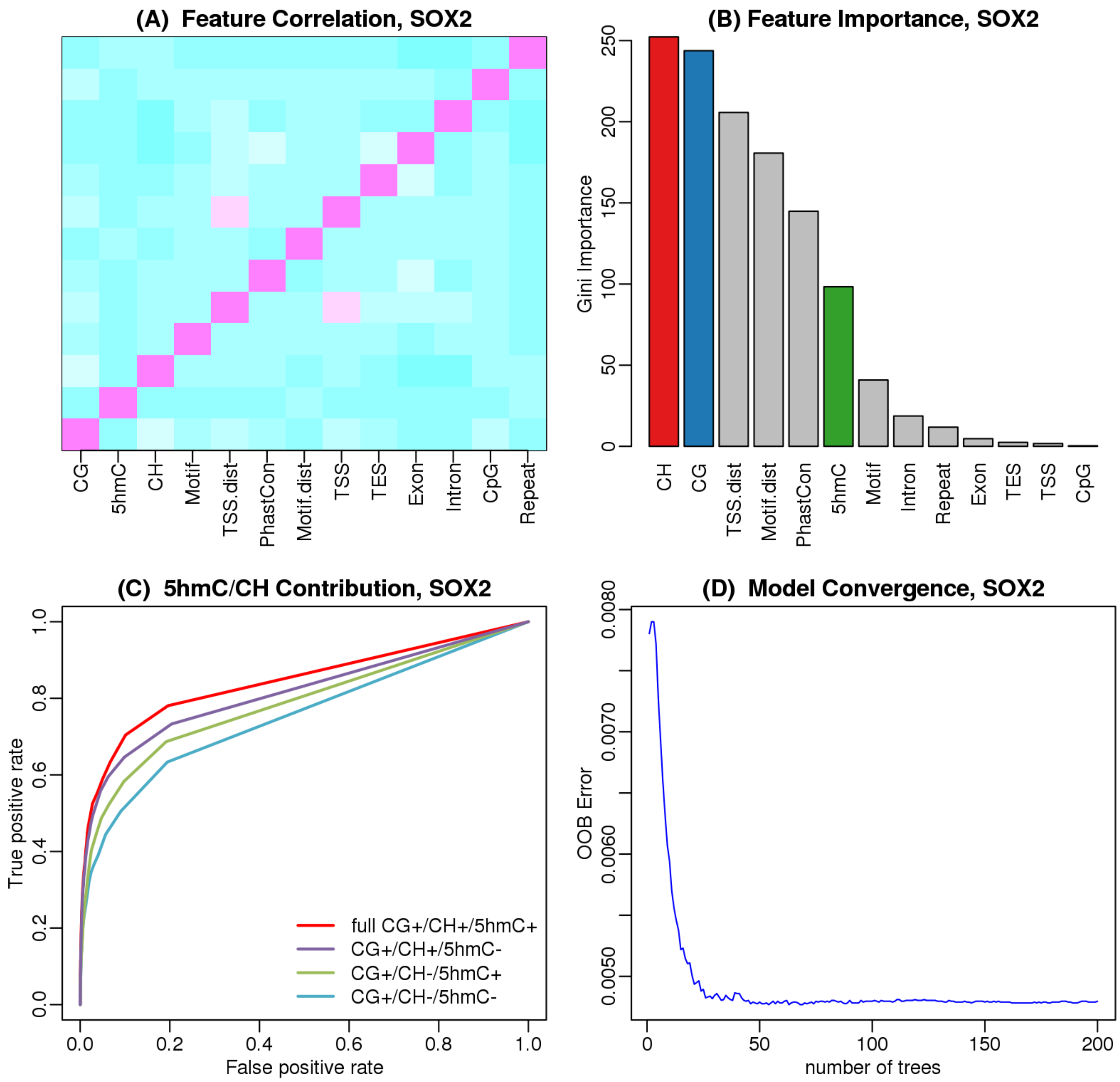

Figure S7-14. Characteristics of features; SOX2 in H1-hESC.

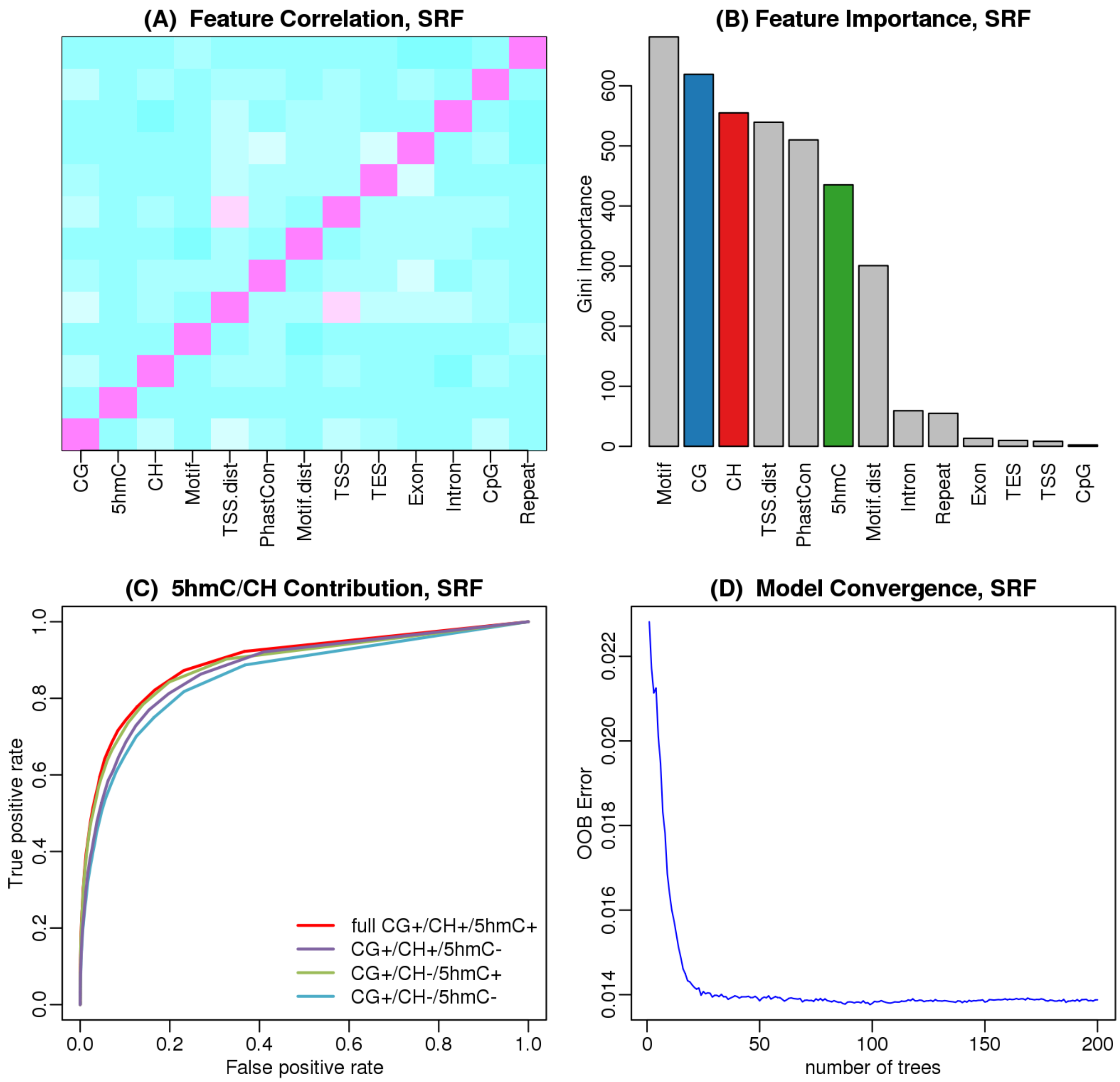

Figure S7-15. Characteristics of features; SRF in H1-hESC.

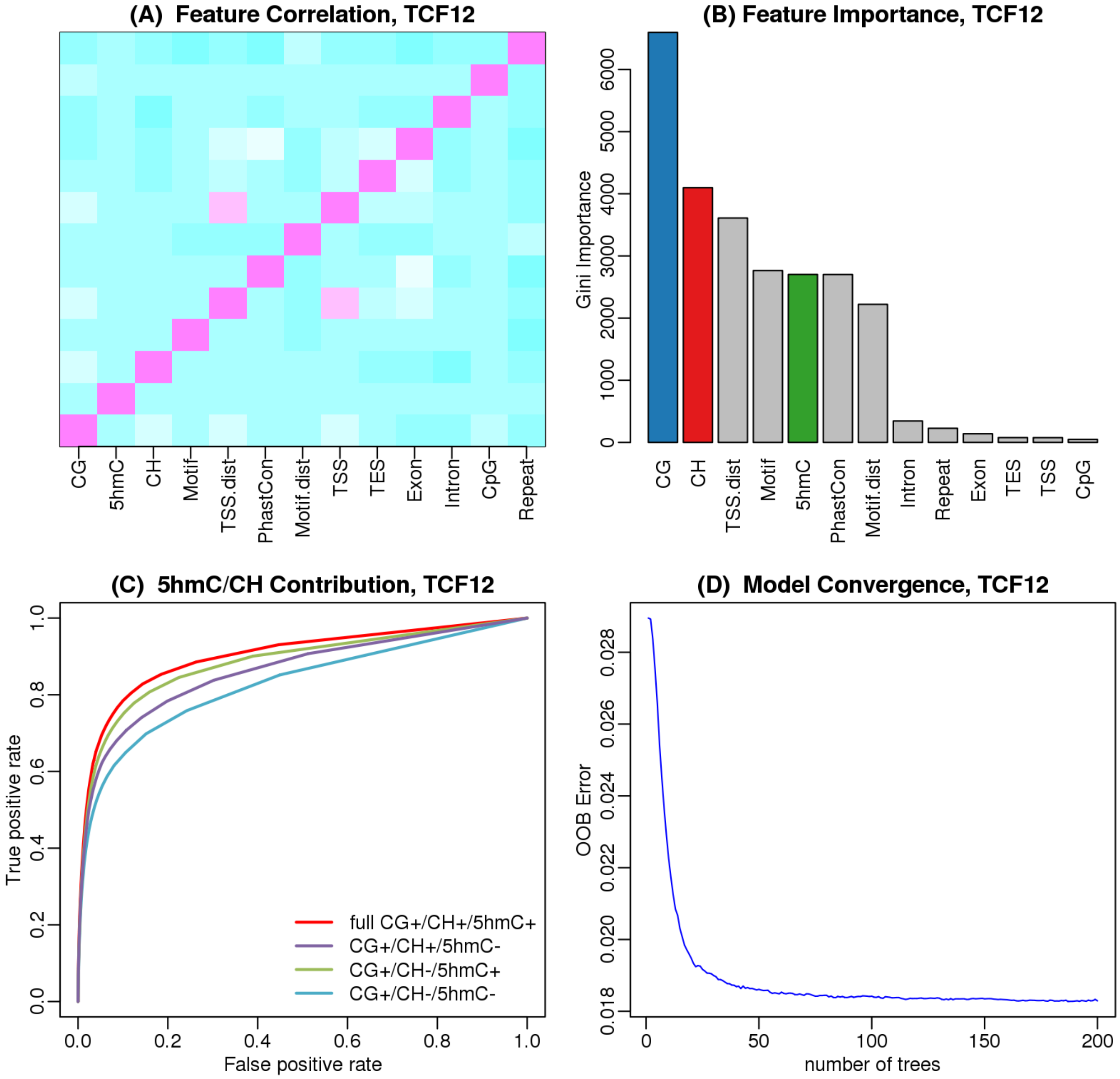

Figure S7-16. Characteristics of features; TCF12 in H1-hESC.

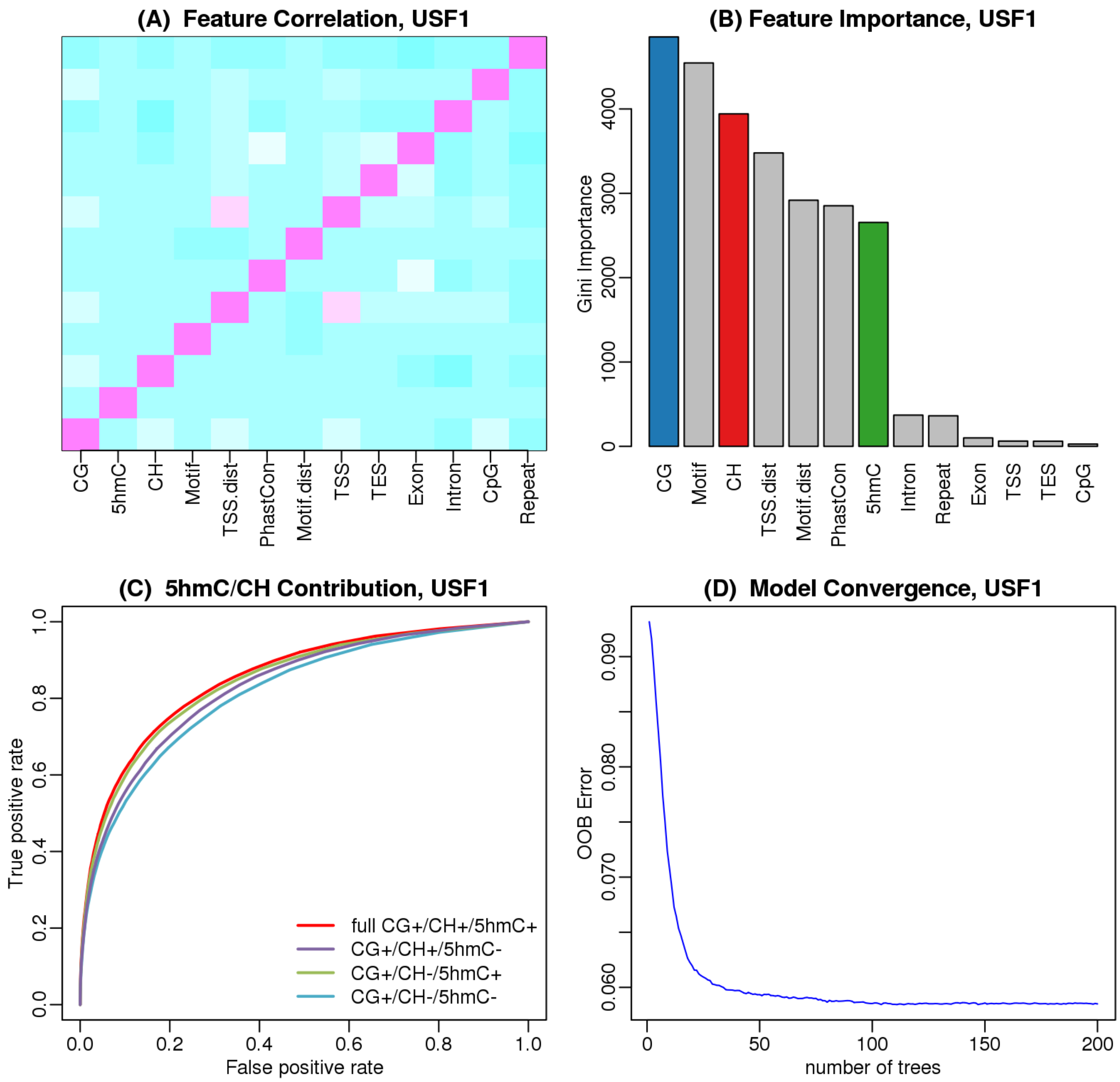

Figure S7-17. Characteristics of features; USF1 in H1-hESC.

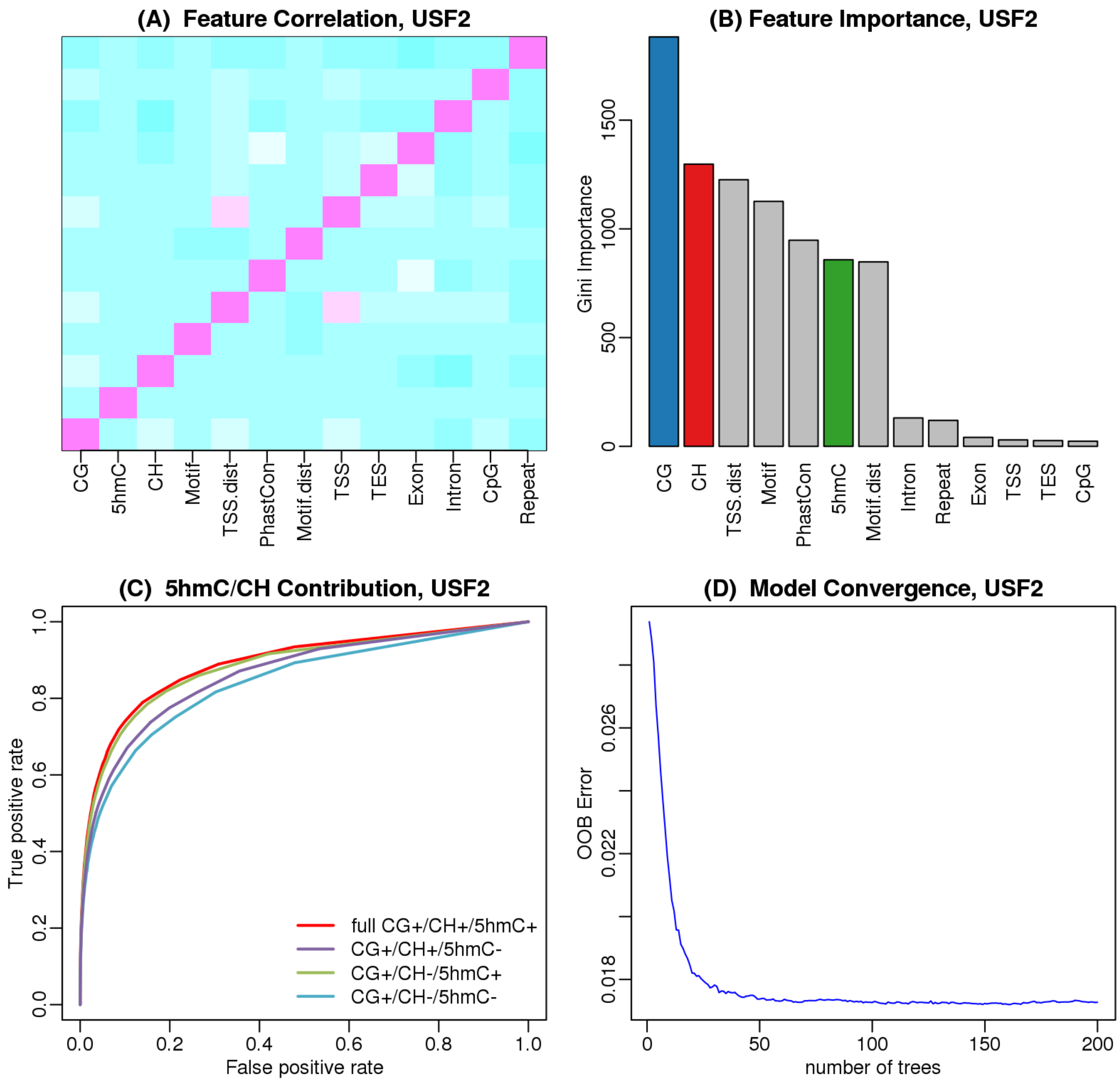

Figure S7-18. Characteristics of features; USF2 in H1-hESC.

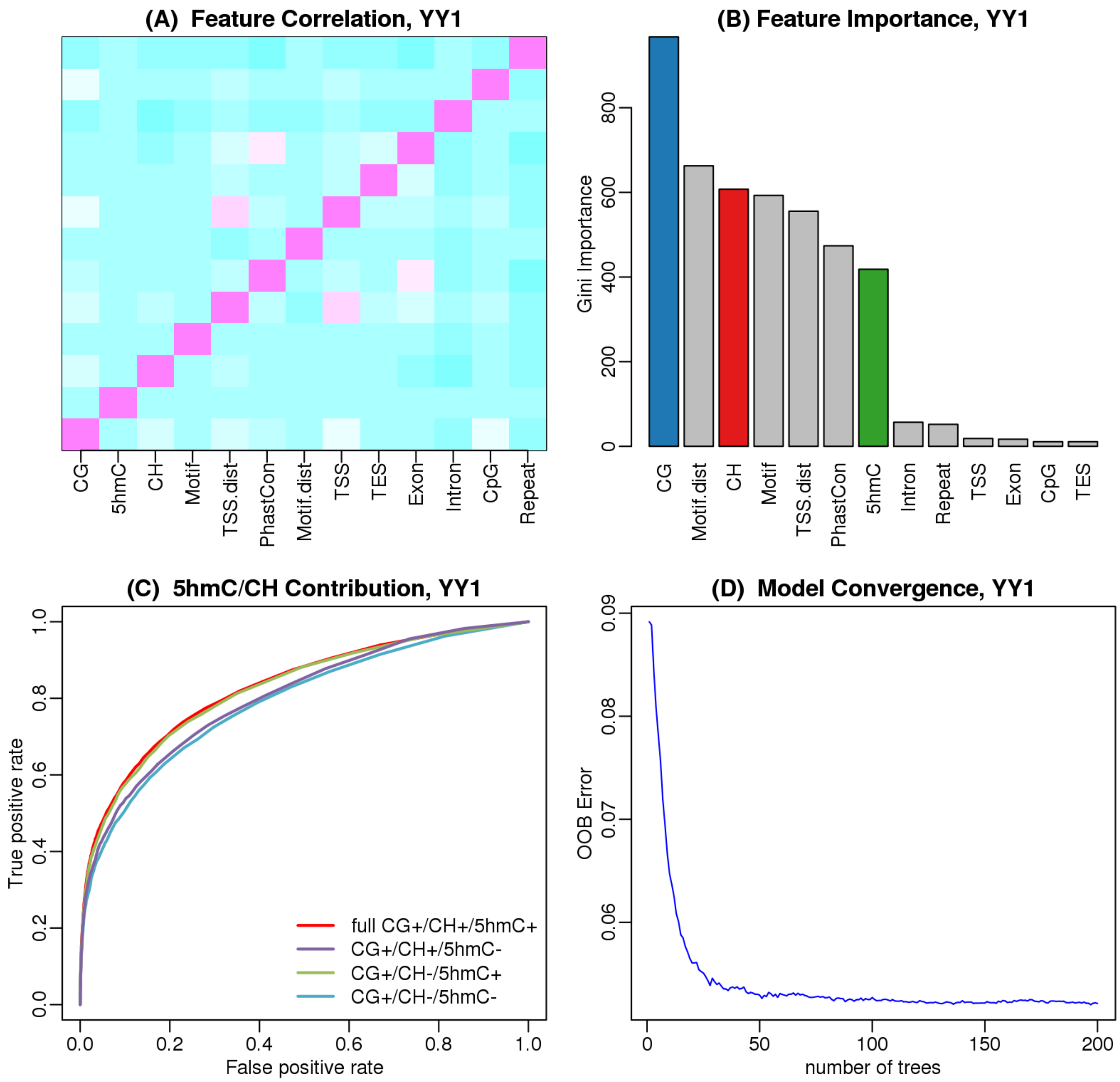

Figure S7-19. Characteristics of features; YY1 in H1-hESC.

7. Comparison with other machine learning algorithms

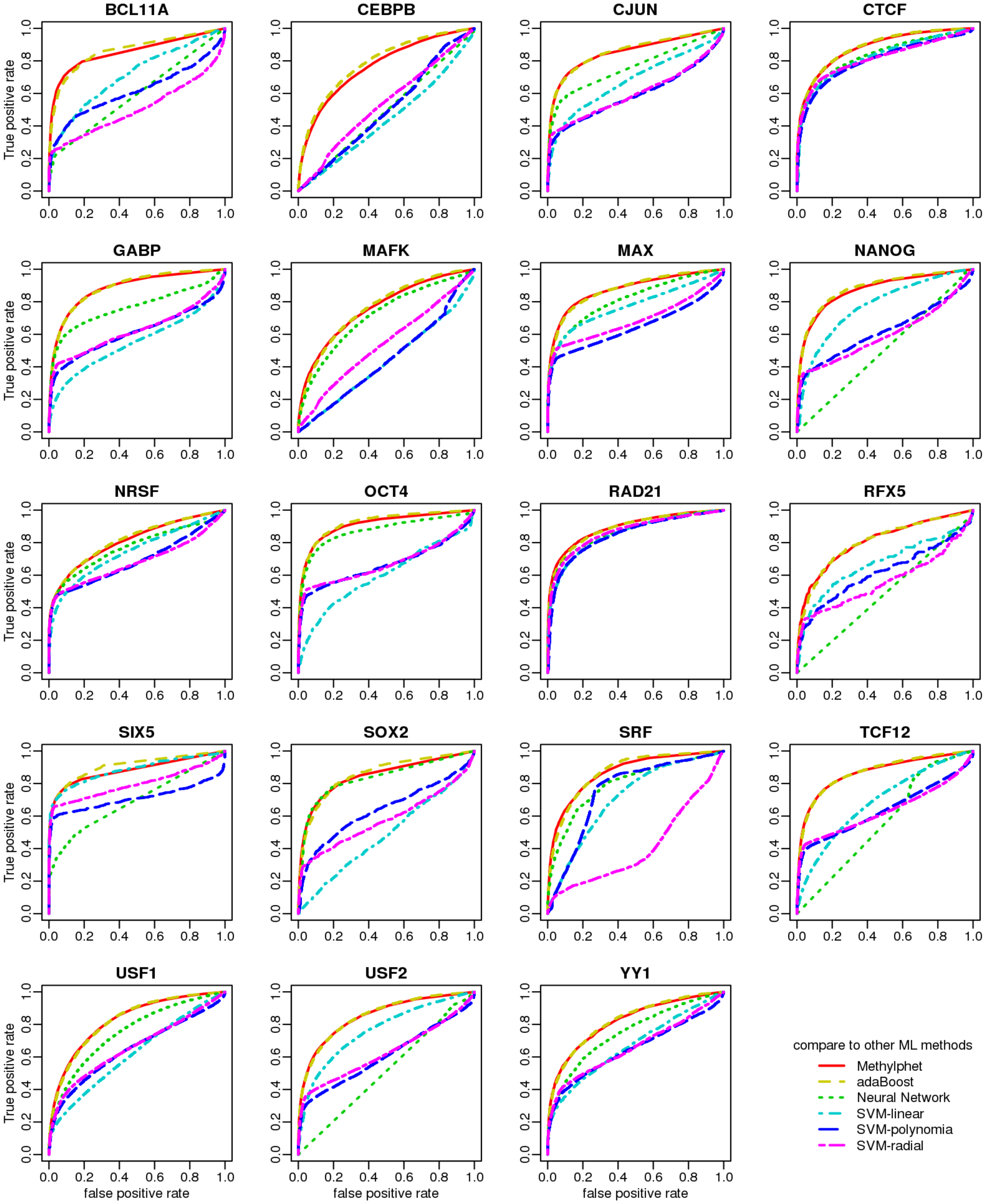

Figure S8. Comparison of results between Methylphet and other popular machine learning (ML) methods, including: adaBoost, Neural Network, SVM (with linear, polinomia and radial kernel).

8. Model evaluation

8.1 Precision-recall curves v.s. ROC curves

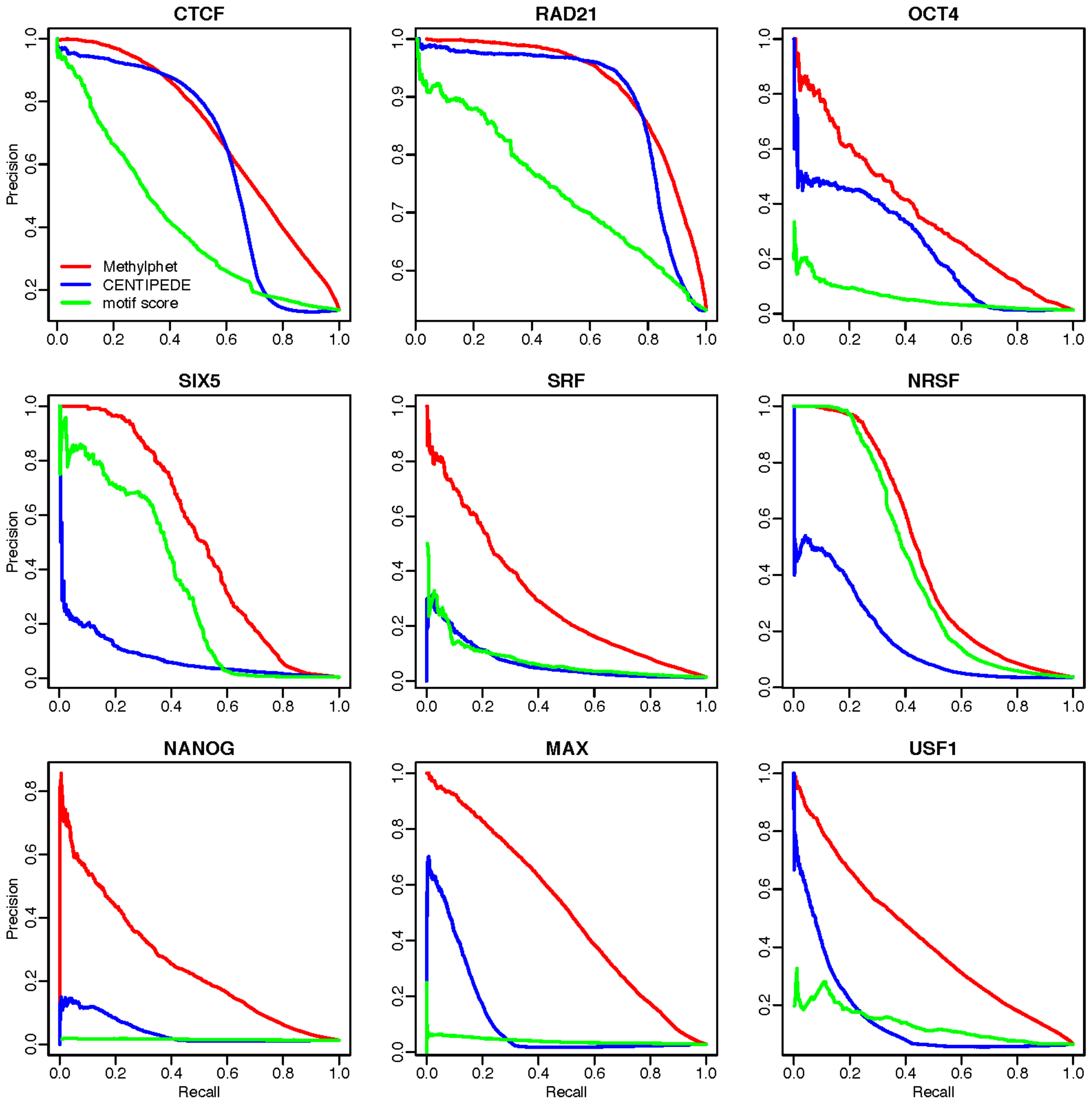

Figure S9. Precision-recall curves for prediction results in H1-hESC cells.

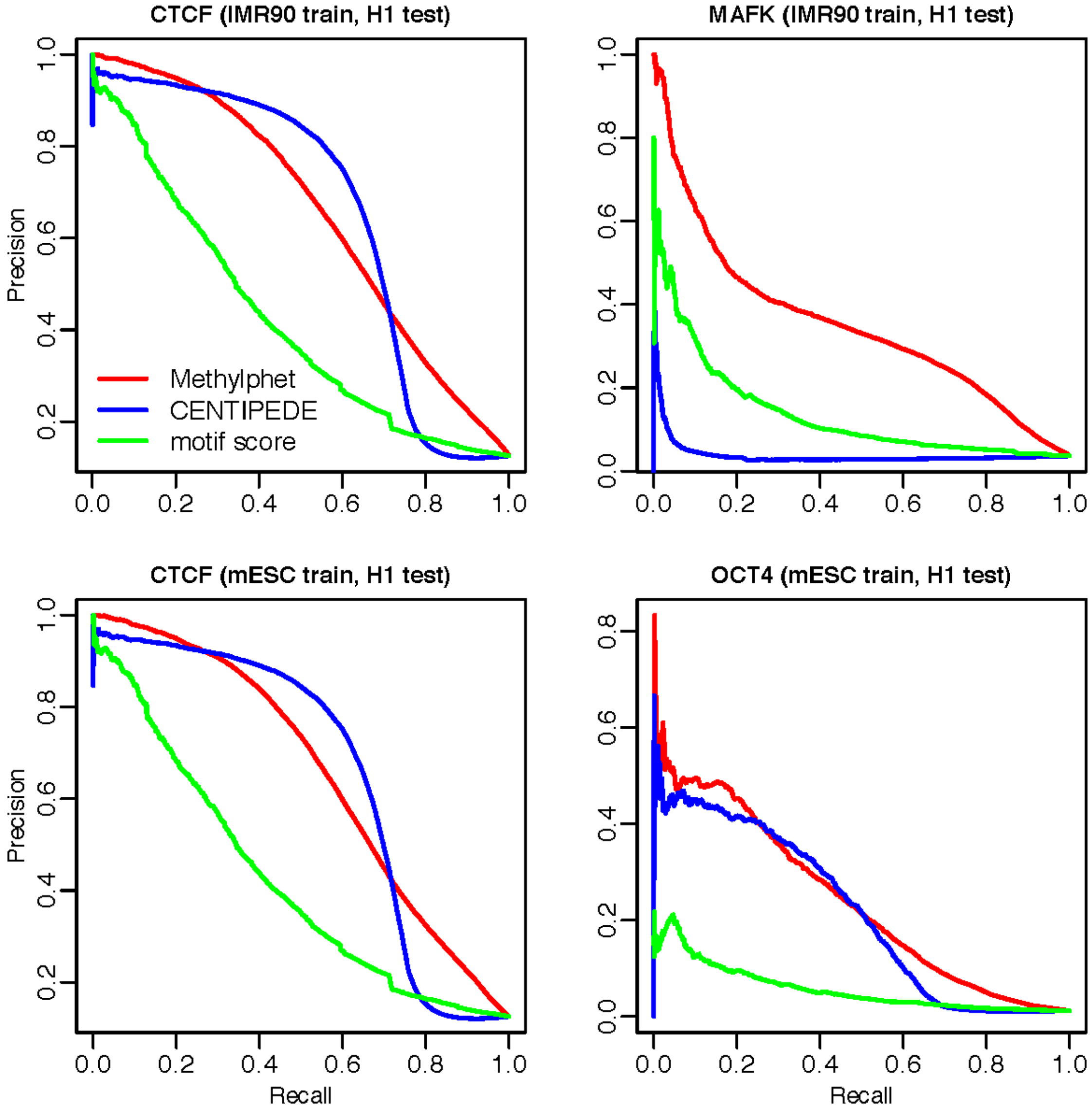

Figure S10. Precision-recall curves for cross-cellline prediction (top two panels: trained in IMR90, test in H1-hHESC; bottom two panels: trained in mESC, test in H1-hESC)

8.2 Choice of bin sizes in constructing methylation model

In constructing methylation model, we take a 300-bp window at each candidate sites (centered at the center of the motif) and divide them into 30-bp windows. The data in the windows are used to estimate methylation patterns. The window size is configurable in the software and can be specified by the users. We set the default window size to be 30-bp because it provides a good balance of the model estimation precision and spatial resolution of the results based on the data we tested.

Smaller window could lead to high uncertainty in model estimation due to potential lower coverage of CpG sites. On the other hand, larger window will hurt the spatial resolution of the results. If the read coverage of the data are deep, one can afford to use a smaller window because there will be enough reads to cover the CpG sites. Otherwise, using a relatively larger window can provide more stable results at the cost of sacrificing a little spatial resolution. We compared the prediction results from Methylphet using 30 bp and 20 bp windows for a number of proteins. Both the ROC and precision recall curves are provided. In general, using 30 bp provides slightly better results overall.

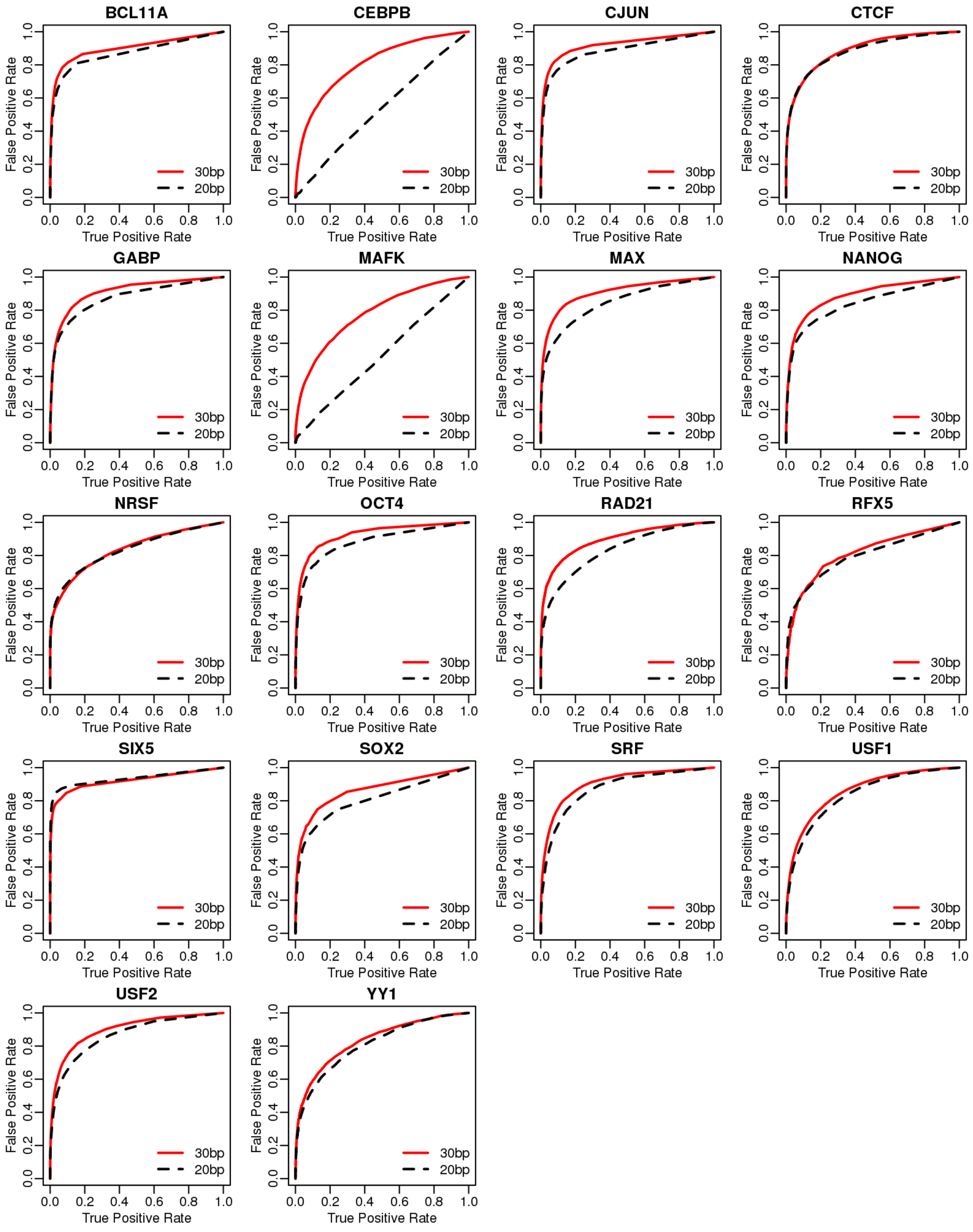

Figure S11-1. Comparison of results using 20bp versus 30bp bins in methylation model. Figure S11-1 shows ROC curves.

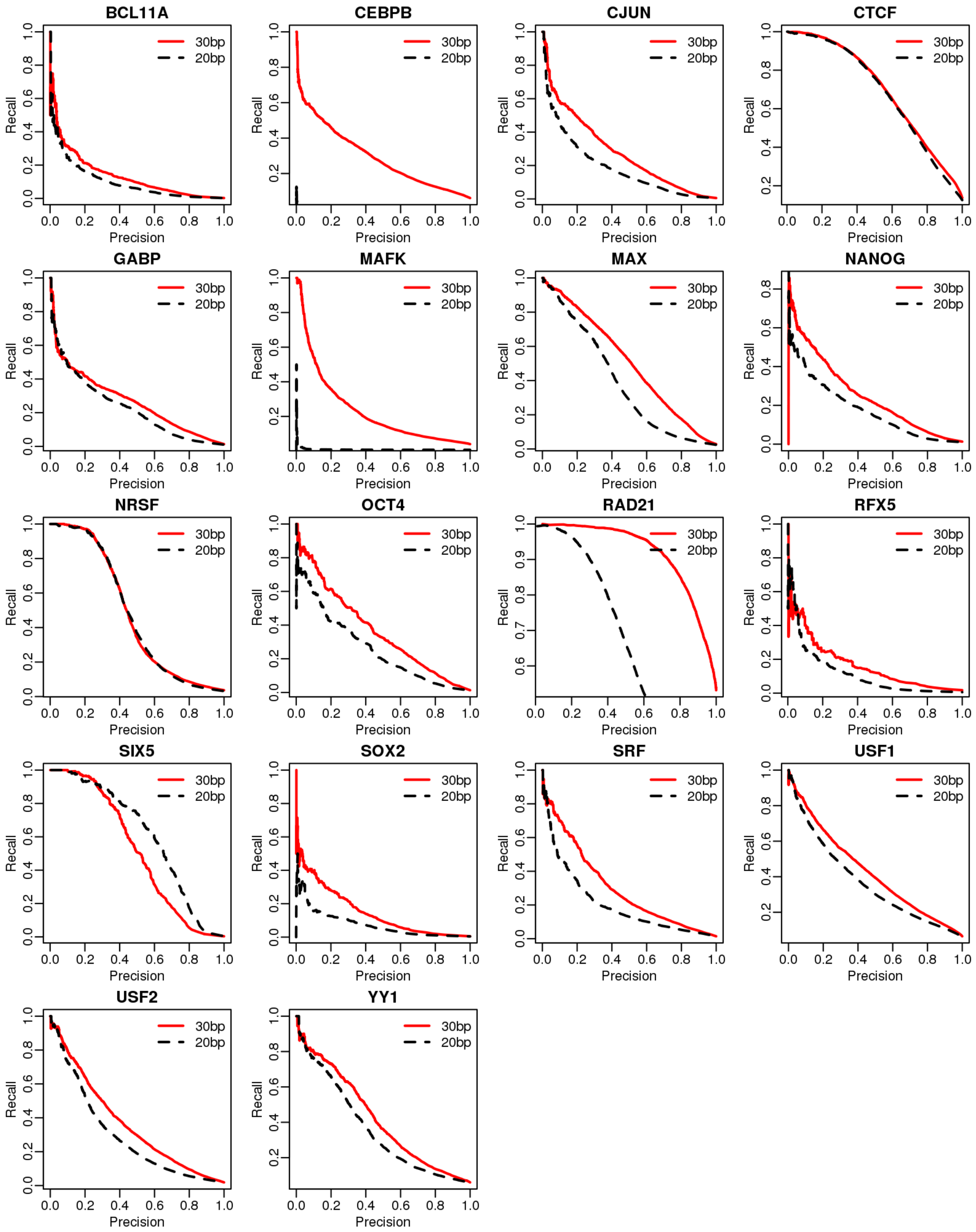

Figure S11-2. Comparison of results using 20bp versus 30bp bins in methylation model. Figure S11-2 shows precision-recall curves.

8.3 AUC result for cross-validation

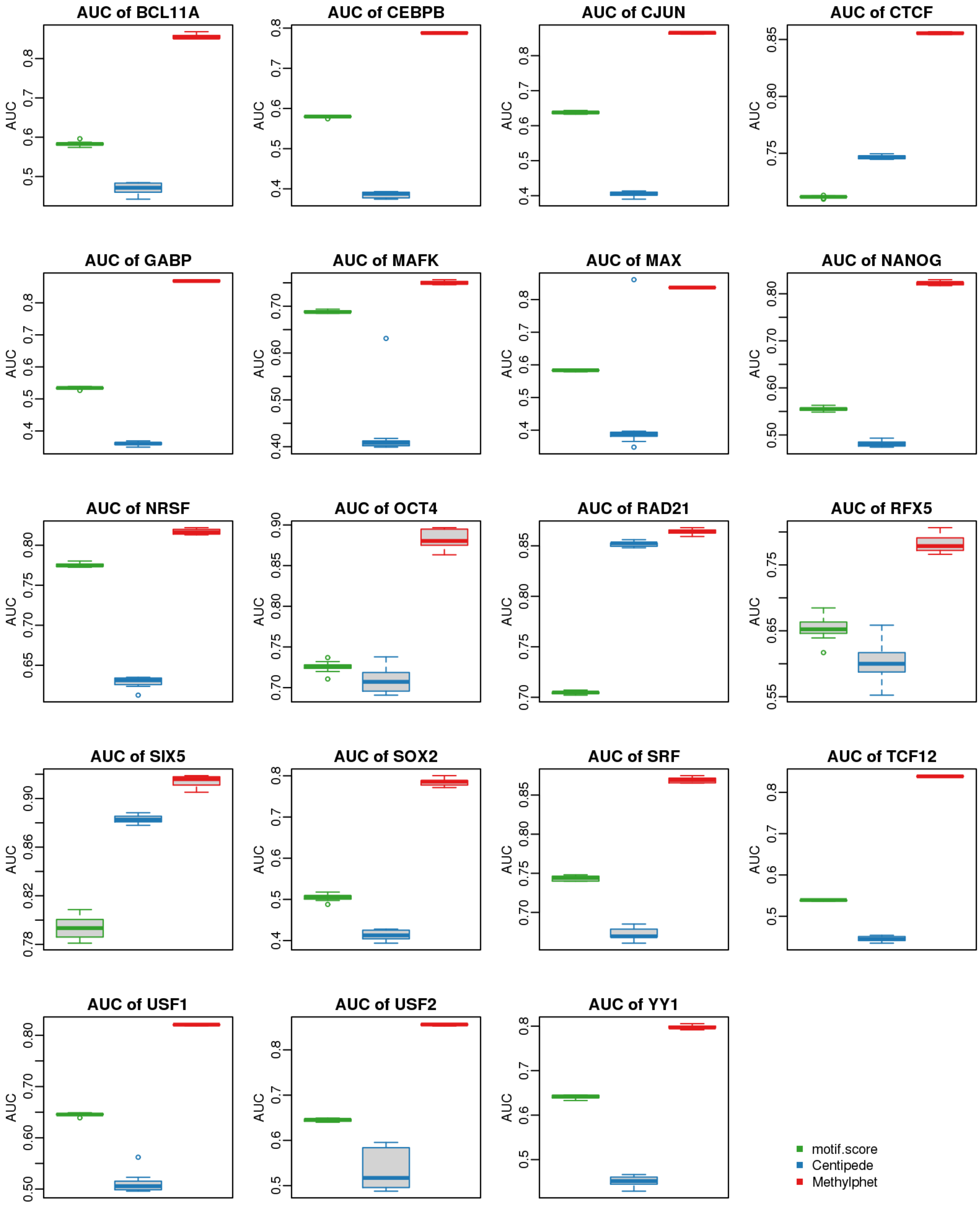

Figure S12. Boxplots of AUC from cross-validation for 19 TFs in H1 cell.

8.4 Comparison of different cell-type-specific features

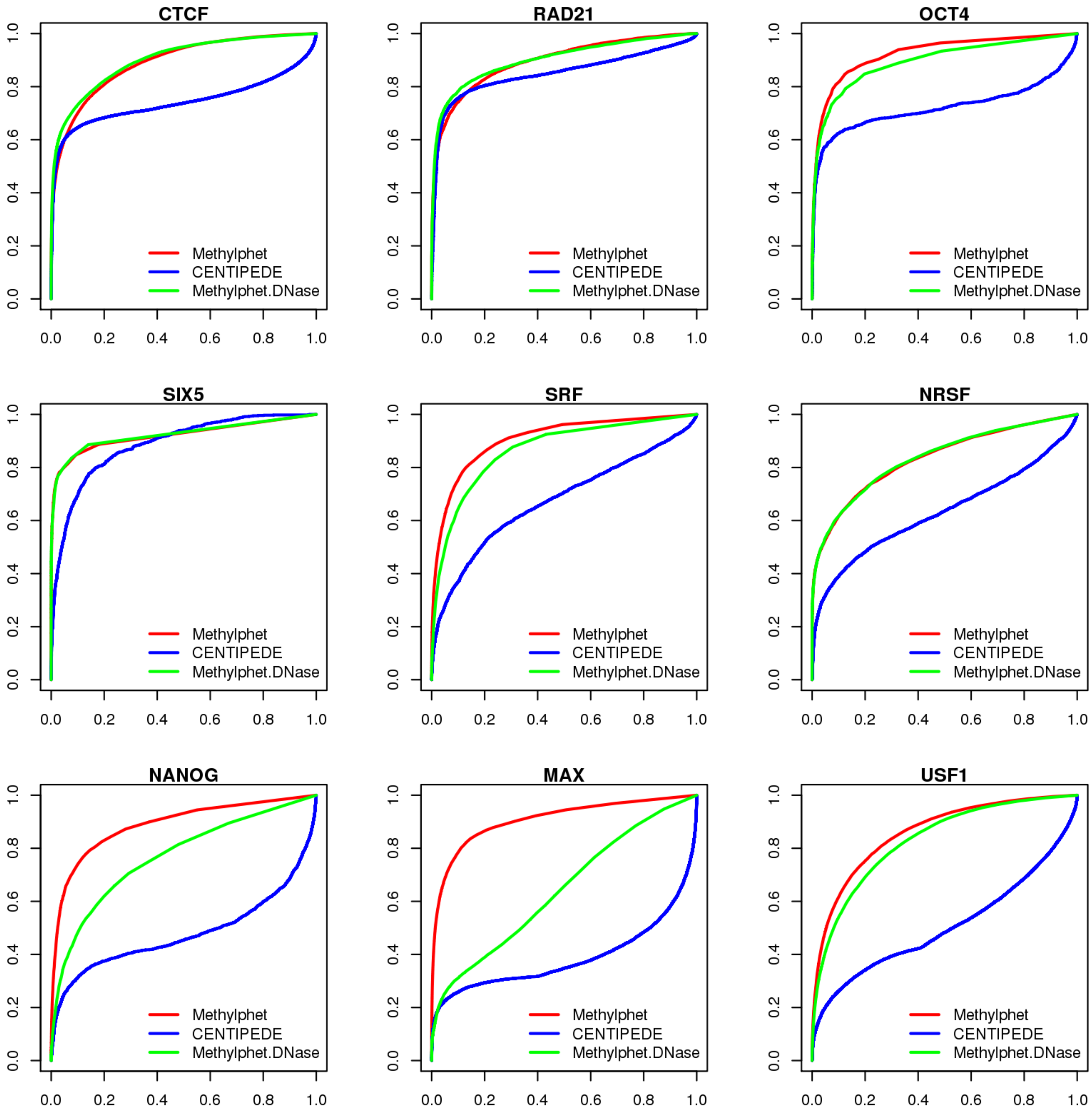

Figure S13. Comparison of ROC curves of (1) Methylphet: red curve; (2) Methylphet using DNase data instead of methylation: green curve; (3) CENTIPEDE: blue curve.

8.5 qPCR of selected Methylphet-predicted sites

qPCR was performed on the selected positive and negative site from Methylphet prediction. The specificity of qPCR reaction for selected sites was confirmed by agarose gel electrophoresis.

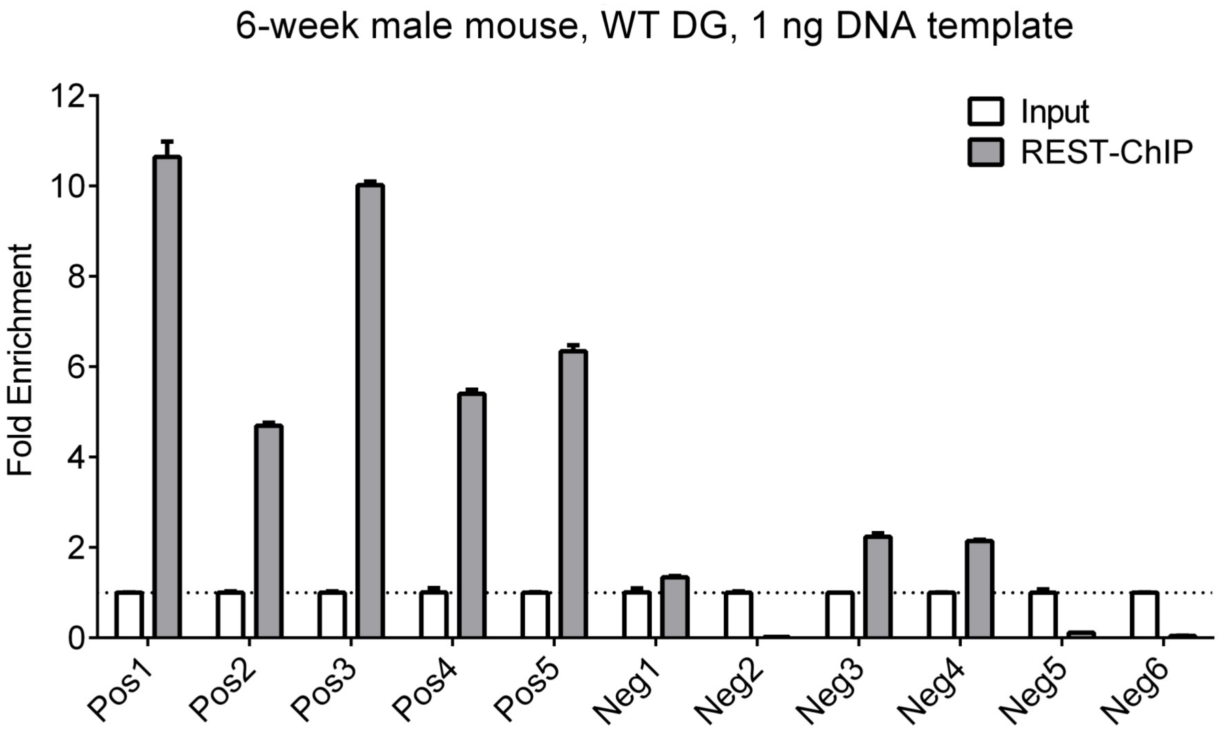

Figure S14. qPCR of selected Methylphet-predicted sites. DG, dentate gyrus; REST=NRSF; Pos, predicted positive site; Neg, predicted negative site. qPCR was performed in duplicates; error bars=std. error

Table S3. Information of selected positive and negative sites

Region #	Locus	Start	End	Amplicon (bp)	Primers (T_M=61-63 dg C)
**Pos1**	Vstm2l	166<br>262	187<br>242	**97**	For: GCCTCTAACATGCAACACCTAG<br>Rev: TCCCTATGCCCCTTTCAGATA
**Pos2**	Gm16532	169<br>251	188<br>231	**83**	For: TTTCAGAACCGTGGACACTG<br>Rev: ACTCAACCATCTCAGAAACGG
**Pos3**	Scamp5	175<br>238	192<br>216	**64**	For: TCGTCCAGGTGCTAGGTG<br>Rev: GTTTCTCCTTTCTATGCACTCAC
**Pos4**	Chga	138<br>212	156<br>192	**75**	For: GATTCAGCACCTCGGACAG<br>Rev: GCGACCATAGCCAACATTATG
**Pos5**	Kcnk9	181<br>280	204<br>261	**100**	For: GGGTCCTGAAATCTCTATGACTTC<br>Rev: TTGCCATTTCAAACCTGCTG
**Neg1**	on chr3	30<br>128	50<br>100	**99**	For: CCTTCATGGACATTTGCACTC<br>Rev: CTTTCTCTTTTTGAGGAGTTACTTTTATC
**Neg2**	on chr6	219<br>287	239<br>266	**69**	For: ACAGAAGTTTTCCTCAGAGCC<br>Rev: TGCTTCTATTCCAGAGTGATGG
**Neg3**	on chr18	23<br>110	44<br>87	**88**	For: GCTCAGAGATGACTTGAACAGG<br>Rev: TCCTACAGTAATTTGCTACCCATG
**Neg4**	on chr5	159<br>248	181<br>229	**90**	For: TCTTGTCTAACTCATGGTTCTGG<br>Rev: ATCTTGGGACTGTGCAGAAG
**Neg5**	on chr1	107<br>193	128<br>174	**87**	For: CACTTTTATAAATGGCGCAGGG<br>Rev: TTAACTTCTCCAGGGCTTGC
**Neg6**	on chr7	60<br>155	79<br>133	**96**	For: AGTGTTGCAGTTGGTAAGGG<br>Rev: ACTCCCAACTTACCTTTCACATC

8.6 Cross-TF prediction result.

To investigate the TF-specificity of Methlphet model, we conducted cross-TF training. We firstly trained Methylphet using methylation data and other features of 9 TFs in H1-hESC as training set. Then we use these models to predict CTCF binding in IMR90 cell line. The ROC curves of this cross-TF cross-cell line prediction are shown in Figure S15.

From this result we see that the CTCF model showed better performance than all of the other models. In addition, RAD21 model shows up as a close second which is reasonable because RAD21 has very similar motif pattern as CTCF. Models trained from TFs other than CTCF and RAD21 perform much worse.

This result clearly demonstrates the TF-specificity of the Methlphet model. The methylation profile and other genomic characteristics of TF binding are important in the Methylphet model and better to be modeled in a TF-specific manner.

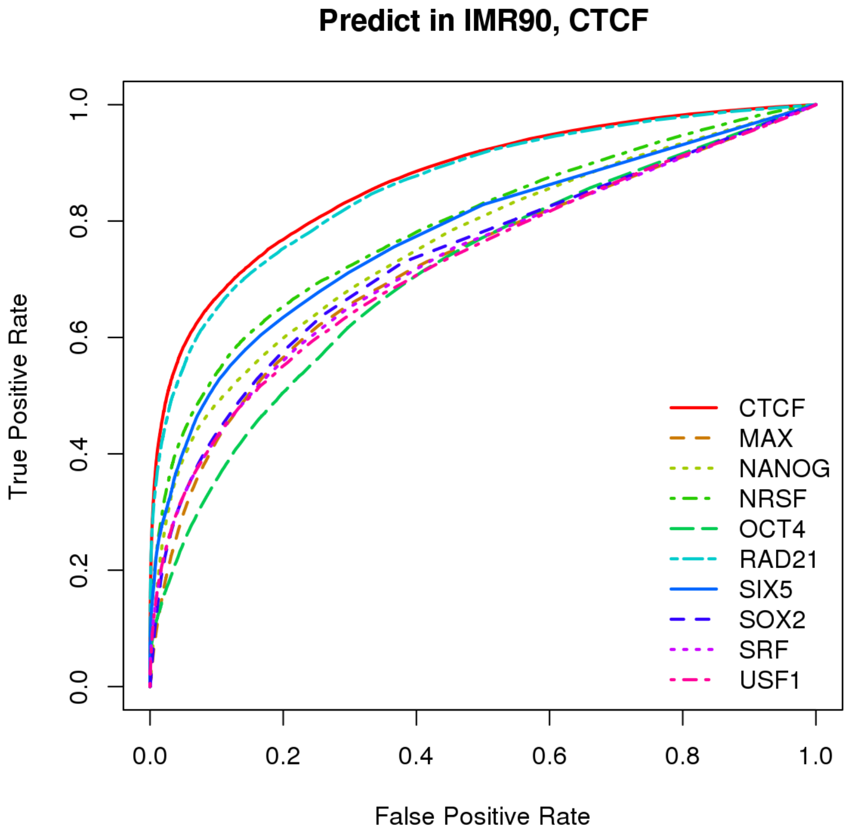

Figure S15. ROC curves of Cross-TF-trained models, with H1-hESC data as training set, IMR90 data as testing set.
